# Chirality in rotaxanes and catenanes

**DOI:** 10.1039/c8cs00097b

**Published:** 2018-05-24

**Authors:** E. M. G. Jamieson, F. Modicom, S. M. Goldup

**Affiliations:** a Chemistry , University of Southampton , University Road, Highfield , Southampton , SO17 1BJ , UK . Email: s.goldup@soton.ac.uk

## Abstract

Although chiral mechanically interlocked molecules (MIMs) have been synthesised and studied, enantiopure examples are relatively under-represented in the pantheon of reported catenanes and rotaxanes and the underlying chirality of the system is often even overlooked. This is changing with the advent of new applications of MIMs in catalysis, sensing and materials and the appearance of new methods to access unusual stereogenic units unique to the mechanical bond. Here we discuss the different stereogenic units that have been investigated in catenanes and rotaxanes, examples of their application, methods for assigning absolute stereochemistry and provide a perspective on future developments.

## Abstract 2

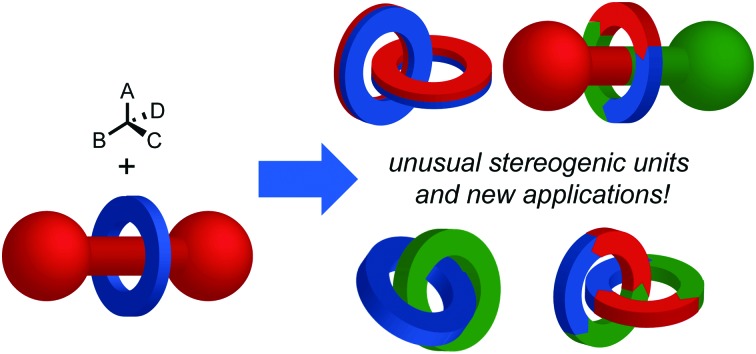
We discuss the stereogenic units that have been investigated in interlocked molecules, their application, absolute stereochemistry and propose future directions.

## Introduction

**Fig. 1:**
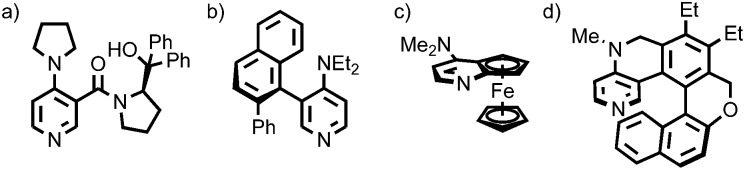
Examples of functional molecules containing chiral covalent (a) point,^6^ (b) axial,^7^ (c) planar^8^ and (d) helical^9^ stereogenic elements.

Chirality is one of the central topics in chemistry. Indeed, ∼10% of publications in general chemistry journals in 2016 related to the topic in some way.^1^ The modern importance of chiral molecules stems largely from the homochirality of biological systems and the associated need to generate molecules in enantiopure form for biological applications. However, historically chirality was also central to development of theories of chemical structure; the observation of optical activity in sodium ammonium tartrate crystals by Pasteur^2^ ultimately led to the independent realisation that the substituents of a tetravalent carbon centre occupy the vertices of a tetrahedron by Le Bel and Van’t Hoff.^3^ Since then, the recognised chiral covalent stereogenic units have been expanded to encompass axial, planar and helical motifs, in addition to stereogenic centres,^4^ all of which have been brought to bear on chemical problems, and the absolute stereochemistry of which (with the exception of helical) are assigned using variations of the systems originally developed by Cahn, Ingold and Prelog (Fig. 1).^5^

Given the foundational role chirality played in chemistry and the modern focus on the applications of chiral molecules, it is perhaps surprising that chirality has played a more limited role in the field of mechanically interlocked molecules (MIMs)^10^ such as rotaxanes and catenanes. Indeed, in the last decade only ∼3.5% of publications in the area explicitly mention chirality.^11^ Nevertheless, chirality in interlocked molecules has been shown to be a worthwhile area of study, yielding enantioselective hosts for small molecules, materials with unusual optical properties and catalysts for enantioselective transformations, many of which exhibit behaviour that intrinsically depends on the mechanical bond.^12^ Moreover, interlocked molecules may display chirality as a direct result of the mechanical bond thanks to stereogenic units that are not found in other molecules, and these remain dramatically under-explored.^13^

In this review, we highlight the interplay between mechanical bonding and chirality. We start by discussing selected examples of interlocked molecules that contain covalent stereogenic units with a focus on their applications. We then move on to discuss examples of chiral MIMs in which the mechanical bond itself plays the role of stereogenic unit. In each case we describe the stereogenic unit and discuss methods for assigning their absolute stereochemistry. In some cases, particularly where multiple methods of assignment have been presented, where no clear method exists, or where the published method is limited in scope, we have made suggestions for how absolute stereochemistry can be assigned in a consistent manner across the range of possible structures.

In preparing this Review we found it necessary to consider the taxonomy of stereogenic units that arise due to the mechanical bond in order to present the material logically. The discussion of stereogenic units in MIMs is divided into covalent, mechanical, co-conformational and unconditional. It should be noted that in places our taxonomy differs from other authors’ and where appropriate we have justified our approach while acknowledging previous opinions. Furthermore, in considering the taxonomy of chiral MIMs it also became clear that some mechanical stereogenic units are “missing”. At the end of the review we outline these “missing mechanical stereogenic units” and characterise them based on the underlying symmetry properties of their subcomponents. We conclude with a discussion of future directions, challenges and opportunities in the study and application of chiral interlocked molecules.

### Stereochemical language – precision and imprecision

Stereochemical analysis is a complex topic requiring precise terminology, something that can be overlooked in the practice of synthetic chemistry.^14^ For example, although “chirality” is a whole molecule property, organic chemists routinely refer to “chiral centres/points”, “chiral axes” and “chiral planes” and refer to these collectively as “elements of chirality”. Molecules containing such units are commonly described as “centrally/point”, “axially” and “planar” chiral.

More precisely, these “elements of chirality” are stereogenic units around which, when considered in isolation, the space is chirotopic^14^ and thus, whose presence in a molecular structure can lead to the appearance of molecular chirality. The resulting molecules are simply chiral, chirality itself being a whole molecule property. The obvious demonstration of the error in calling such stereogenic units “elements of chirality” is that molecules can contain them without exhibiting molecular chirality; *meso* compounds typically contain two or more stereogenic units positioned such that they are related by a mirror plane (σ), point of inversion (i) or rotation–reflection axis (S_*n*_) (collectively “improper symmetry operations” or operations of the “second kind”) in at least one available conformation and are thus on average achiral. Indeed, the presence of an improper symmetry operation is a sufficient criterion for the assignment of achirality and *vice versa*.

For the avoidance of doubt, here we use the term “chiral stereogenic element/unit” to describe structural fragments (*e.g.* a carbon centre with four different substituents) around which, when considered in isolation, all points are chirotopic,^14^ and thus whose inclusion in a molecule is a sufficient, but not automatic, condition for the emergence of molecular chirality. However, having explained why terms such as “centrally chiral” to describe a molecule are misleading, we propose to use them below for the simple expedient that they are easily understood by the majority of chemists and result in linguistically simple statements. For example a molecule described as “point chiral” is readily understood to contain at least one chiral stereogenic centre and belong to a point group with no improper symmetry operations. Hopefully, having explained the difficulty such language presents from a formal perspective we will avoid confusing the situation further.

## Chiral covalent stereogenic units

It is trivial to imagine connecting an interlocked molecule with a group containing a chiral stereogenic unit or including such a unit as part of the MIM structure itself to generate a chiral interlocked molecule. Furthermore, the keyword searches discussed above almost certainly underestimate the actual number of publications concerning chiral MIMs, given that glucose-derived cyclodextrin macrocycles are widely used in MIM synthesis, although their chirality is often not a significant feature of their properties or applications.^15^ Indeed, it can sometimes be difficult to avoid including chiral covalent stereogenic units in the structure of complex MIMs for synthetic reasons.

However, when chiral covalent stereogenic units are intentionally included in the structure of MIMs, functional molecules can be produced for a range of applications in which their chirality plays a key role. In the following sections we will highlight selected examples of these, with a focus on how the mechanical bond alters their behaviour before moving on to the effects of chiral covalent stereogenic units in MIM synthesis.

### MIMs containing chiral covalent stereogenic units in catalysis

Enantioselective catalysis is one of the dominant applications of chirality in modern chemistry^16^ and by embedding catalytic moieties in the framework of a rotaxane or catenane it is possible to generate interlocked catalysts that present a well expressed chirotopic reaction field which is enhanced or altered by the mechanical bond.

**Fig. 2:**
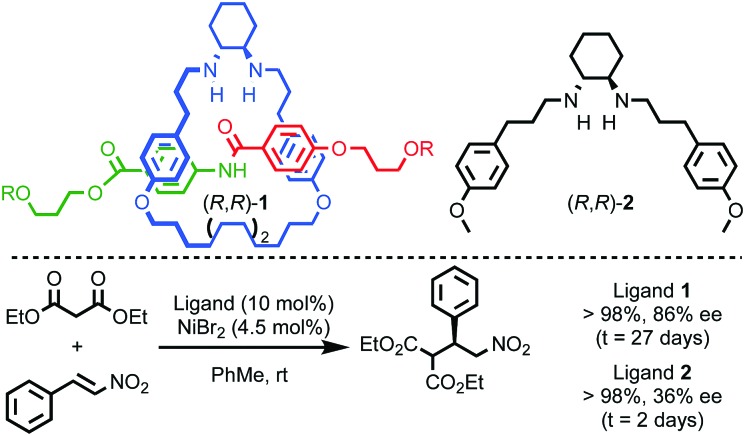
Leigh's chiral rotaxane ligand (*R*,*R*)-**1** in the Ni^II^-catalysed Michael addition.^17^ R = 4-(C_6_H_4_)-C(4-(C_6_H_4_)-^*t*^Bu)_3_.

Leigh and co-workers demonstrated that the crowded environment of the mechanical bond could lead to enhanced enantioselectivity in catalysis (Fig. 2).^17^ The macrocycle in rotaxane (*R*,*R*)-**1** bears two stereogenic carbon centres that are not related by an improper symmetry operation and the molecule is thus chiral. When (*R*,*R*)-**1** is used as a ligand in a Ni^II^-mediated Michael addition reaction the product is produced in higher enantioselectivity than acyclic chiral ligand (*R*,*R*)-**2** alone. This effect is attributed to the more sterically crowded environment of the rotaxane enhancing the reaction stereoselectivity. It should also be noted that the same effect appears to slow the catalytic process dramatically.

**Fig. 3:**
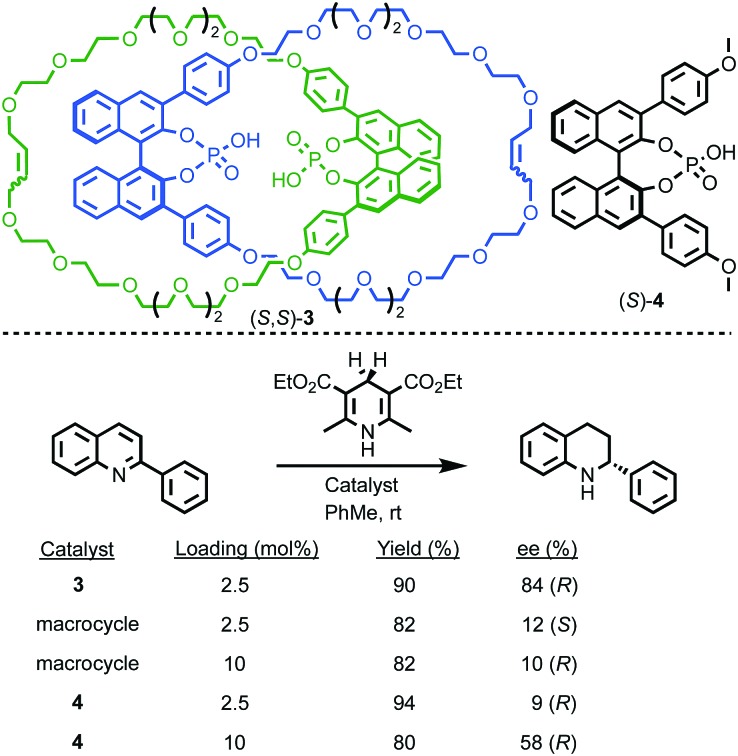
Niemeyer's chiral catenane phosphoric acid (*S*,*S*)-**3** in the transfer hydrogenation of quinolines.^18^

More recently, Niemeyer and co-workers demonstrated extremely high enantioselectivity in the Brønsted acid catalysed transfer hydrogenation of quinolones mediated by bis-phosphoric acid chiral catenane (*S*,*S*)-**3**.^18^ Comparison of the performance of catenane (*S*,*S*)-**3**, the corresponding non-interlocked macrocycle and acyclic ligand (*S*,*S*)-**4** revealed a number of interesting points (Fig. 3). Firstly, catenane (*S*,*S*)-**3** consistently produces higher enantioselectivity than either the macrocycle alone or (*S*,*S*)-**4**, albeit with a lower rate of reaction that, as above, is attributed to the increased steric hindrance of the interlocked structure, as in Leigh's report of rotaxane (*R*,*R*)-**1**. Secondly, acyclic phosphoric acid (*S*,*S*)-**4** displays concentration dependent enantioselectivity, with greater selectivity observed at higher concentrations. Molecular modelling rationalised these observations by invoking intermediates in which a hydrogen bonded dimeric phosphoric acid species activates the substrate. The competing pathway that is monomeric in phosphoric acid was found to deliver lower levels of selectivity. The high effective molarity of the phosphoric acid moiety in the catenane ensures the dimeric pathway dominates in the case of (*S*,*S*)-**3**. Similarly, raising the concentration of (*S*,*S*)-**4** increases the rate of the dimeric pathway relative to the less selective monomeric reaction. According to this rationale, the non-interlocked macrocycle delivers poor enantioselectivity in all cases because the ring structure inhibits the dimeric pathway. Conversely, although sterically hindered, catenane (*S*,*S*)-**3** remains flexible enough to accommodate the more enantioselective mechanism.

**Fig. 4:**
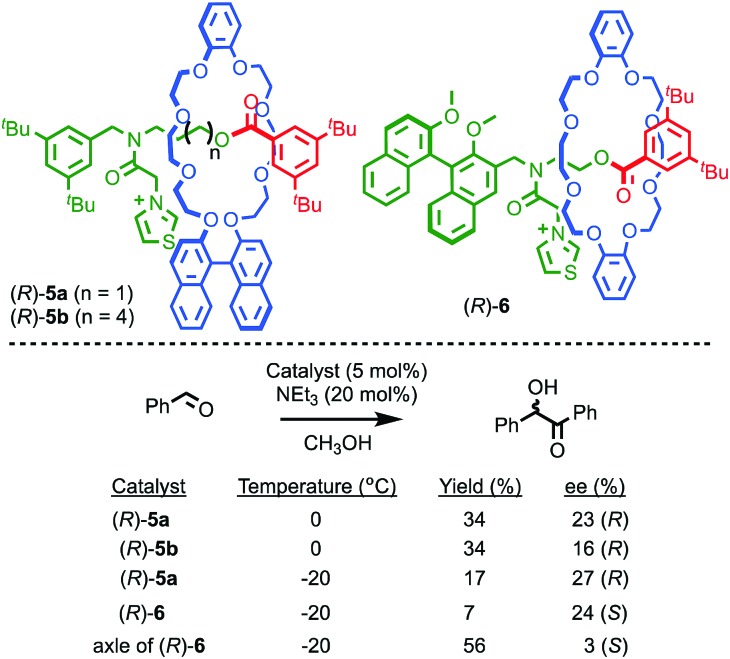
Takata's thiazolium rotaxane catalysts (*R*)-**5** and (*R*)-**6**.^19^ Counter ions omitted for clarity.

It is also possible to impose a chiral environment on an otherwise achiral catalytic fragment using the mechanical bond. Takata and co-workers demonstrated this principle in 2004 using rotaxanes (*R*)-**5** which contain a thiazolium moiety in an achiral axle moiety encircled by a chiral binaphthyl-containing macrocycle (Fig. 4).^19^ The corresponding non-interlocked thiazolium axle of (*R*)-**5a**, deprotonated to form an N-heterocyclic carbene moiety, mediates the formation of a racemic benzoin product. Conversely, when the same reaction is carried out using rotaxane (*R*)-**5a**, an enantioselectivity of 23% ee was obtained. This result demonstrates that the crowded environment of the mechanical bond allows for the relatively efficient transfer of stereochemical information, resulting in a well-expressed chirotopic environment around the catalytic functionality. Lengthening the axle, as in rotaxane (*R*)-**5b**, results in diminished enantioselectivity, as would be expected from the decreased crowding between the chiral macrocycle and the thiazolidene catalyst. Rotaxane (*R*)-**6** in which the chiral group is part of the axle moiety also demonstrates that, as in the case of rotaxane (*R*,*R*)-**1** and catenane (*S*,*S*)-**3**, the mechanical bond can be used to enhance enantioselectivity; the corresponding chiral non-interlocked axle mediates the benzoin reaction in just 3% ee whereas rotaxane (*R*)-**6** produces the product in 24% ee.

**Fig. 5:**
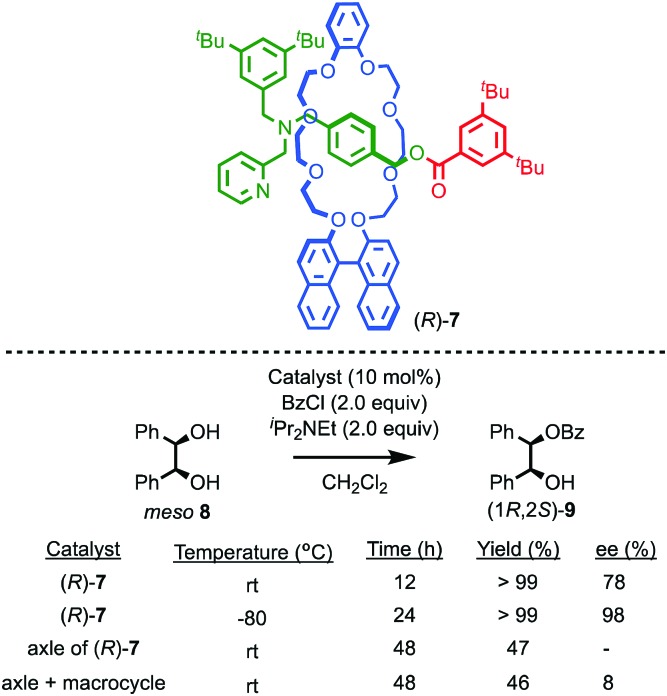
Takata's pyridine-rotaxane (*R*)-**7** for enantioselective acyl transfer catalysis.^20^

Takata and co-workers have extended this approach to acyl transfer catalysis using rotaxane (*R*)-**7** for the desymmetrisation of *meso* diol **8**.^20^ In the presence of rotaxane (*R*)-**7**, (1*R*,2*S*)-**9** was produced in 78% ee with quantitative conversion in 12 h (Fig. 5). Under the same conditions, the corresponding non-interlocked pyridine axle produced just 47% conversion to a racemic product in 48 h. A combination of non-interlocked macrocycle and axle led to similar conversion (46%) and low enantioselectivity (8% ee). Lowering the temperature to –80 °C with rotaxane (*R*)-**7** gave (1*R*,2*S*)-**9** in an impressive 98% ee and quantitative conversion over 24 h. Not only does rotaxane (*R*)-**7** demonstrate the potential for the transfer of chiral information through the mechanical bond to influence reactions that are otherwise hard to render enantioselective, it also shows that the mechanical bond can lead to enhanced catalytic activity, in contrast to the examples of Leigh and Niemeyer presented above, although the reasons for this are unclear.

**Fig. 6:**
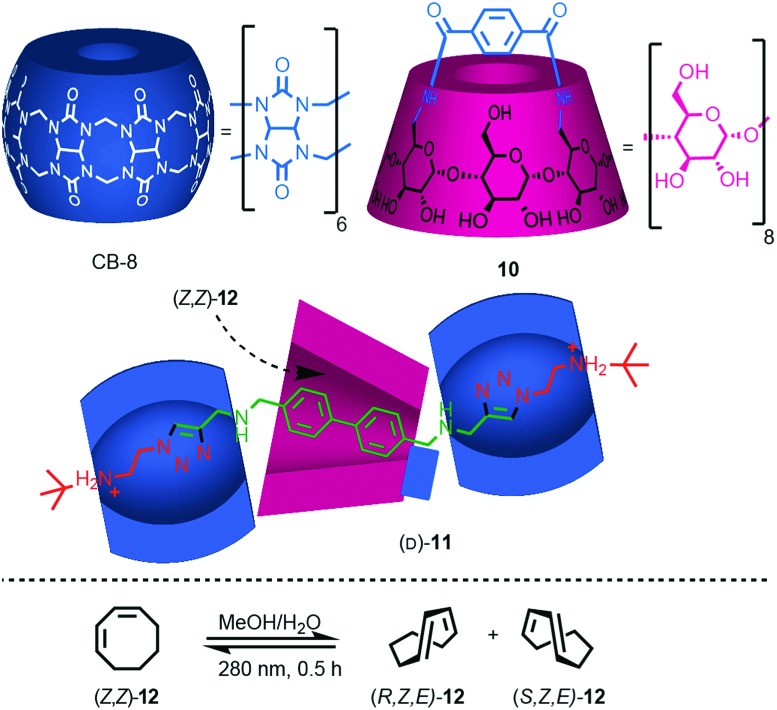
Photochirogenesis with chiral rotaxane (d)-**10**.^21^ Counter ions omitted for clarity.

Yang and co-workers recently demonstrated the potential for the mechanical bond to provide a chiral reaction field for asymmetric photocatalysis.^21^ Rotaxane (d)-**11** (d stereochemical label refers to all six glucose units), which contains a photosensitising group in the axle, was assembled efficiently using Stoddart's cooperative-capture^22^ approach from cucurbit[6]uril (CB6) and macrocycle (d)-**10**, which is derived from γ-(d)-cyclodextrin (γ-[d]-CD) (Fig. 6). Titrations revealed that both non-interlocked macrocycle (d)-**10** and rotaxane (d)-**11** bind (*Z*,*Z*)-1,3-cyclooctadiene ([*Z*,*Z*]-**12**) in a 1 : 1 manner with binding constants *K*_a_ = 2130 and 3820 M^–1^ respectively. The higher binding constant of rotaxane (d)-**11** was attributed to the better fit between the guest and the partially filled cavity of the γ-(d)-CD macrocycle compared with the relatively flexible cavity of (d)-**10** alone. Photoisomerisation of (*Z*,*Z*)-**12** using light at 280 nm in water in the presence of either macrocycle (d)-**10** or (d)-**11** produced very different levels of enantioselectivity: (*Z*,*E*)-**12** was produced in just 1.1% ee in the case of (d)-**10** whereas rotaxane (d)-**11** gave rise to 12.8% ee under the same conditions. The higher enantioselectivity was attributed to the tighter fit between the substrate and the chiral macrocycle in rotaxane (d)-**11** which better imposes a chiral reaction field.

**Fig. 7:**
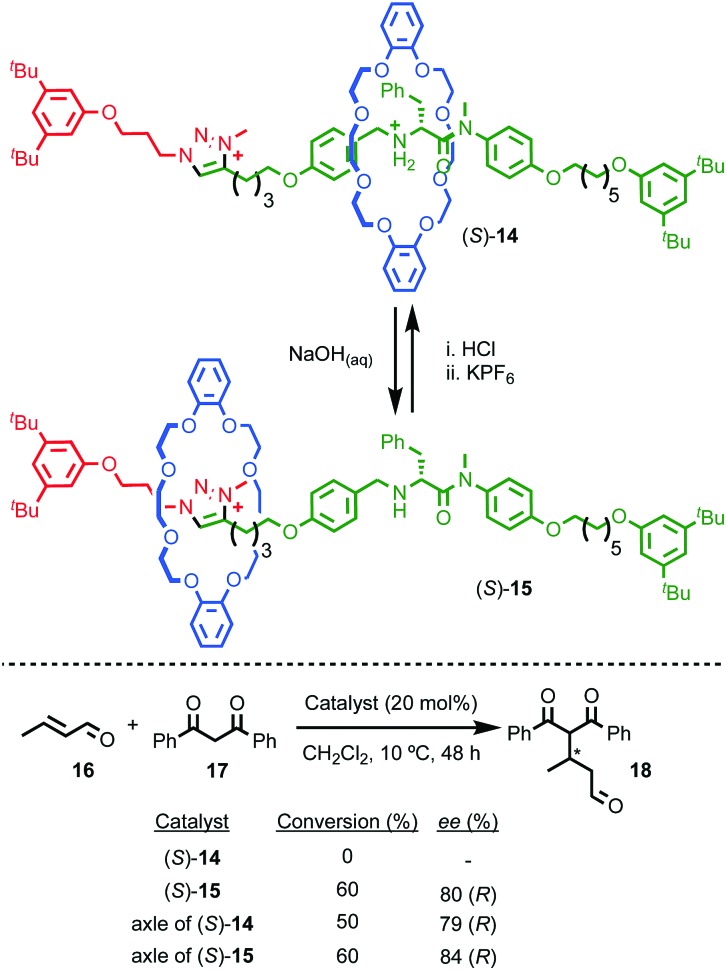
Leigh's switchable chiral rotaxane catalyst.^26^ Counter ions omitted for clarity.

Perhaps the best-known application of interlocked molecules is as components of molecular machines,10b–g which culminated in 2016 in the award of the Nobel prize for Chemistry to Stoddart and Sauvage, alongside Feringa.^23^ Leigh and co-workers have demonstrated the potential of combining catalytic functionality with the well-developed chemistry of molecular shuttles^24^ to generate “switchable” catalysts.^25^ In 2014 they extended this approach to an enantioselective variant.^26^ Rotaxane (*S*)-**14** (Fig. 7) contains a chiral stereogenic secondary amine moiety that can catalyse a wide variety of organocatalytic transformations.^27^ However, protonated rotaxane (*S*)-**14** is catalytically inactive as the macrocycle binds to the ammonium unit, shielding it sterically and also acting to raise its p*K*_a_,^28^ both of which can be expected to inhibit its catalytic activity. Deprotonation to give rotaxane (*S*)-**15** causes the macrocycle to shuttle to the triazolium station, revealing the catalytically competent amine function which can mediate the Michael addition of diketone **17** to crotonaldehyde (**16**) to produce **18** in up to 80% ee, which is comparable with the non-interlocked axle (84% ee).

Despite relatively few examples thus far, these results demonstrate that chiral interlocked molecules assembled using well-developed templating approaches with simple covalent stereogenic building blocks can deliver catalysts which enhance enantioselectivity compared to their non-interlocked counterparts. In particular, Takata's demonstration of the use of the mechanical bond to impose a chirotopic environment on an otherwise achiral subcomponent containing the catalytic functionality is a strategy that may allow catalytic modalities that are otherwise hard to render enantioselective to be readily modified by employing the reaction field created by the mechanical bond.

### MIMs containing covalent stereogenic units as hosts and sensors

**Fig. 8:**
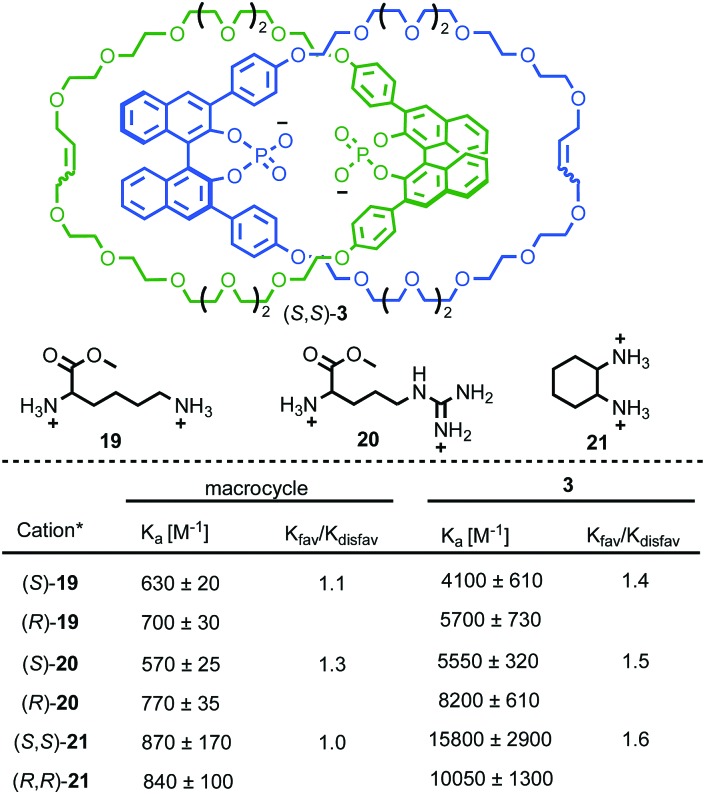
Niemeyer's chiral phosphate catenane for selective chiral cation binding.^29^ All cations were employed as their bis-HCl salt. Counter ions omitted for clarity.

As demonstrated in the previous section, the combination of chirality and mechanical bonding can create a well-expressed chirotopic reaction field. In addition to catalysis, this can be exploited for the related binding of chiral analytes. Indeed, Niemeyer and co-workers’ organocatalytic catenane (*S*,*S*)-**3** was originally reported as its conjugate base as an anionic chiral receptor for doubly charged amino acid derivatives. Treatment of enantiopure (*S*,*S*)-**3** as its tetrabutylammonium (TBA) salt with arginine, lysine or *trans*-diaminocyclohexane bis-HCl salts led to significant changes in the ^1^H NMR spectrum of the host, indicative of complex formation (Fig. 8).^29^ The enantiomers of the guests induced subtly different changes in the host ^1^H NMR and the association constant of these diastereomeric host–guest complexes were determined to be different from one another, indicating diastereoselective binding. Catenane (*S*,*S*)-**3** exhibits both higher binding constants with the doubly cationic guests and higher diastereoselectivity compared with the corresponding non-interlocked macrocycle. The higher selectivity of catenane (*S*,*S*)-**3** can be tentatively attributed to the smaller, more sterically hindered nature of the cavity formed between the two rings compared with the macrocycle alone.

**Fig. 9:**
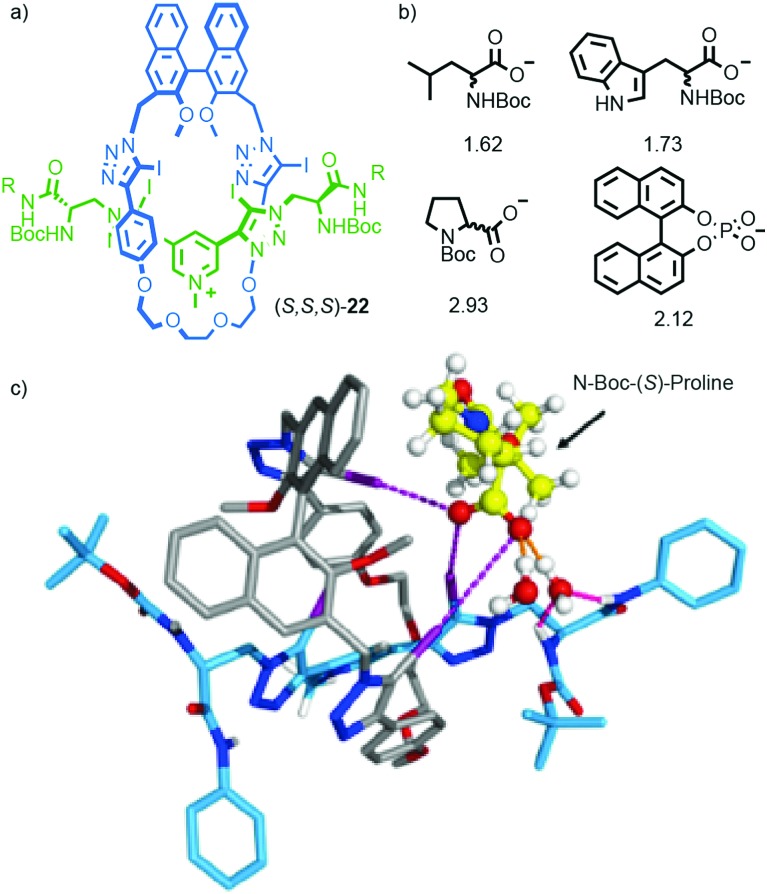
(a) Beer's chiral rotaxane (*S*)-**22** for selective chiral anion binding.^31^ (b) Representative anionic guests (used as TBA salts) and the selectivity observed for the preferred enantiomer in each case. (c) Modelled structure of (*S*)-**22** with *N*-Boc-(*S*)-proline. R = 4-(C_6_H_4_)-C(4-(C_6_H_4_)-^*t*^Bu)_3_ Reprinted with permission from ref. 31. Copyright 2017 American Chemical Society. Counter ions omitted for clarity.

More recently, Beer and co-workers extended their work on the binding of anions in interlocked molecules^30^ to chiral analytes.^31^ Rotaxane host (*S*)-**22** was synthesised using Beer's iodo-acetylene modification^32^ of the active template^33^ Cu-mediated alkyne-azide cycloaddition^34^ (AT-CuAAC)^35^ reaction. Rotaxane (*S*)-**22** interacts with enantiomeric amino acid carboxylate salts to produce complexes with different binding constants through a combination of H-bonding, charge–charge interactions and halogen bonding (Fig. 9). Anion binding enantioselectivities of up to ∼3 : 1 could be achieved in the case of proline and reasonable selectivities (>1.5) were observed for other analytes. Replacing the chiral macrocycle with an achiral analogue led to a dramatic reduction in selectivity. Replacing the chiral axle with an achiral unit that lacked the H-bond donors altered the selectivities observed for each analyte, but the host remained highly selective. The anion binding enantioselectivities of the non-interlocked macrocycle or axle were lower than those observed for (*S*)-**22**. Computer modelling (Fig. 9c) suggested that the high selectivity observed in the case of (*S*)-**22** is due to multi-point interactions between the host and guest.^36^

The development of hosts capable of discriminating between enantiomers of a given analyte and reporting the binding event optically or electrochemically is a current challenge in analytical chemistry for the development of high throughput methods for screening reaction enantioselectivity.^37^ Although this remains in its infancy, the results from Niemeyer and Beer suggest that chiral interlocked molecules have a role to play in answering this challenge, particularly given Beer's previous successes in applying this approach to selective anion binding.^30^

### Chiroptical properties of MIMs containing covalent stereogenic units

One of the properties of chiral molecules that intrigued scientists in the 19th century,^2^ and the one that students of chemistry are first introduced to, is optical rotation. Unsurprisingly, chiral MIMs containing covalent stereogenic elements display optical rotation and related chiroptical properties. However, in some cases, the mechanical bond clearly augments these properties, producing molecules with unusual chiroptical properties.

**Fig. 10:**
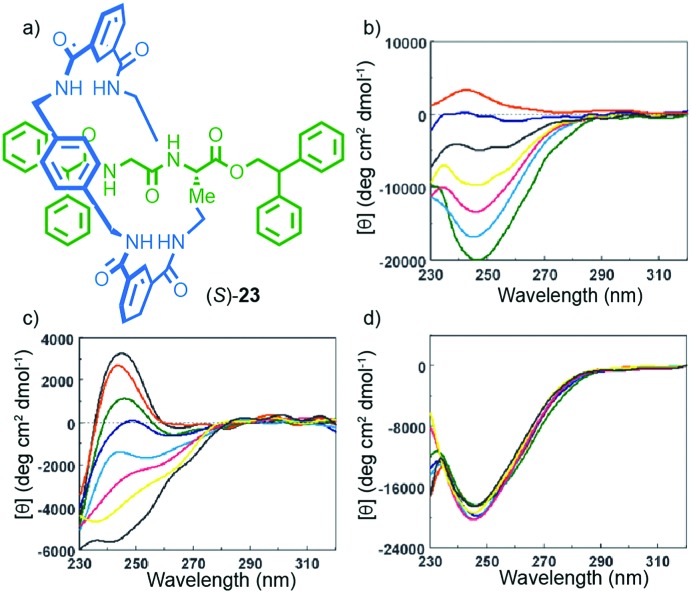
(a) Structure of Leigh's peptidic rotaxane (*S*)-**23**.^39^ (b) Solvent-dependent CD spectra (0.1 mM) of rotaxane **23** in CHCl_3_ (green), 1 : 1 CHCl_3_/MeOH (cyan), 2 : 3 CHCl_3_/MeOH (yellow), 1 : 5 CHCl_3_/MeOH (black), 1 : 10 CHCl_3_/MeOH (blue) and 100% MeOH (red). Temperature-dependent CD spectra (0.1 mM) of rotaxane (*S*)-**23** in (c) MeOH and (d) CHCl_3_ :  263 K (black), 273 K (red), 283 K (green), 293 K (blue), 303 K (cyan), 313 K (magenta), 323 K (yellow) and 333 K (black) Reprinted with permission from ref. 39. Copyright 2002 American Chemical Society.

Leigh and co-workers provided a clear example of how the mechanical bond can contribute to the transfer of chiral information within the covalent components of a mechanically bonded structure. Rotaxane (*S*)-**23** was synthesised using a five-component clipping reaction,^38^ and its circular dichroism (CD) spectra measured under various conditions (Fig. 10).^39^ The interlocked product displayed significantly stronger CD signals than the non-interlocked axle by approximately an order of magnitude. Furthermore, changes in solvent led to significant variations in the CD response of rotaxane (*S*)-**23**; a larger molar ellipticity was observed in less polar, non-H-bonding solvents and, strikingly, the sign of the observed CD signal was inverted in CHCl_3_*vs.* MeOH (Fig. 10b). Variation of temperature also had different effects on the observed CD depending on the solvent with much larger spectra changes observed in MeOH than in CHCl_3_ (Fig. 10c and d). A combination of X-ray crystallography and computational analysis revealed that the C-terminal diphenylmethine stopper is the major contributor to the observed chiroptical response. In this case, the role of the crowded mechanical bond appears to be the transfer the stereochemical information contained in the peptide moiety to the stopper moiety *via* the achiral macrocycle, presumably by enforcing a preferred chiral conformation in the latter. The effect of solvent can then be rationalised by considering that competitive solvents will interrupt the inter-component hydrogen bonds and thus reduce the organisation of the structure.

**Fig. 11:**
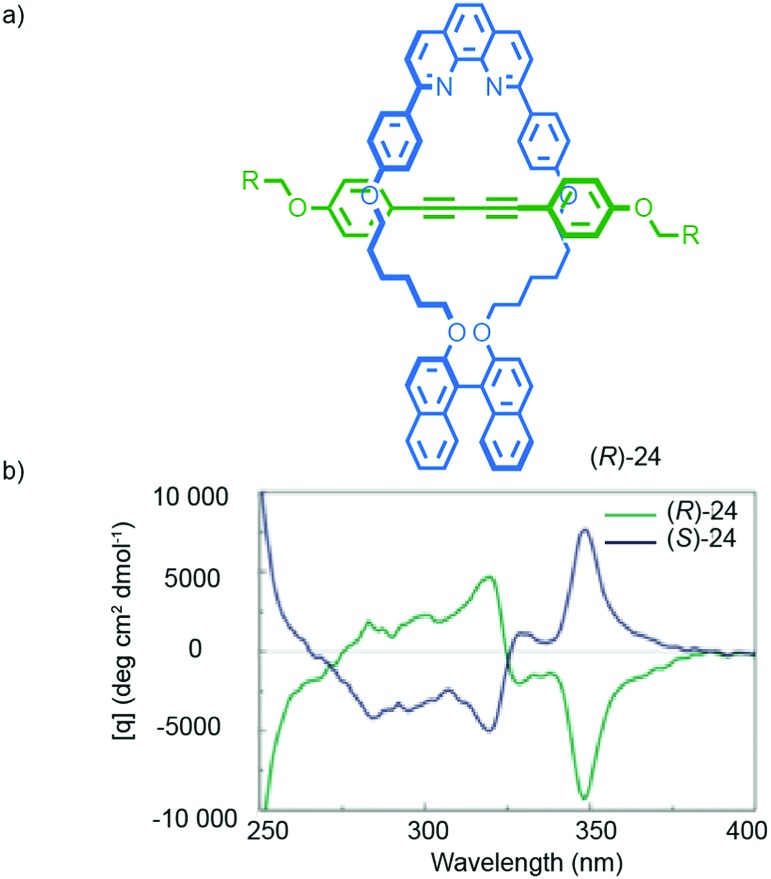
Transfer of chiral information between the macrocycle and axle in Saito's axially chiral rotaxane (*R*)-**24**.^40^ R = C(4-(C_6_H_4_)-(4-(C_6_H_4_)-cyclohexyl))_3_. Reprinted with permission from ref. 40. Copyright 2015 The Chemical Society of Japan.

Chiral information in one component can also be transferred through the mechanical bond, as in the case of Takata's catalysts **5–7**, to enforce a chiral environment onto and thus induce a chiroptical response from an achiral component. For instance, Saito and co-workers reported rotaxane (*R*)-**24** in which an achiral diyne axle is combined with a stereogenic binaphthyl-containing macrocycle (Fig. 11).^40^ The CD spectrum of rotaxane (*R*)-**24** contains bands at 320 and 348 nm that are attributed to the achiral axle that is held in the chirotopic environment of the macrocycle by the mechanical bond. Chen and co-workers proposed similar effects in the case of an unusual triply-threaded [2]catenane.^41^

**Fig. 12:**
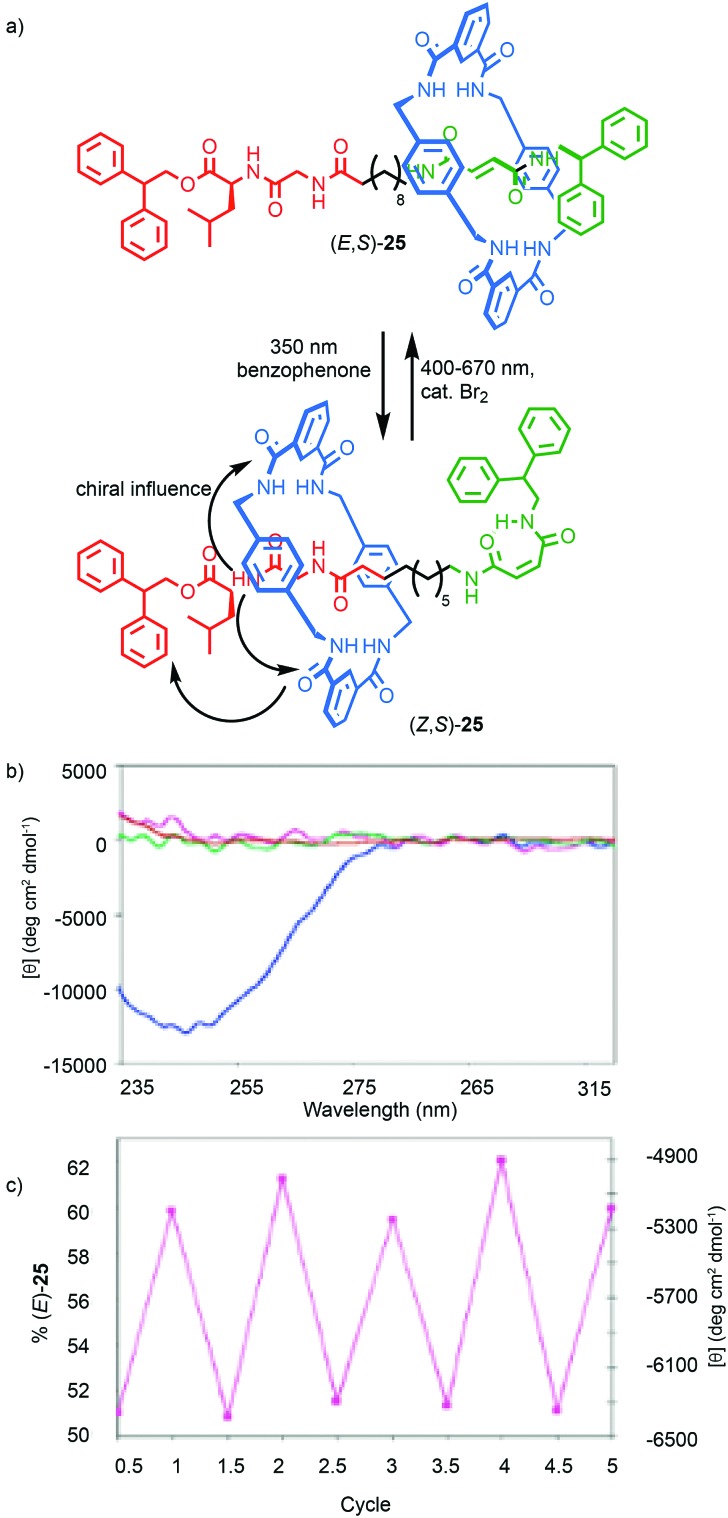
(a) Leigh's peptidic rotaxane (*Z/E*,*S*)-**25**.^42^ (b) CD spectra (0.1 mM in CHCl_3_) at 298 K of (*Z*,*S*)-**25** (blue), (*E*,*S*)-**25** (purple), and the corresponding axles (red and green respectively). (c) Variation in CD response with irradiation after alternating radiation at 254 nm (half integers) and at 312 (integers) for five complete cycles (percentage of (*E*,*S*)-**25** at the photostationary state on left-hand *Y* axis, right-hand *Y* axis shows the CD absorption at 246 nm). Reprinted with permission from ref. 42. Copyright 2003 American Chemical Society.

The transfer of chiral information within chiral MIMs has been extended to molecular shuttles to generate examples that exhibit switchable chiroptical properties. Leigh and co-workers synthesised shuttle (*E*,*S*)-**25** that contains a fumaramide and a chiral Gly–Val binding site for the macrocycle (Fig. 12).^42^ The macrocycle predominantly occupies the fumaramide “station” preferentially, placing it far from the stereogenic unit and in this state the system shows negligible CD signal, as does the non-interlocked axle. Irradiation of (*E*,*S*)-**25** in the presence of a photosensitizer isomerises the fumaramide station to the maleamide geometric isomer (*Z*,*S*)-**25** (∼70% photostationary state). In (*Z*,*S*)-**25**, the macrocycle preferentially occupies the Gly–Val station, placing the macrocycle in a well expressed chirotopic environment, resulting in a large increase in the CD signal. Back isomerisation in the presence of Br_2_ returns the system to its initial state. Alternatively, switching between 49% (*Z*,*S*)-**25**:(*E*,*S*)-**25** and 38% (*Z*,*S*)-**25**:(*E*,*S*)-**25** can be achieved by irradiation at 254 nm and reversed at 312 nm, allowing the authors to demonstrate the photochemical switching of the CD signal.

**Fig. 13:**
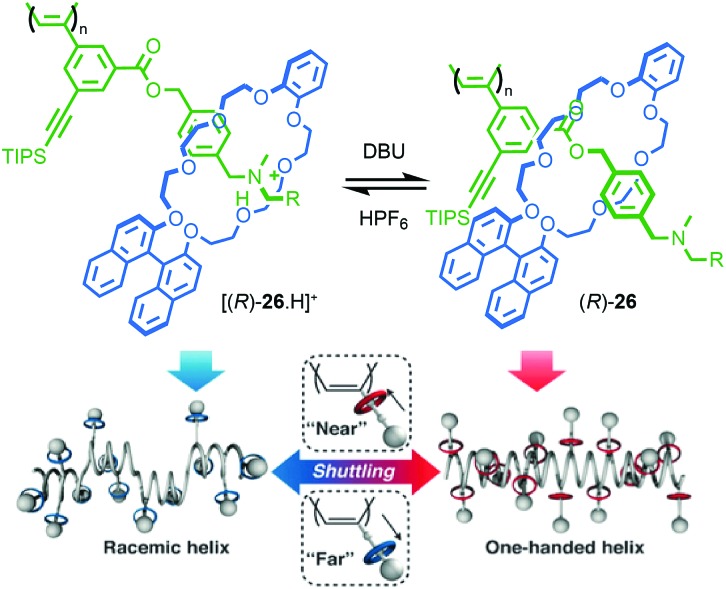
Takata's helical polymer switch rotaxane (*R*)-**26** (R = 3,5-^*t*^Bu-C_6_H_3_).^43^ Counter ions omitted for clarity. Reproduced from ref. 43 with permission from The Royal Society for Chemistry.

Takata and co-workers extended this approach to polymeric materials by using a molecular shuttle to control the helical twisting of a polyacetylene macromolecule (Fig. 13).^43^,^44^ Side chain polyrotaxane (*R*)-**26** (“*R*” refers to the stereodescriptor of all of the macrocycles in the side chains) was produced by polymerisation of a chiral [2]rotaxane monomer. When the axle is protonated, the macrocycle bearing the stereogenic binaphthyl unit occupies the ammonium station far from the polymer backbone due to H-bonding interactions and the chain adopts a random, racemic helical conformation. As a result, no significant CD signal is observed attributable to the polymer chain. Deprotonation of the ammonium unit results in the net displacement of the macrocycle towards the polymer chain. This leads to an increased influence of the stereogenic unit in the macrocycle on the conformation of the polymer backbone and the appearance of a CD signal associated with the polyacetylene moiety as it adopts a predominantly single handed helical conformation.

The chiroptical properties of chiral interlocked molecules, which are in many cases distinct from those of the constituent subcomponents, are readily studied and provide not only an insight into their structures but also the opportunity to generate systems with switchable optical properties for materials applications.

### Chirality as a consideration in the synthesis of MIMs

Having discussed some of the applications of MIMs containing chirotopic stereogenic units, it is worth considering how including chiral subcomponents affects their synthesis. The most obvious consequence of including elements of covalent chirality in MIM subcomponents is that of increased complexity. This manifests itself particularly in the analysis of the products; interlocked molecules typically exhibit complex NMR spectra and this is further complicated when stereogenic units are introduced. This is one reason why, where possible, stereogenic elements are excluded/removed from interlocked molecules. For example, Chiu and co-workers hydrogenate the product of their “slipping-followed-by-swelling” approach to remove a stereogenic centre and simplify the interpretation of its ^1^H NMR data.^45^

**Fig. 14:**
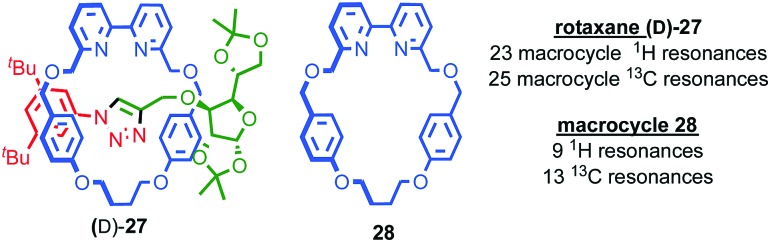
Goldup's chiral, crowded sugar-based rotaxane (d)-**27** and a comparison of the number of resonances attributable to its macrocycle component and those of macrocycle **28** alone.^46^

Conversely, the appearance of complexity in the NMR data of a molecule can, as with the chiroptical properties discussed above, provide an indication of effective transfer of chiral information between the covalent sub-components; Goldup and co-workers observed significant desymmetrisation of the macrocycle component of rotaxane (d)-**27** (the d stereodescriptor defines all of the stereocentres in the stopper unit, which is derived from d-glucose) by both ^1^H and ^13^C NMR, which was attributed to the small, crowded nature of the interlocked product (Fig. 14),^46^ an observation that ultimately led to the development of a new approach to chiral rotaxanes (see below).

**Fig. 15:**
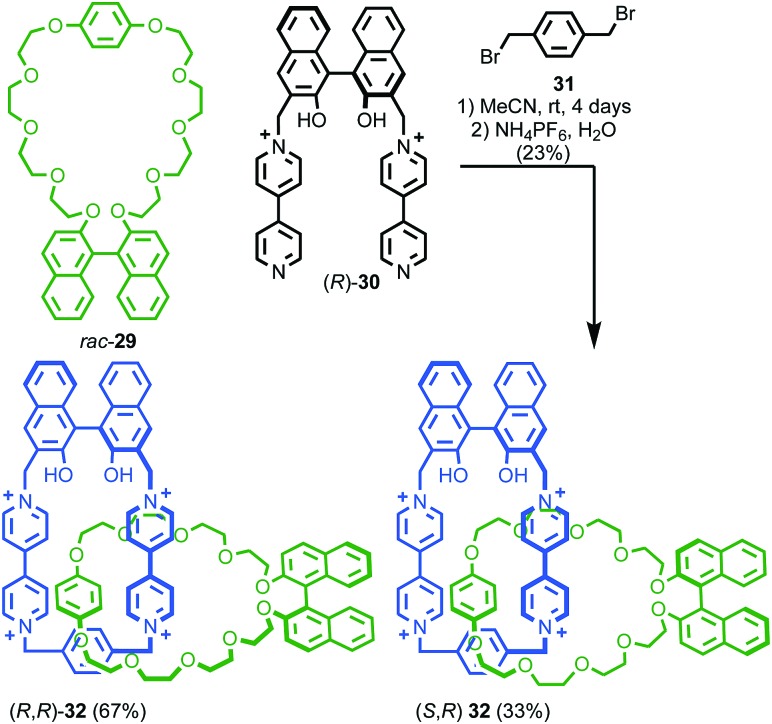
Stoddart's diastereoselective synthesis of catenane **32** under kinetic control.^47^ Counter ions omitted for clarity.

When two or more of the covalent subcomponents contain covalent stereogenic elements the situation becomes more complex as there is the potential for the formation of mixtures of diastereomers. Stoddart and co-workers observed diastereoselectivity in the formation of catenane **32** when enantiopure macrocycle precursor (*R*)-**30** was combined with racemic macrocycle **29**.^47^ Ring closure of diastereomeric intermediates from the reaction occurred with different rates, resulting in an ∼2 : 1 mixture of diastereomers (*R*,*R*)-**32** and (*R*,*S*)-**32** (Fig. 15).

**Fig. 16:**
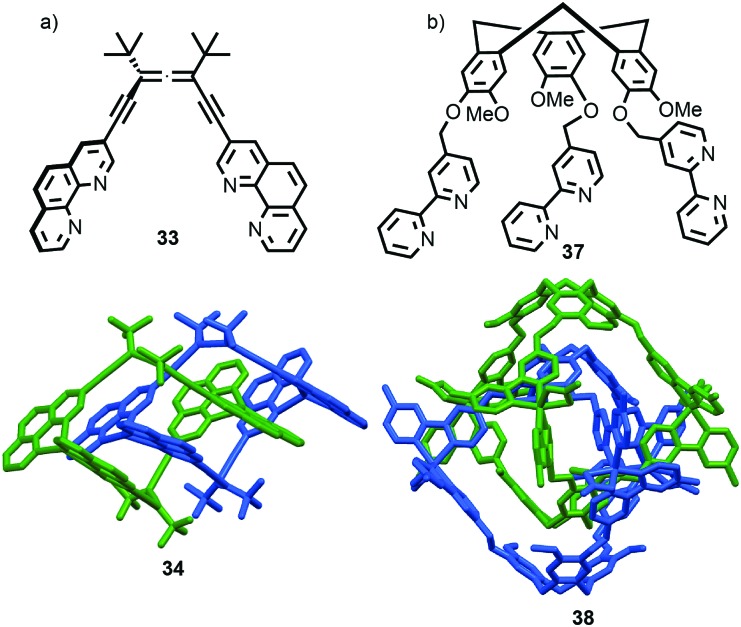
(a) Diederich's self-sorting synthesis of Ag-coordinated catenanes **34** from racemic **33**;^50^ (b) Hardie's triply interlocked catenane **38** synthesised with control over all six metal stereocentres. Counter ions omitted for clarity.^52^

The example from Stoddart and co-workers takes place under kinetic control.^48^ Higher levels of diastereocontrol have been achieved through the self-sorting of chiral subcomponents under thermodynamic control. Diederich and co-workers found that when a racemic mixture of chiral allene ligand **33** was mixed with Ag^I^ ions, a racemic mixture of homochiral catenanes **34** was produced which are composed of homochiral helicates^49^ in which the at-metal absolute stereochemistry is also controlled (Fig. 16a).^50^,^51^ An even more complex system was described by Hardie and co-workers. Ligand **37** assembles under thermodynamic control in the presence of Zn^II^ ions to give triply interlocked [2]catenane **38** composed of two interlocked macrocycles derived from two equivalents of **37**. Although each ligand **37** is locked in a chiral conformation in catenane **38**, each macrocycle contains one equivalent each of the *R* and *S* enantiomers and so the organic component, considered in isolation, is overall *meso*. However, the coordination geometry of the Zn^II^ ions which are bound to two bipyridine units and a η^2^-nitrate anion, results in at-metal stereochemistry and all of these stereocentres have the same absolute stereochemistry. Thus, the structure is chiral and both enantiomers were observed in the solid state (Fig. 16b).^52^

**Fig. 17:**
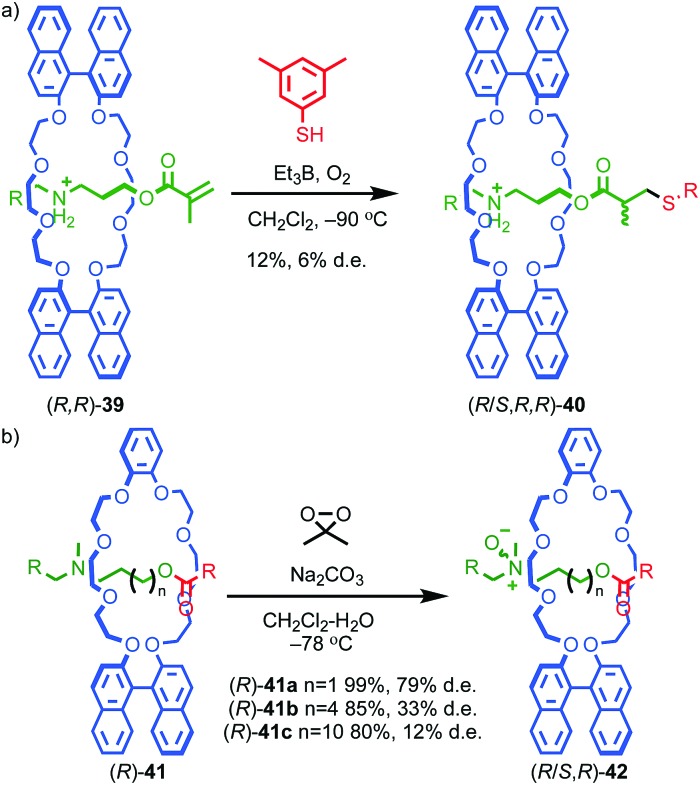
Takata's diastereoselective functionalisation of (a) rotaxane (*R*)-**39** (counter ion omitted for clarity)^53^ and (b) rotaxane (*R*)-**41**.^54^ (R = 3,5-^*t*^Bu-C_6_H_3_).

Takata and co-workers demonstrated diastereoselectivity in the post-synthetic functionalisation of rotaxanes by using the stereogenic element contained in one component to influence the outcome of a reaction affecting the other. In the first example reported, the authors noted a low (6% de) selectivity in the formation of rotaxane (*R*/*S*,*R*,*R*)-**40** assembled from pseudorotaxane (*R*,*R*)-**39** through a stoppering reaction in which the last step is a diastereoselective H-atom abstraction (Fig. 17a).^53^ More recently, Takata and co-workers reported chiral rotaxanes (*R*)-**41** and observed diastereoselective *N*-oxidation when rotaxanes (*R*)-**41** were treated with dimethyldioxirane (Fig. 17b).^54^ The degree of diastereoselectivity in the formation of *N*-oxides (*R*/S,*R*)-**42** varied significantly with the length of the axle, suggesting that the through-space transfer of chiral information between the components depends on the crowded nature of the mechanical bond. Similarly, the effect of varying the substituent of the prochiral nitrogen atom suggested that specific interactions between the chiral macrocycle and prochiral axle play a role in ensuring high diastereoselectivity.

**Fig. 18:**
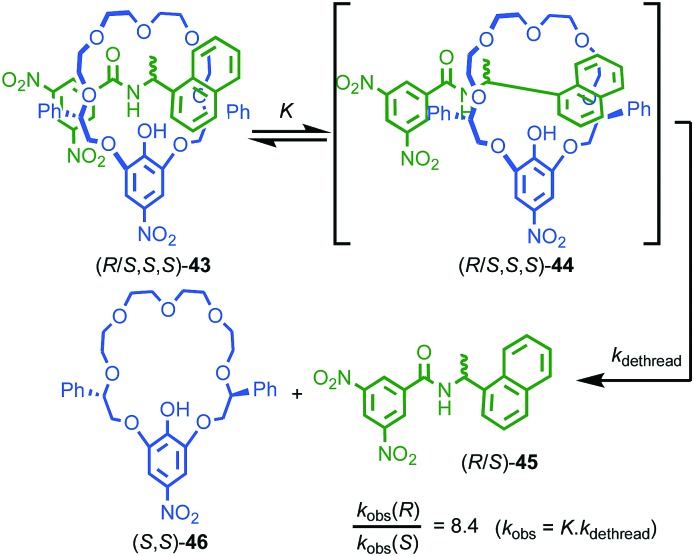
Hirose's observation of the kinetic effect of diastereoisomerism on dethreading rates of pseudorotaxane (*R*/*S*,*S*,*S*)-**43**.^55^

One of the more esoteric effects of chirality on the properties of threaded molecules concerns the dethreading of meta-stable pseudo rotaxanes (*R*/*S*,*S*,*S*)-**43** (Fig. 18).^55^ Hirose and co-workers observed that diastereomeric pseudorotaxanes (*R*/*S*,*S*,*S*)-**43**, which differ in the configuration of a single stereogenic centre, exhibited significantly different rates of decomposition to give their non-interlocked components. Although it is expected that diastereomers display different chemical properties, the difference in the rates of dethreading are quite striking; in toluene *k*_R_/*k*_S_ can reach 8.4, almost an order of magnitude difference! The authors proposed that the mechanism of dethreading involves a pre-equilibrium *via* an activated complex (*R*/*S*,*S*,*S*)-**44** which is significantly biased by the stereochemistry of the axle component, in turn leading to large differences in reaction rate of the dethreading process.

The symmetry properties of interlocked molecules can lead to chirality that is solely the result of the mechanical bond and these forms of molecular asymmetry will be discussed in the next section. However, just as steric hindrance due to covalent substituents can lead the appearance of atropisomerism and thus long lived (*i.e.* observable or even separable) axial stereogenicity, the crowded nature of the mechanical bond can restrict conformational motion in the subcomponents and thus lead to the appearance of chirality even when they are, on average, achiral in their non-interlocked state.

**Fig. 19:**
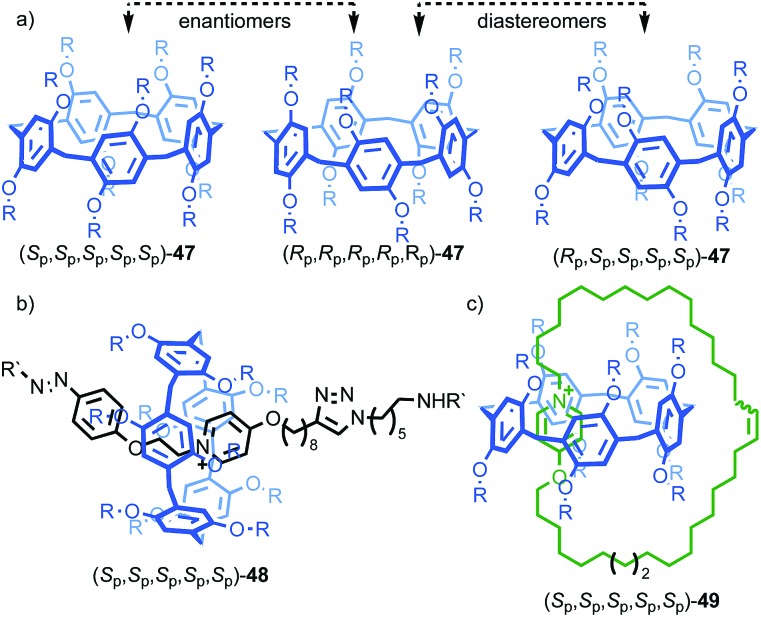
(a) Conformational stereoisomers of pillar[5]arene **47**.^56^ (b) Rotaxane **48** and (c) catenane **49** synthesised from pillar[5]arene **47** as single diastereomers (all-*S*_p_ stereoisomer shown for illustrative purposes).^58^ R = CH_2_CH_3_; R′ = 3,5-^*t*^Bu-C_6_H_3_. Counter ions omitted for clarity.

One of the clearest examples of this phenomenon appears in Ogoshi's pillar[5]arenes, which can exist as a large collection of diastereomers and enantiomers as each of the aromatic rings acts as a planar stereogenic unit, the absolute stereochemistry of which is inverted by rotation of the aromatic ring relative to the macrocycle framework. When the phenoxy substituent is small, in solution these stereoisomers are often in rapid equilibrium and the molecule thus behaves as a single achiral stereoisomer (Fig. 19a).^56^ The formation of interlocked molecules based on pillar[5]arene derivatives results in loss of conformational freedom; the threaded component increases the steric hindrance in the macrocycle cavity preventing the rotation of the aromatic rings to exchange stereoisomers and thus pillar[5]arene rotaxanes and catenanes can exist as separable diastereomers and enantiomers.^57^

Ogoshi and co-workers demonstrated this principle in the high yielding synthesis of [2]rotaxanes and [2]catenanes based on per-ethyl pillar[5]arene (**47**) using a pyridinium template (Fig. 19b).^58^ Strikingly, rotaxane **48** and catenane **49** were formed as a racemic mixture of a single diastereomer that was determined by ^1^H NMR in both cases to be the highly symmetric (*R*_p_,*R*_p_,*R*_p_,*R*_p_,*R*_p_)* isomer.^59^ These could be separated by chiral stationary phase HPLC, demonstrating their configurational stability. The high diastereoselectivity observed was rationalised by the higher stability of the inclusion complex of the pyridinium moiety and the axle precursor in the highly symmetrical (*R*_p_,*R*_p_,*R*_p_,*R*_p_,*R*_p_) conformation. Thus, a single conformation of the host guest complex predominates prior to covalent bond formation to capture the interlocked molecule. The authors also reported the corresponding [3]rotaxane which was formed as a statistical mixture of racemic chiral and *meso* diastereomers.^60^

**Fig. 20:**
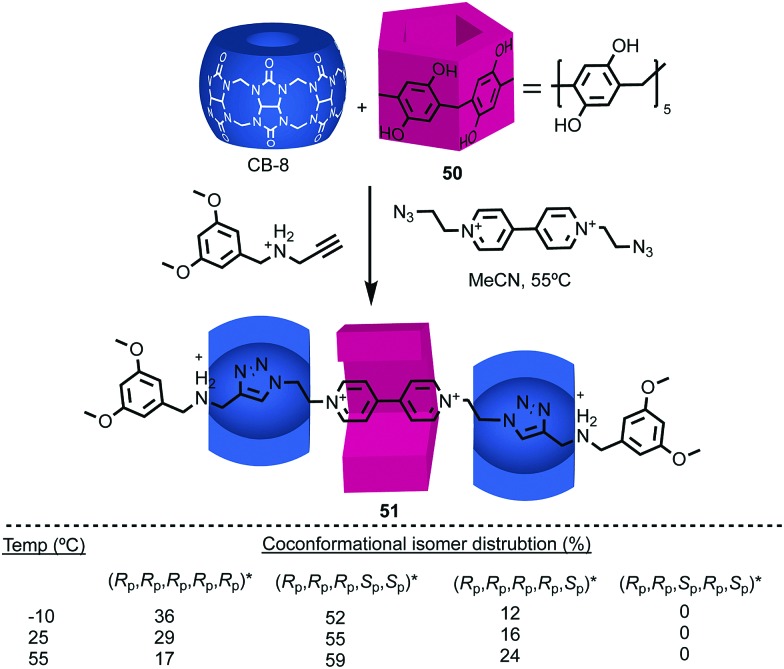
[4]Rotaxane **51** synthesised as a mixture of diastereomers^59^ using Stoddart's cooperative capture approach.^62^ Counter ions omitted for clarity.

Stoddart and co-workers applied pillar[5]arene **50** in their cooperative capture approach to produce hetero[4]rotaxane **51** (Fig. 20).^61^ As in **48**, the threaded axle removes the conformational freedom of the pillararene macrocycle. However, in this case the product is formed as a mixture of diastereomers, the composition of which varies with temperature. The lower diastereoselectivity observed in the case of **51** compared with **48** reflects the significant difference in the mechanism of formation. In the case of **48**, the observed diastereoselectivity reflects the greater thermodynamic stability of the (*R*_p_,*R*_p_,*R*_p_,*R*_p_,*R*_p_)* 59 host guest complex. In contrast, the formation of **51** takes place under kinetic control with each of the pillararene diastereomers forming a different activated complex with the cucurbituril macrocycles with its own stability compared with the starting materials (*i.e.* different pre-equilibria) and rates of progress towards the product.

**Fig. 21:**
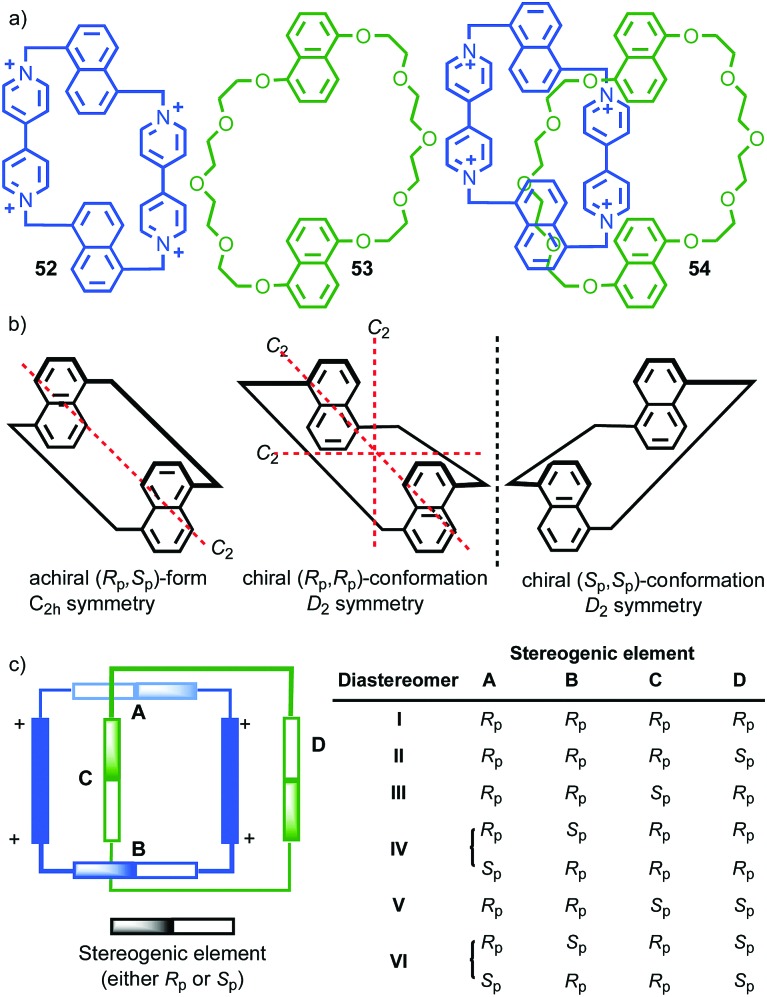
Stoddart's stereochemical Rubik's cube **54** that exists as a complex mixture of diastereomers in slow exchange as a result of the mechanical bond.^63^ (a) Structures of parent macrocycles **52**, **53** and catenane **54**. (b) Conformational stereoisomers of macrocycles **52** and **53**. (c) Stereoisomerism in catenane **54** (enantiomers of each diastereomer not shown). Counter ions omitted for clarity.

Interlocked molecules derived from pillararenes form stereoisomers that are essentially locked as a single atropisomer. Similar but dynamic conformational isomerism can arise in interlocked molecules and, by dint of the crowded nature of the mechanical bond, when multiple such units are present, well-expressed diastereoisomerism can result that can be observed by ^1^H NMR. A striking example of this effect was provided by Stoddart and co-workers in their detailed analysis of catenane **54** (Fig. 21).^62^ Both macrocycles in **54** contain moieties that act as conformational chiral planar stereogenic units; the naphthyl units in both parent macrocycles **52** and **53** can adopt either an *R*_p_ or *S*_p_ conformation (Fig. 21b). In the case of **54**, this results in diastereomers that are in slow exchange on the ^1^H NMR timescale. Although catenane **54** contains four stereogenic units, suggesting that a maximum of 16 stereoisomers are possible, examination of models in which A and B have opposite configuration reveals that the *R*_p_, *S*_p_ and *S*_p_, *R*_p_ configurations are equivalent by permutation of units A and B.^63^ Thus, catenane **52** can be shown to have 6 possible diastereomers (Fig. 21c) and their corresponding enantiomers, 12 stereoisomers in total, making **54** a veritable stereochemical Rubik's™ cube!

The stereoisomers of **54** can exchange *via* a number of pathways; (i) rotation about the single bonds of each naphthyl unit inverts each stereogenic unit and so all diastereomers and enantiomers can be interchanged by stepwise inversion of the individual elements; (ii) pirouetting of the neutral macrocycle through the tetracationic cyclophane exchanges between diastereoisomers (*e.g.* (*R*_p_,*R*_p_,*R*_p_,*S*_p_)-**54** to (*R*_p_,*R*_p_,*S*_p_,*R*_p_)-**54**). However, counter-intuitively, pirouetting of the tetracationic cyclophane through the neutral macrocycle is a degenerate process, always regenerating the original diastereoisomer.

**Fig. 22:**
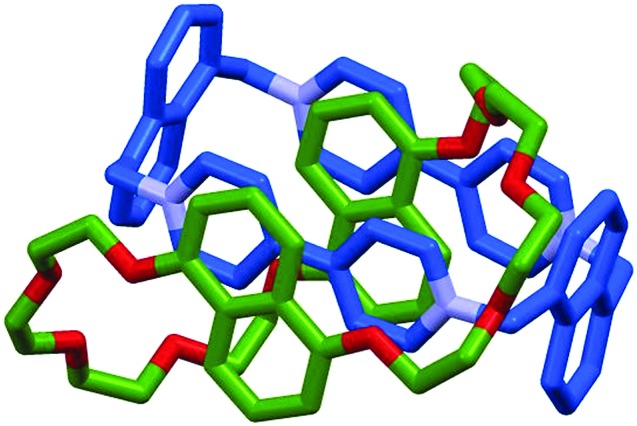
Solid state structure of (*S*_p_*S*_p_*S*_p_*S*_p_)-**54**. Counter ions omitted for clarity.

Variable temperature ^1^H NMR, in combination with the study of model compounds, revealed that although the conformational inversion of the dialkoxy naphthyl unit that lies outside the cavity (**D**) is rapid, all other conformational inversion processes are slow. Thus, unit **D** can be ignored as it is functionally symmetrical on the ^1^H NMR timescale, making diastereomers **I** and **II**, **III** and **V**, and **IV** and **VI** equivalent. In contrast, the other elements are not interconverted on the ^1^H NMR timescale. Similarly, the pirouetting of the neutral macrocycle is slow. Thus, the enantiomers and diastereomers of **54** are essentially static on the ^1^H NMR timescale and multiple resonances are observed for each proton. Key signals of isomers **I**/**II** and **III**/**V** were found to be isochronous while signals corresponding to isomer **IV**/**VI** could be integrated separately resulting in a ratio of (**I**/**II** + **III**/**V**) : **IV**/**VI** of approximately 3 : 1. This ratio could be improved by fractional crystallisation up to 93 : 7, underlining the configurational stability of **54**. Ultimately a solid-state structure of isomer **I** was obtained as a single enantiomer in which all stereogenic units are assigned as *S*_p_ (Fig. 22).

#### Conclusions

The examples presented demonstrate that the crowded environment of the mechanical bond can lead to highly diastereoselective reactions, including in the post synthetic modification of achiral covalent subcomponents. This presents opportunities both for the synthesis of highly ordered chiral structures from simple building blocks and also in the modification of achiral subunits using the mechanical bond to direct the reaction. Furthermore, the kinetic effects noted by Hirose and co-workers suggest that kinetic self-sorting^64^ approaches to chiral interlocked molecules may be possible, although they have yet to be exploited. The effect of the mechanical bond on conformational stereogenic units underlines the potential opportunities, as in the case of the highly diastereoselective synthesis of interlocked pillararenes, but also introduces complications,^65^ as amply demonstrated by Stoddart's cyclophanes, that conformational stereoisomerism can present in mechanically bonded structures.

## Chirotopic mechanical stereogenic elements

The preceding section focusses on how including covalent chiral stereogenic elements can affect the behaviour and applications of interlocked molecules. The mechanical bond itself can lead to chirality that cannot be attributed to a single covalent stereogenic element and such molecules have been described as “mechanically chiral” as the mechanical bond itself, combined with the underlying symmetry properties of the covalent sub-components, can be identified as the stereogenic unit.

This section is organised by the different conditional mechanical stereogenic elements that have been reported in interlocked molecules, focussing on their underlying structural features, aspects of their synthesis in enantiopure form and, where relevant, a discussion of their properties and examples of their applications. We will also outline the accepted method of assigning absolute stereochemistry to these mechanical stereogenic units or, where the situation remains ambiguous or unaddressed, we will propose nomenclature or modifications of existing proposals for the assignment of absolute stereochemistry in these systems.

Before introducing the archetypal conditional mechanical stereogenic elements however, first we must introduce and clarify the concept of topology, a term which is much abused and confused in recent literature. When considering a molecule's topology, the only fixed property of the system is the atomic connectivity (the molecular graph). All other properties (bond angles and lengths) can be varied arbitrarily on the one condition that bonds and atoms should not pass through one another.^10^ When considered on these terms, chiral covalent stereogenic units (*e.g.* centres, axes, helices and planes) are considered topologically non-stereogenic as they can be continuously deformed into their enantiomers. Indeed, all of the molecules discussed so far are only chiral in the Euclidean sense, in that their chirality relies on treating bonds as fixed vectors in three-dimensional space. Interlocked molecules can display Euclidean chirality or topological chirality as a result of the mechanical bond and these various classifications will be clarified in the following sections.

**Fig. 23:**
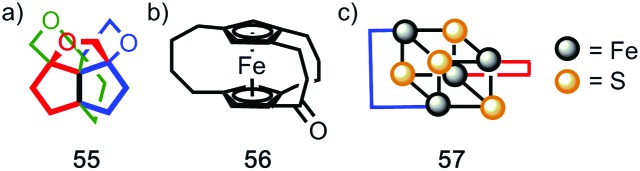
Covalent structures that display topological chirality. (a) Propeller-shaped organic molecule **55**.^67^ (b) Topologically chiral ferrocene **56**.^68^ (c) A schematic representation (**57**) of the iron–sulfur cluster of the Chromutium high potential iron protein.^69^

Finally, it is worth noting that topological chirality is not solely the preserve of interlocked molecules. Covalent molecules, in particular metal complexes, can display topological chirality (Fig. 23).^66^ The other class of molecules that often display topological chirality are the knots, which will be discussed briefly later in this review due to their relationship with interlocked structures.

### The mechanical planar chiral stereogenic unit

#### Description

**Fig. 24:**
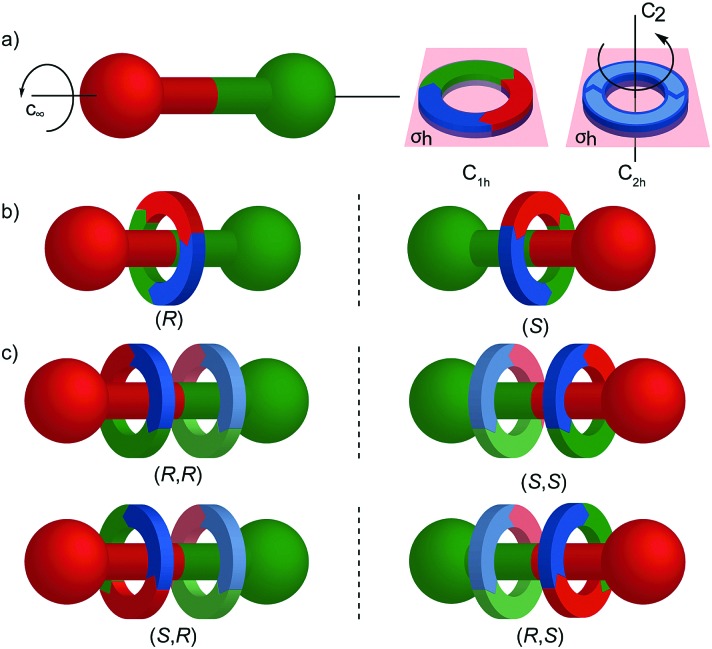
Cartoon representation of mechanically planar chiral rotaxanes and their components. (a) Axle (*C*_∞v_) and macrocycle (*C*_1h_, *C*_2h_) components suitable for inclusion in mechanically planar chiral rotaxanes. (b) A mechanically planar chiral [2]rotaxane. (c) A mechanically planar chiral [3]rotaxane and the stereoisomers possible for a given order of macrocycles on the axle.

As recognised by Schill,^70^ when a *C*_*n*h_ symmetric macrocycle (principle axis perpendicular to the macrocycle plane, mirror plane parallel to the macrocycle plane) encircles a *C*_*n*v_ axle (principle axis and mirror plane(s) aligned with the long axis of the axle) (Fig. 24a), the resultant structure is chiral (Fig. 24b). Macrocycles that have *C*_*n*h_ symmetry are often described as “oriented” because chemically speaking such symmetry is achieved by a sequence of substituents around the ring skeleton. Although *C*_*n*h_ symmetry is present when a repeating oriented unit is used to form the macrocycle, most examples of mechanically planar chiral rotaxanes are composed of *C*_1h_ (more commonly referred to as *C*_s_) symmetry rings, the only symmetry operation of which is a single mirror plane.

The reduction in symmetry on forming the mechanical bond leading to the appearance of chirality, even in simple cartoons devoid of chemical structure, can be understood in terms of how mechanical bond formation decreases the symmetry of the ensemble compared with that of the individual components, and in particular, how it affects their improper symmetry elements. Considering a mechanically planar chiral rotaxane composed of a *C*_1h_ macrocycle and a *C*_∞v_ axle, the improper symmetry operations of the components are a σ_h_ (parallel with the ring plane) and an infinite number of σ_v_ (aligned with the axle) respectively (Fig. 24a). When the macrocycle encircles the axle, the mechanical bond enforces a relative orientation that ensures the σ_h_ and σ_v_ planes cannot coincide in any threaded representation of the rotaxane. Thus, the improper symmetry operations of the components are not symmetry operations of the whole and can be said to be removed on mechanical bond formation. Lacking any improper symmetry operations, the interlocked structure is chiral.

As an aside, given that the enantiomers of a mechanically planar chiral rotaxane cannot be exchanged by continuous deformation of the covalent framework, the stereochemistry of mechanically planar chiral rotaxanes is non-Euclidean, but given that rotaxanes are topologically trivial, also non-topological. They are thus unique in this section in being described simply as mechanically chiral, all other examples being classified as mechanical as well as Euclidean or topological.

Although Schill suggested the term “cyclochiral” to describe this form of molecular asymmetry,^71^ Takata and co-workers later suggested that this form of chirality could be considered analogous to planar chirality in covalent structures,^71^ with the macrocycle playing the role of a plane which is desymmetrised by the axle to yield a chirotopic stereogenic unit. Goldup and co-workers subsequently extended this nomenclature to suggest that such rotaxanes should be considered “mechanically planar chiral” to emphasise the role of the mechanical bond in as the stereogenic element and the suffix “mp” added to the stereodescriptor to distinguish it from the absolute configuration of other stereogenic units.

In higher order [*n*]rotaxanes containing multiple oriented macrocycles encircling an oriented axle, the stereochemical situation is even more complicated (Fig. 24c). The advantage of considering the macrocycle plane as the stereogenic unit, as proposed by Takata, is that each macrocycle can be considered to act as a stereogenic unit, allowing the resultant diastereomers and enantiomers to be discussed in an analogous manner to covalent structures containing multiple stereocentres. Each macrocycle requires a stereodescriptor to specify the absolute stereochemistry of the system (Fig. 24c).^72^

#### Assignment of absolute stereochemistry

**Fig. 25:**
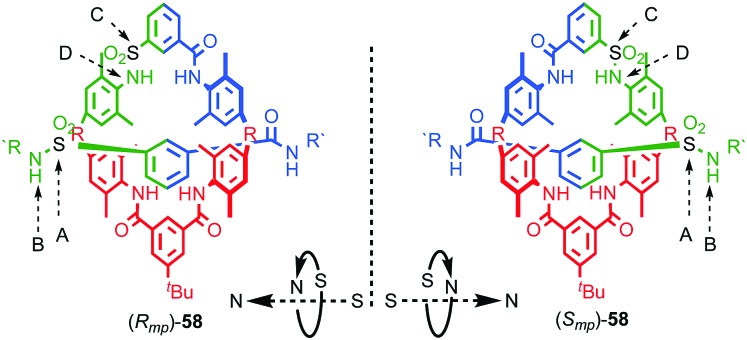
Vögtle's mechanically planar chiral rotaxane **58**.^75^ R = 1,1-cyclohexyl, R′ = –C_6_H_4_–Tr. Highest priority atoms in each component are highlighted in black.

Vögtle proposed a formal method for the assignment of absolute stereochemistry of mechanically planar chiral rotaxanes. This approach has largely been adopted by the community and is exemplified in the case of rotaxane **58** (Fig. 25).^73^ For each oriented *C*_*n*h_ ring in turn:

(i) Determine the atom of highest priority in the axle using the CIP system and label it “A”;

(ii) Exploring outwards from A, determine the highest priority atom that allows the axle direction to be assigned, again, using the CIP approach and label it “B”.

(iii) Repeat this process for the macrocycle, labelling the highest priority atom as “C” and the highest priority atom found when exploring outwards from C that allows direction to be determined as “D”.

(iv) View the assembly along the direction A → B and observe the orientation of C → D as either clockwise (*R*_mp_) or anticlockwise (*S*_mp_). Where A or C lie off the main chain of the axle or macrocycle respectively, the portion of the path from A → B or C → D that lies within the component under consideration is used to define the orientation.

#### Synthesis of mechanically planar chiral rotaxanes

The potential for this unusual form of stereoisomerism was first discussed by Schill in 1971,^71^ but it took almost three decades before Vögtle and Okamoto separated a racemic sample of rotaxane **58** using chiral stationary phase HPLC (CSP-HPLC; Fig. 25).^74^ The authors did not assign the stereochemistry of **58** as they could not determine the orientation of the macrocycle on the axle. Instead the labels (+) and (–) were applied to the isolated enantiomers according to the direction of their optical rotation.

**Fig. 26:**
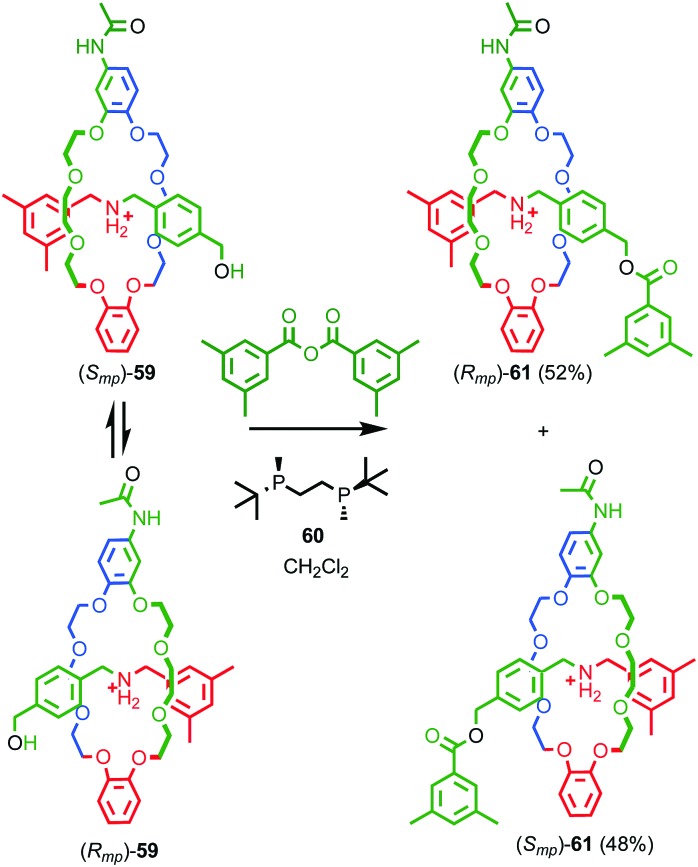
Takata's enantioselective dynamic kinetic resolution of pseudorotaxanes **59** to produce mechanically planar chiral rotaxane **61**.^72^ Counter ions omitted for clarity. Highest priority atoms in each component are highlighted in black.

Over the next decade Vögtle and co-workers continued to study mechanically planar chiral rotaxanes and their derivatives using CSP-HPLC to separate their enantiomers.^75^ However, this approach necessarily limits the scale on which compounds are available due to the cost, particularly historically, of high capacity CSP-HPLC columns. In 2007 Takata and co-workers disclosed an enantioselective approach rotaxanes using a dynamic kinetic resolution process (Fig. 26);^72^ enantiomeric pseudorotaxanes **59** were acylated using chiral phosphine catalyst **60** to give rotaxane **61** in up to 4.4% ee. The enantiomers of rotaxane **61** were subsequently separated using CSP-HPLC and characterised.

**Fig. 27:**
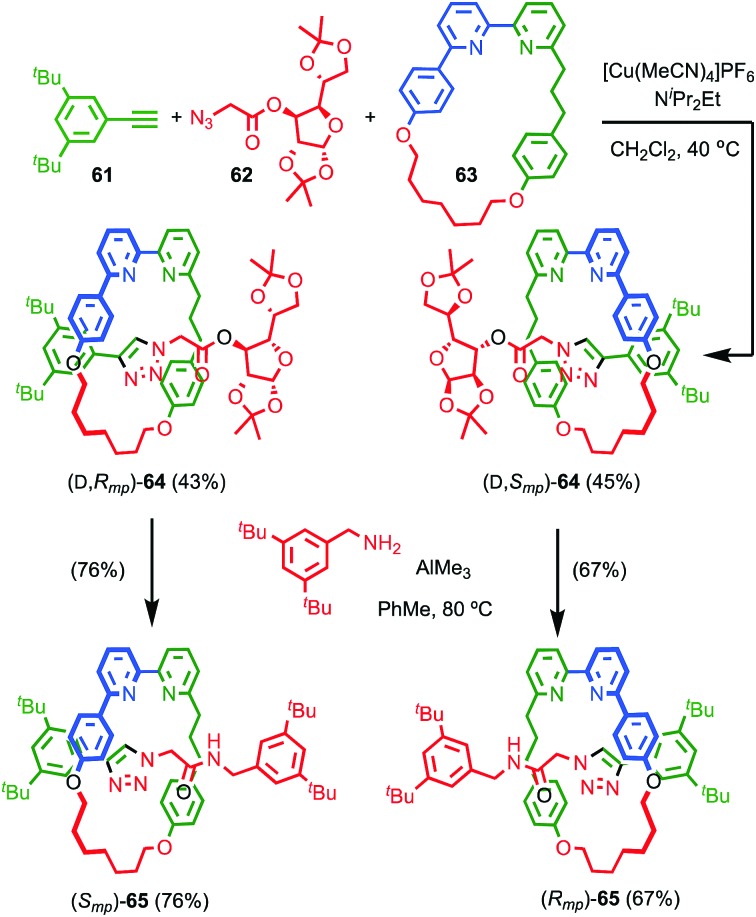
Goldup's chiral derivatisation approach to mechanically planar chiral rotaxanes **65**.^77^ Highest priority atoms in each component are highlighted in black.

Although Takata and co-worker's approach represents the first catalytic asymmetric synthesis of mechanically planar chiral rotaxanes, the low ee obtained prevents this method being used in a scalable synthetic manner. In 2014 Goldup and Bordoli demonstrated a scalable approach to mechanically planar chiral rotaxane **65** without the need for HPLC purification (Fig. 27).^76^ The approach relies on the use of enantiopure half-axle component **61** derived from d-glucose. Formation of the mechanical bond using Goldup's small macrocycle modification^46^ of the AT-CuAAC reaction^33^,^35^ produced a pair of mechanically epimeric diastereomers **64** that could be separated by flash chromatography.^77^,^78^ Diastereomeric rotaxanes **64** were subsequently crystallised, which allowed the mechanical stereochemistry to be assigned unambiguously, the first time this had been achieved. Subsequent aminolysis of the separated diastereomers of rotaxane **64**, a grafting process that preserves the mechanical bond,^79^ replaced the sugar-based stopper with an achiral amine to produce enantiopure rotaxanes (*S*_mp_)-**65** and (*R*_mp_)-**65** in which the mechanical bond is the sole stereogenic unit.

It is worth noting that although Goldup's use of a stopper containing covalent stereogenic units to derivatise a mechanically planar chiral rotaxane and allow the separation of its mechanical epimers, followed by removal of the covalent stereogenic unit to produce enantiopure rotaxanes is novel, mechanically epimeric rotaxanes themselves are not. Indeed, Vögtle and co-workers synthesised mechanically planar chiral rotaxanes stoppered by enantiopure sugars and even topologically chiral knots.^80^ However, in these cases the diastereomers were not separated. Even more common is for the mechanical component of the molecular asymmetry to be overlooked, or at least not highlighted, let alone the diastereomers separated.^81^ This points to diastereoisomerism being poorly expressed in these examples and is perhaps unsurprising; when the macrocycle is large or the axle is long the mechanical and covalent stereogenic units will typically interact weakly, making their separation or even the observation of diastereoisomerism by techniques such as ^1^H NMR difficult. Rotaxane **64** was thus designed specifically to be small and crowded to maximise the expression of the diastereoisomerism and allow the study and separation of the epimers.

However, one class of typically overlooked mixed mechanical-covalent diastereomers requires special mention. Any macrocycle bearing stereogenic centres in the backbone of the ring itself is by definition oriented (excluding *meso*-forms).^82^ Thus, rotaxanes formed from such macrocycles with *C*_*n*v_ axles will contain elements of both covalent and mechanical stereochemistry. The most obvious example of such macrocycles are the cyclodextrins which are ubiquitous in the chemistry of the mechanical bond, particularly in examples of mechanically interlocked polymers.^15^

**Fig. 28:**
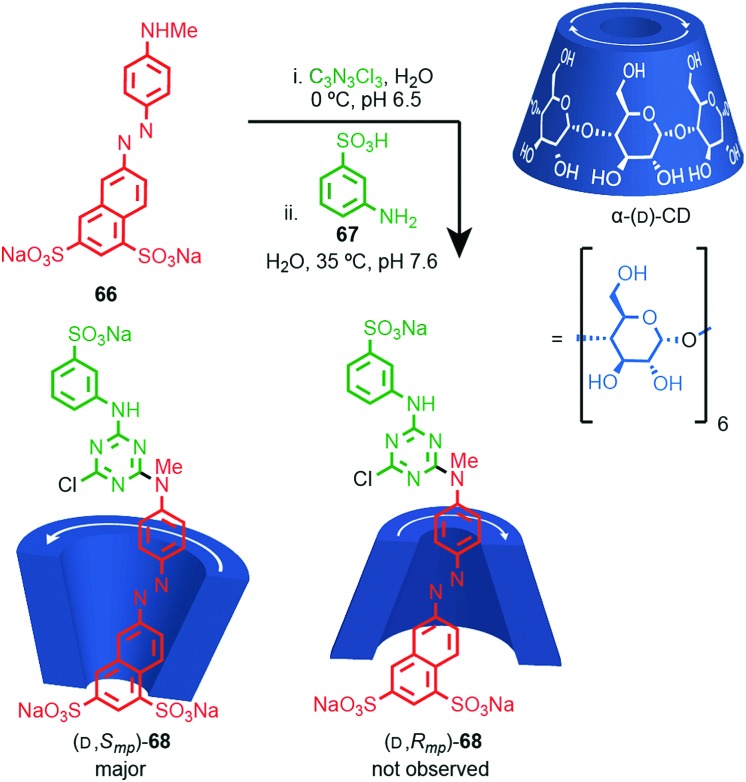
Anderson's selective synthesis of one epimer of rotaxane (d,*S*_mp_)-**68** and the structure of alternative mechanical epimer (d,*S*_mp_)-**68** (not observed).84*a* Highest priority atoms in each component are highlighted in black.

Most applications of cyclodextrin-based rotaxanes do not invoke their chirality and to our knowledge, the mechanical planar stereogenic component of their isomerism has not been highlighted previously. However, it is obviously related to their cone shape and rotaxanes have been synthesised where the cyclodextrin ring is threaded onto the axle in a specific orientation,^83^ as in Anderson and co-workers’ synthesis of azo-dye rotaxane (d,*S*_mp_)-**68**, formed from the inclusion complex between α-(d)-CD and pre-axle **66** stoppered by reaction with trichloro-1,3,5-triazine and aniline **67** (Fig. 28).84*a* Rotaxane **68** is preferentially formed as the (d,*S*_mp_) isomer over the (d,*R*_mp_) diastereomer. Although these are typically thought of as orientational isomers,^84^ presumably due to the pronounced cone shape of the macrocycles, we suggest that they are more accurately described as mechanically planar chiral diastereomers, making the synthesis and separation of cyclodextrin-based mechanical epimers one of the earliest and most common examples of the stereoselective synthesis of mechanically planar chiral rotaxanes!

**Fig. 29:**
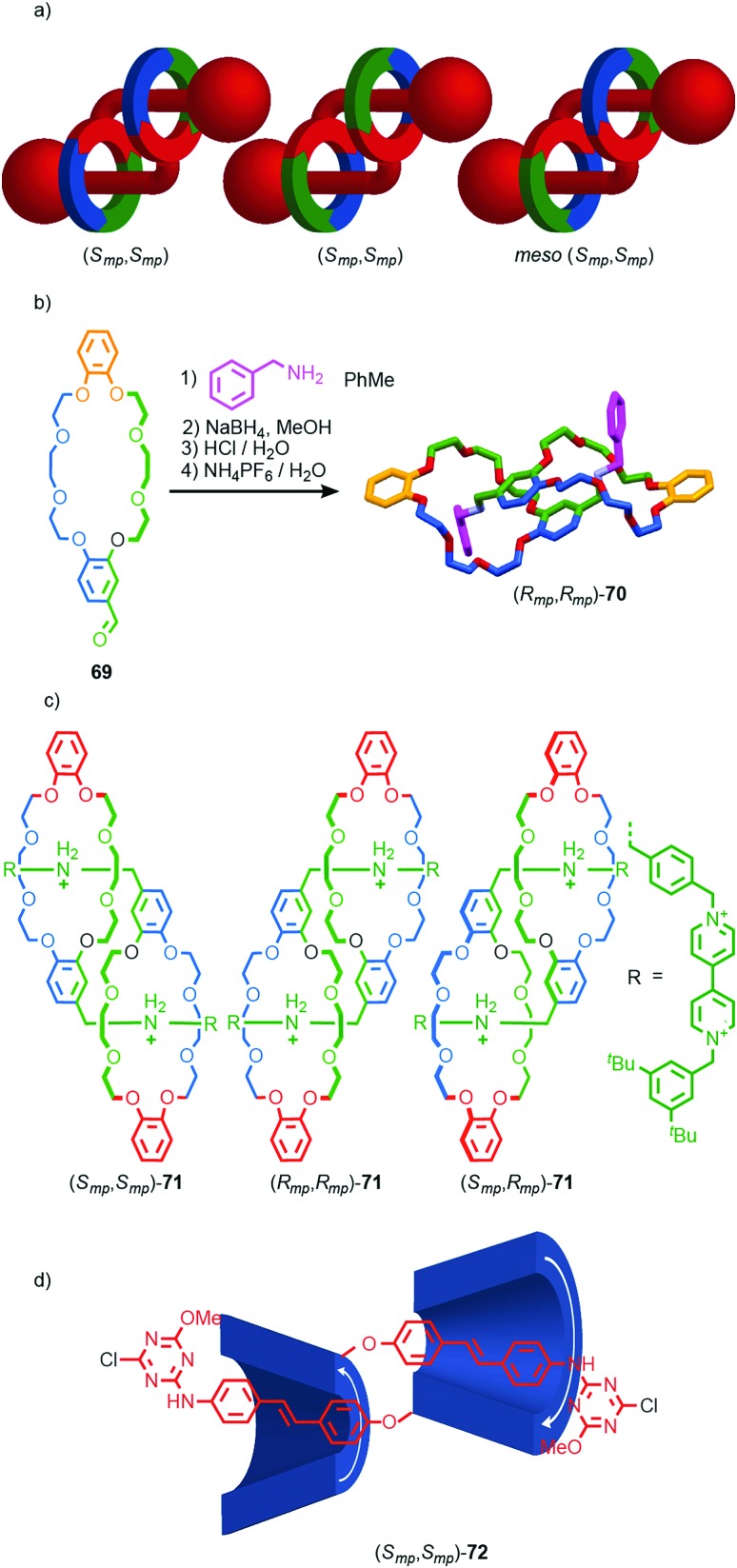
(a) Cartoon representations of the stereoisomers of homo-dimeric [*c*2]daisy chain rotaxanes (arbitrary stereodescriptors included). (b) Solid-state structure of pseudo[*c*2]daisy rotaxane **70**.^86^ (c) Mixture of [*c*2]daisy diastereomers **71** reported by Stoddart and co-workers.^87^ (d) Easton's [*c*2]daisy **72** which is formed as a single stereoisomer.^88^ Highest priority atoms in each component are highlighted in black. In the case of **70**, the ether oxygen that is highest priority in the daisy chain is indicated in macrocycle **69**.

Finally, mechanical planar chirality can also arise in the formation of daisy chain-type rotaxanes when the two rings are oriented. This stereoisomerism arises even when the structure is homo-dimeric leading to a *C*_2_ symmetric diastereomer that exists as a pair of enantiomers and a *C*_i_ symmetry diastereomer that is *meso* (Fig. 29a). Stoddart and co-workers initially observed this form of stereoisomerism in the formation of pseudo [*c*2]daisy rotaxane **70** from building block **69** (Fig. 29b).^85^ In the solid state only the *C*_2_ diastereomer was observed as a racemic mixture. However, in later work, stimuli responsive [*c*2]daisy rotaxane **71** was found to form as a statistical mixture of stereoisomers in solution (Fig. 29c).^86^ The method for assigning absolute stereochemistry in mechanically planar chiral rotaxanes can be extended to [*c*2]daisy rotaxanes and related structures but in this case only one highest priority atom need be identified and labelled “A”. The orientation of the axle can then be defined by exploring outward from this highest priority atom until the highest priority point of difference is identified that orients the axle portion of the molecule and labelling it “B”. The same procedure can be applied to the ring to identify atom “C”. The stereochemistry is then assigned as with simple mechanically chiral rotaxanes by viewing the molecule along the axis A → B and observing the orientation of the vector A → C. Where the highest priority atom lies outside the main chain of one of the components (almost always the case), the orientation of the axle and ring are defined by considering the portion of the path from A to B or A to C respectively that lies within that component.

Coutrot and co-workers observed similar behaviour in the formation of a [*c*2]daisy rotaxane stoppered by enantiopure sugar units. In this case, no diastereoselectivity between the *C*_2_ diastereoisomers was observed as a result of the interaction of the stereochemistry of the stopper units with the stereogenic mechanical bond during the self-assembly process. Conversely, Easton and co-workers observed complete stereocontrol in the formation of cyclodextrin-based [*c*2]daisy rotaxane (*S*_mp_,*S*_mp_)-**72** (Fig. 29d), demonstrating that the interaction between covalent and mechanical stereogenic elements can direct for the formation of such complex diastereomers.^87^

#### Properties and applications of mechanically planar chiral rotaxanes

**Fig. 30:**
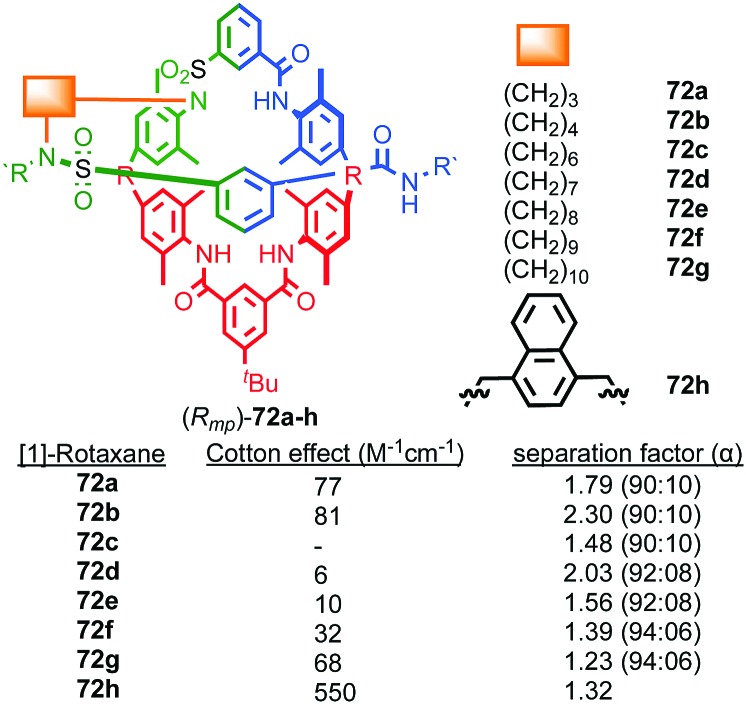
The effect of structure on the Cotton effect of rotaxanes **72** (*R*_mp_ stereoisomer shown for illustrative purposes).^74^ R = 1,1-cyclohexyl, R′ = –C_6_H_4_–Tr. Highest priority atoms in each component are highlighted in black.

The synthesis of mechanically planar chiral rotaxanes in enantiopure form remains challenging, with most syntheses relying on CSP-HPLC to separate a racemic mixture. Using this approach, Vögtle and co-workers studied a homologous series of enantiopure mechanically planar chiral [1]rotaxanes by CD (Fig. 30).73a,^88^ In the first series studied (**72a–g**) no clear link was observed between the size of the Cotton effect and the length of the aliphatic bridge between the macrocycle and axle although simplistically one might expect the shorter aliphatic link to lead to a more rigid structure and better expressed molecular asymmetry resulting in larger Cotton effects. Indeed, parent [2]rotaxane **58**, which is expected to display significant co-conformational mobility, exhibits a larger Cotton effect than [1]rotaxane **73**. Similarly, the separation factors in the HPLC purification of the enantiomers failed to show any significant trend. Later work in which rigid, chromophoric units were introduced as the cross-link led to clearer trends;73*b* larger Cotton effects were observed when the bridge contained a chromophoric unit and was relatively rigid. Remarkably, [1]rotaxane **72h** that contains a short, rigid naphthalene linker displays a large molar CD of the order of 500 M^–1^ cm^–1^, a value more commonly associated with helicenes with their large π-surface (Fig. 30).

**Fig. 31:**
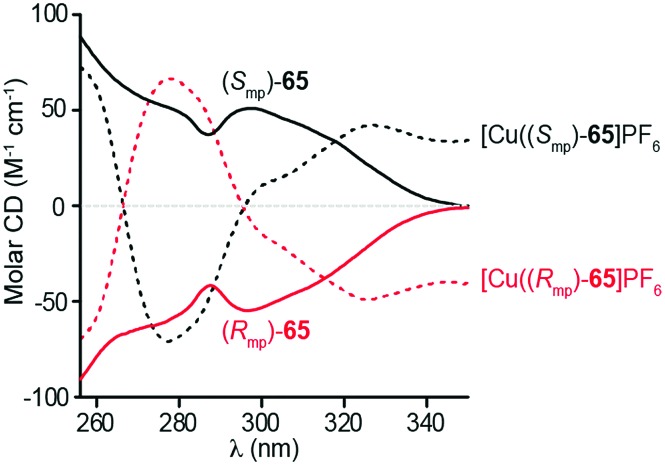
Chiroptical switching through metal binding in Goldup's mechanical planar chiral rotaxane **65**.^77^ Reproduced with permission from ref. 77. Copyright 2014 American Chemical Society.

Goldup and co-workers also studied the CD spectra (Fig. 31) of enantiopure [2]rotaxane 65 isolated using their chiral derivatisation approach (Fig. 27).^77^ Although the enantiomers of rotaxane 65 display mirror image CD spectra that are relatively featureless, binding of Cu^I^ into the cavity of the rotaxane introduces a metal–ligand transfer band and the appearance of strong Cotton effects with two sign inversions. Notably, binding of the metal ion inverts the sign of the CD signal at several wavelengths, suggesting that mechanically planar chiral metal complexes have potential applications in chiroptical switching.

**Fig. 32:**
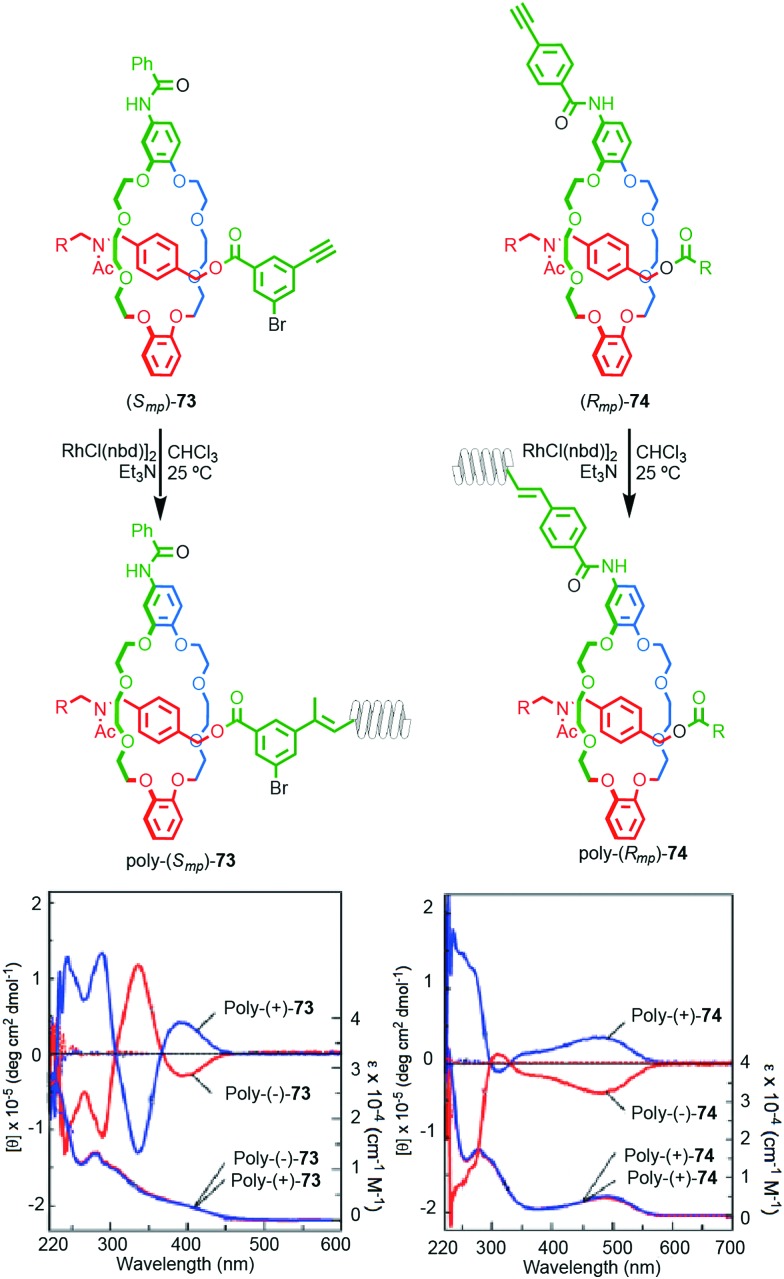
Takata's mechanically planar chiral helical polymers poly-**73** and poly-**74**.^90^ Stereoisomers shown and assigned for illustrative purposes. R = 3,5-Me_2_-C_6_H_3_. Highest priority atoms in each component are highlighted in black. Reproduced with permission from ref. 90. Copyright 2017 John Wiley and Sons.

Takata and co-workers recently reported the synthesis of polyacetylene materials in which a mechanically planar chiral unit on the side chain of the polymer induces a helical conformation on the polymer chain (Fig. 32).^89^ Racemic samples of alkyne substituted rotaxanes **77** and **74** were separated using preparative CSP-HPLC and the separated enantiomers polymerised by the action of a Rh^II^ catalyst. The resulting enantiomeric polymers displayed strong mirror image Cotton effects on the main chain UV absorbance below 450 nm, consistent with the polymer chain adopting a helical conformation. Interestingly, significant helicity was observed whether the polymer was attached to the macrocycle (**74**) or axle (**73**) moiety.

**Fig. 33:**
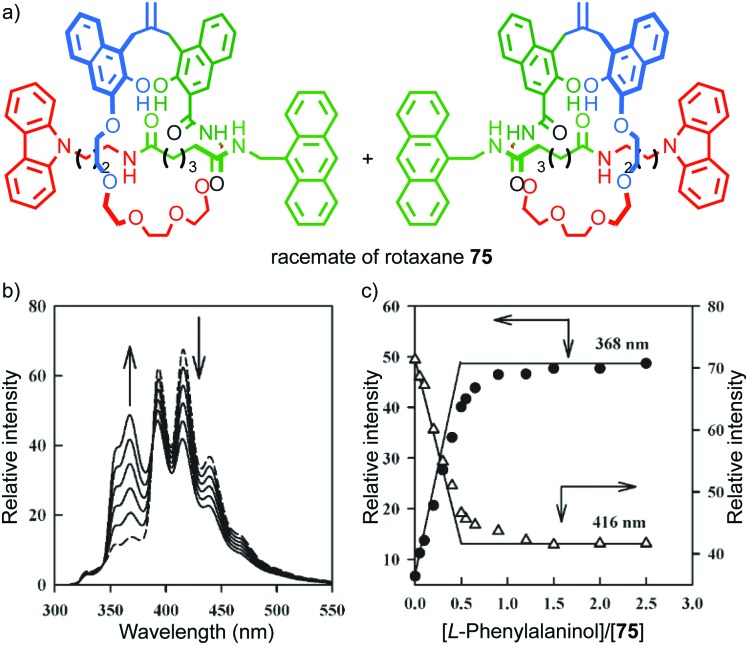
(a) Kameta and Hiratni's chiral host **75**.^91^ Highest priority atoms in each component are highlighted in black. (b) The effect of (l)-phenylalalinol on the fluorescence of **75**. (c) A plot of the emission intensity of rotaxane **75** with respect to equivalents of (l)-phenylalalinol. Reproduced from ref. 91 with permission from The Royal Society for Chemistry.

In 2004 Kameta, Hiratani and co-workers reported a mechanically planar chiral rotaxane as a diastereoselective host for enantiopure phenyl alalinol (Fig. 33).^90^ The authors reported that a racemic sample of rotaxane **75**, in the presence of 0.5 equivalents of (l)-phenyl alaninol, displayed two sets of resonances by ^1^H NMR, one consistent with the starting material and another that was assigned as a host–guest complex. Similarly, monitoring of the titration by fluorescence led to a curve consistent with binding of the guest and suggestive of a turning point when 0.5 equivalents of guest was added. The authors proposed that their results are consistent with selective binding of the guest to one hand of the host to produce a single diastereoisomeric complex. However, the data presented are not conclusive as the enantiopure hosts were not tested individually, although CSP-HPLC separation of the enantiomers was reported.

#### Conclusions

Since the seminal work of Vögtle and Okamoto progress in the study of mechanically chiral rotaxanes has remained relatively slow. With the exception of Goldup and co-workers’ synthesis of rotaxane **65**, enantiopure mechanically planar chiral rotaxanes are typically prepared using CSP-HPLC or are investigated as their racemate, neither of which is conducive to large scale applications or study. The surge in recent interest, combined with new methods for their synthesis, suggests that these molecules are experiencing a renaissance that will lead to further information about their interesting chiroptical and host–guest properties, along with potential applications in catalysis and sensing. Furthermore, although it has been largely overlooked, the role of mechanical planar chirality in the properties of cyclodextrin-derived rotaxanes is ripe for investigation.

### Topologically chiral catenanes

#### Description of the mechanical stereogenic unit

**Fig. 34:**
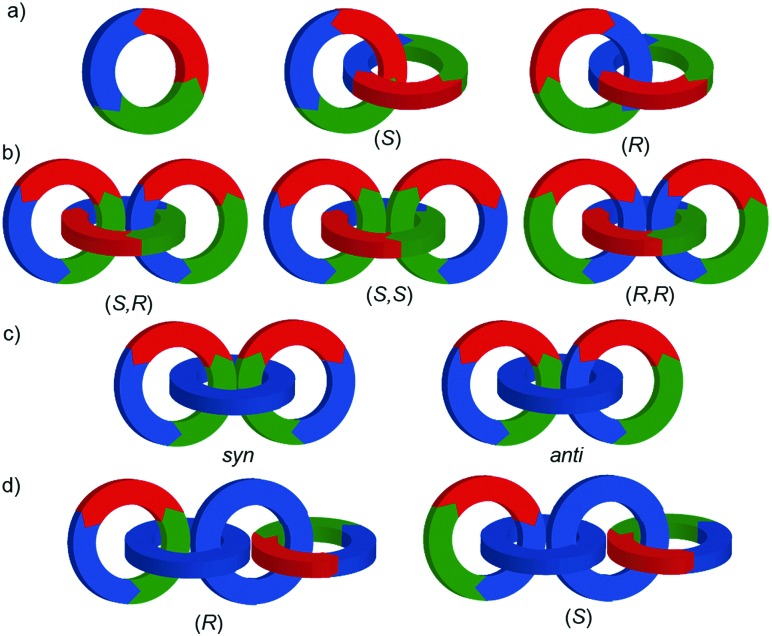
Schematic representations with arbitrary stereodescriptors of (a) topologically chiral homo [2]catenane and the constituent macrocycle, (b) the stereoisomers of a topologically chiral homo[3]catenane, (c) the topological diastereomers of an achiral linear [3]catenane in which only the terminal rings are oriented, (d) the enantiomers of a chiral linear [4]catenane in which only the terminal rings are oriented.

When two oriented rings of *C*_*n*h_ symmetry are combined to form a [2]catenane the resultant structure is chiral (Fig. 34). This stereogenic element is maintained under all topologically permitted deformations and so this form of chirality has been recognised and termed “topological” and molecules that display this form of stereochemistry have been termed “topologically chiral catenanes”. As with the oriented rings of mechanically planar chiral rotaxanes, although *C*_*n*h_ symmetry is present when multiple repeating oriented units are combined to form the macrocycle, most examples of topologically chiral catenanes are composed of *C*_1h_ symmetry rings.

Considering how mechanical bond formation affects the improper symmetry operations of the components, linking two *C*_*n*h_ rings in a catenane results in a structure in which the mirror planes of the individual components can never become aligned and thus the assembly is chiral. Linking multiple *C*_*n*h_ symmetric macrocycles to form a linear [*n*]catenane results in one additional stereogenic unit per mechanical bond and thus complex stereoisomers. Conversely, linear [*n*]catenanes where only the terminal rings are oriented are chiral when *n* is even and achiral when *n* is odd. It should be noted however, that [3]catenanes with oriented peripheral rings exist as achiral diastereomers. These can be described as *syn* if the two peripheral rings are oriented in the same direction or *anti* if they are oriented in the opposite sense. This topological diastereoisomerism has been studied by Stoddart and co-workers.^91^

#### Assignment of absolute stereochemistry

The potential for catenanes to display topological chirality was discussed as early as 1961 by Wasserman and Frisch in their seminal work on chemical toplogy,13*a* and the first example of a topologically chiral catenane was described by Sauvage and co-workers in 1988.^92^ However, to date the absolute stereochemistry of isolated samples of enantiomers has yet to be assigned. Perhaps as a result, there are no generally accepted clear guidelines for determining a stereochemical label in such molecules.

**Fig. 35:**
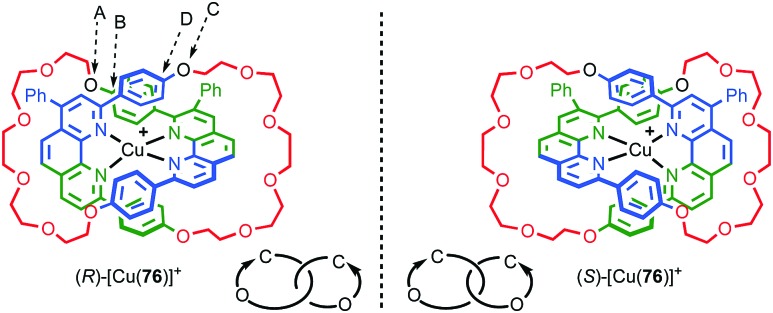
Schematic representation of topologically chiral catenane complex [Cu(**76**)]. Counter ions omitted for clarity.^93^,^97^ Highest priority atoms in each component are highlighted in black.

However, a number of authors, including Vögtle^93^,^94^ and Stoddart10g,^95^ have proposed methods for assigning the stereochemistry of topologically chiral catenanes and these largely agree with one another. Based on these previous suggestions, here we explicitly outline a general approach to assigning the absolute stereochemistry of topologically chiral catenanes, using catenane **76** as an exemplar (Fig. 35). For each oriented ring in turn:

(i) Determine the atom of highest priority using the CIP system and label it as “A”.

(ii) Exploring outwards from A, determine the highest priority atom that allows direction to be assigned again, using the CIP approach, and label it “B”.

(iii) Using A and B define the orientation of the rings by a polar vector running in the direction A → B. Where A lies off the main chain of the macrocycle, the portion of the path from A → B that lies within the ring is used to define the orientation.

(iv) View the catenane with the A → B vector of one ring passing through the cavity of the other and away from the observer.

(v) Observe the orientation of the A → B vector of the second ring; a clockwise orientation is assigned as *R* and an anticlockwise orientation is assigned as *S*.

It should be noted that the same outcome is obtained regardless of which role a given ring plays. Thus, although this method bears a significant resemblance to the rules for assignment of stereochemistry in mechanically planar chiral rotaxanes where the stereogenic unit can be considered as arising by the axle desymmetrising an oriented macrocycle, the stereogenic unit of a topologically chiral catenane is best considered to be the overall link.

The approach presented can be extended to complex [*n*]catenanes composed of *n* oriented rings in which the stereochemistry at each ring intersection can be assigned unambiguously for both linear and branched analogues. This methodology can readily be extended to esoteric examples such as linear [4]catenanes with oriented rings at the extremities of the chain either by considering a notional system in which the oriented rings are interpenetrated, or more simply, by orienting the system as above using the closest approach of the A → B vector of one ring to the other ring to orient the observer. The stereochemistry is then determined by considering the orientation of the A → B vector of the second ring.

#### Synthesis

In 1988 Sauvage and co-workers reported the synthesis of a racemic sample of topologically chiral catenane **76** as its Cu^I^ complex using their phenanthroline-Cu^I^ template.^93^ Although they were able to demonstrate the chirality of [Cu^I^(**76**)]^+^ by ^1^H NMR using Pirkle's reagent to induce splitting of key signals into diastereomeric sets, it took a further five years before [Cu^I^(**76**)]^+^ was isolated in enantiopure form using CSP-HPLC.^96^ The separated enantiomers were assigned as (+)-**76** and (–)-**76** based on the direction in which they rotate plane polarised light as the absolute stereochemistry of **76** could not be determined.

**Fig. 36:**
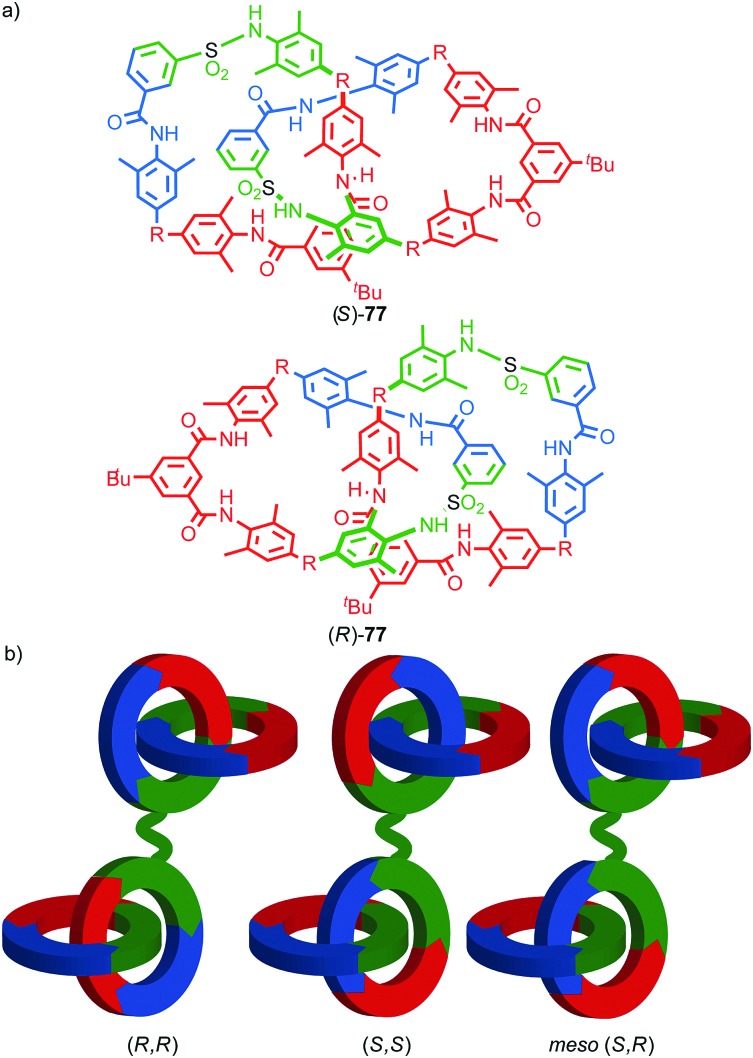
(a) Vögtle and co-workers neutral topologically chiral catenane **77**.^75^ (b) Schematic representation of the stereoisomers of cross-linked analogues of catenane **77**.^93^,^94^ R = 1,1-cyclohexyl. Highest priority atoms in each component are highlighted in black.

Attempts to separate the enantiomers of metal-free catenane **76** by CSP-HPLC failed, in part due to poor solubility, although at the time it was also intimated that the metal free catenane experiences significantly greater co-conformational freedom which might decrease the expression of topological chirality. However, in 1997, Vögtle and co-workers separated the neutral enantiomers of catenane **77** (Fig. 36a),^75^ demonstrating that even relatively co-conformationally flexible topologically chiral catenanes can be resolved using standard CSP-HPLC techniques.^97^ Using their CSP-HPLC approach, Vögtle and co-workers studied the optical properties of topologically chiral catenanes, demonstrating that they exhibit a significant CD response, and produced a number of derivatives including the products of cross linking the macrocycles of catenane **77** to give molecules they christened “pretzelanes”,^75^,^95^ and chiral handcuff catenanes that exist as chiral and *meso* diastereomers (Fig. 36b).^94^

Sauvage and Vögtle clearly demonstrated the synthesis and separation of topologically chiral catenane enantiomers. However, the use of CSP-HPLC is necessarily limiting both in terms of scale and generality, and to date no general methodological approach to purely topologically chiral catenanes in enantiopure form has been proposed or demonstrated. However, there have been a number of diastereoselective syntheses of catenanes containing elements of covalent and topological chirality.

**Fig. 37:**
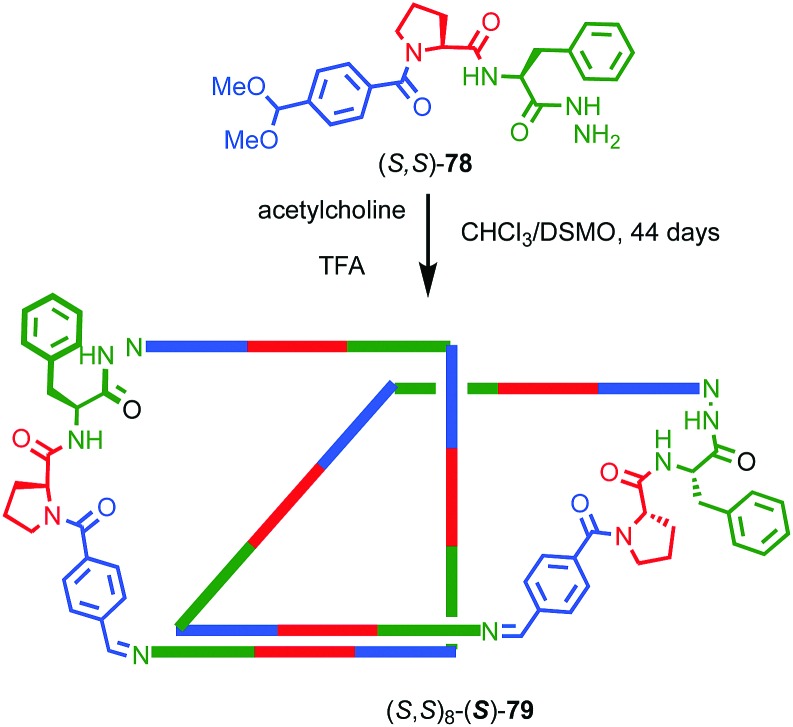
Sanders acetylcholine-host catenane (*S*,*S*)_8_-(S)-**79** assembled by hydrazone formation under dynamic conditions in the presence of the guest. Single diastereomer shown is for illustrative purposes; the absolute configuration of the product was not determined. (*S*,*S*) stereodescriptors refer to all covalent stereocentres in macrocycle framework. Emboldened ***S*** descriptor refers to the topological stereogenic unit. Highest priority atoms in each component are highlighted in black.

As previously discussed, a macrocycle that contains one or more stereogenic centres in the ring structure is by definition oriented, as long as the macrocycle is not *meso*. In 2005 Sanders and co-workers reported the isolation of catenane **79** from the dynamic covalent self-assembly of simple enantiopure peptide building block **78** in the presence of acetylcholine (Fig. 37); over 44 days HPLC analysis confirmed the emergence of catenane **79** from a reaction mixture that was initially dominated by short linear oligomers.^98^ On a preparative scale catenane **79** was isolated in 68% yield. ^1^H NMR analysis of **79** in the presence of acetylcholine revealed sharp resonances suggesting that a single diastereomer of **79** had formed and thus that covalent chirality in the building block was able to direct the formation of a single topologically chiral epimer. However, it was not possible to identify which epimer had formed.

**Fig. 38:**
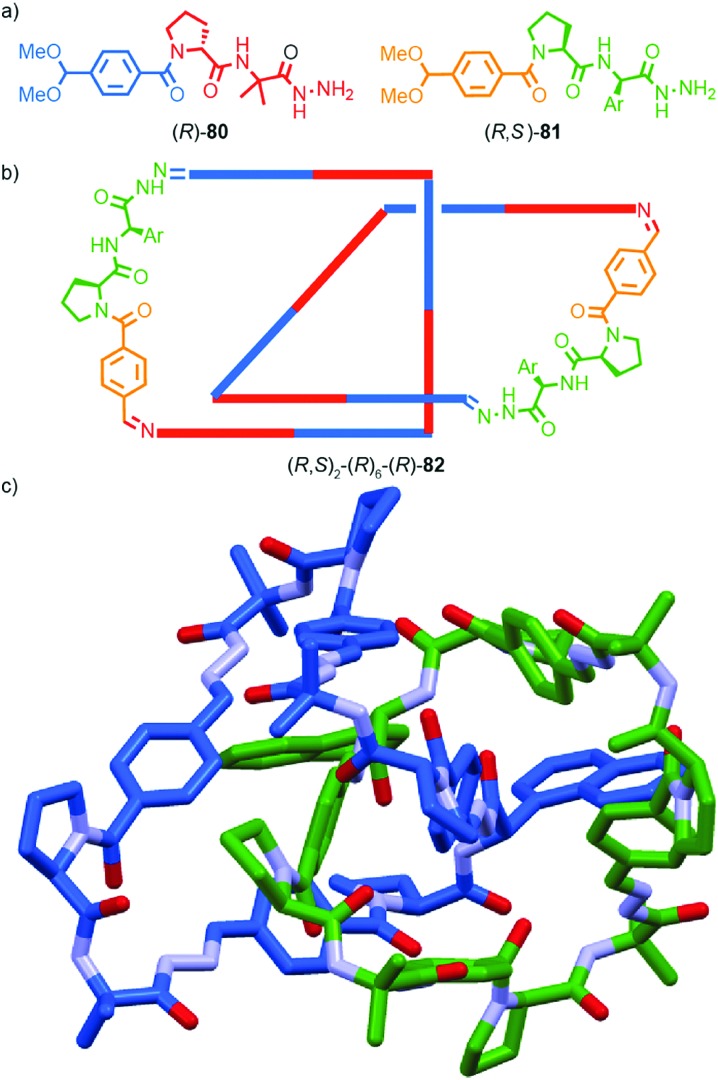
Gagne and co-workers diastereoselective assembly of catenane (*R*,*S*)-(*R*)_3_-(*R*)-**82**. (a) Building blocks (*R*)-**80** and (*R*,*S*)-**81**. (b) Schematic representation of (*R*,*S*)_2_-(*R*)_6_-(*R*)-**82** (*R* and *R*, *S* refer to stereochemistry of repeating covalent fragments derived from (*R*)-**80** and (*R*,*S*)-**81** respectively; (*R*) refers to the topological stereogenic unit). (c) Solid state structure of catenane (*R*,*S*)_2_-(*R*)_6_-(*R*)-**82** demonstrating the formation of a single diastereomer. Ar = naphthyl. Highest priority atoms in each component are highlighted in black.

A similar effect was observed by Gagné and co-workers in the dynamic co-assembly of enantiopure building blocks (*R*)-**80** and (*R*,*S*)-**81** (Fig. 38a).^99^ HPLC-MS analysis suggested that the major product of the dimeric library was an octameric species containing (*R*)-**80** and (*R*,*S*)-**81** in a 3 : 1 ratio and the fragmentation pattern of this major product contained a single tetrameric ion composed of these components in the same 3 : 1 ratio. Optimisation of the reaction conditions allowed isolation of the octameric product in 64% yield. As in the case of **79**, ^1^H NMR analysis revealed sharp signals consistent with the formation of a single topological diastereoisomer. Single crystal X-ray analysis allowed the product to identified as catenane **82** assembled from two macrocycles, themselves comprised of (*R*)-**80** and (*R*,*S*)-**81** in a 3 : 1 ratio, as predicted by MS (Fig. 38b). Strikingly, **82** was assembled as a single topologically chiral diastereomer. Using the approach outlined above we can assign the observed diastereomer formed as (*R*,*S*)_2_-(*R*)_6_-(***R***)-**82**, although the absolute stereochemistry of the mechanical bond was not assigned by the authors.

**Fig. 39:**
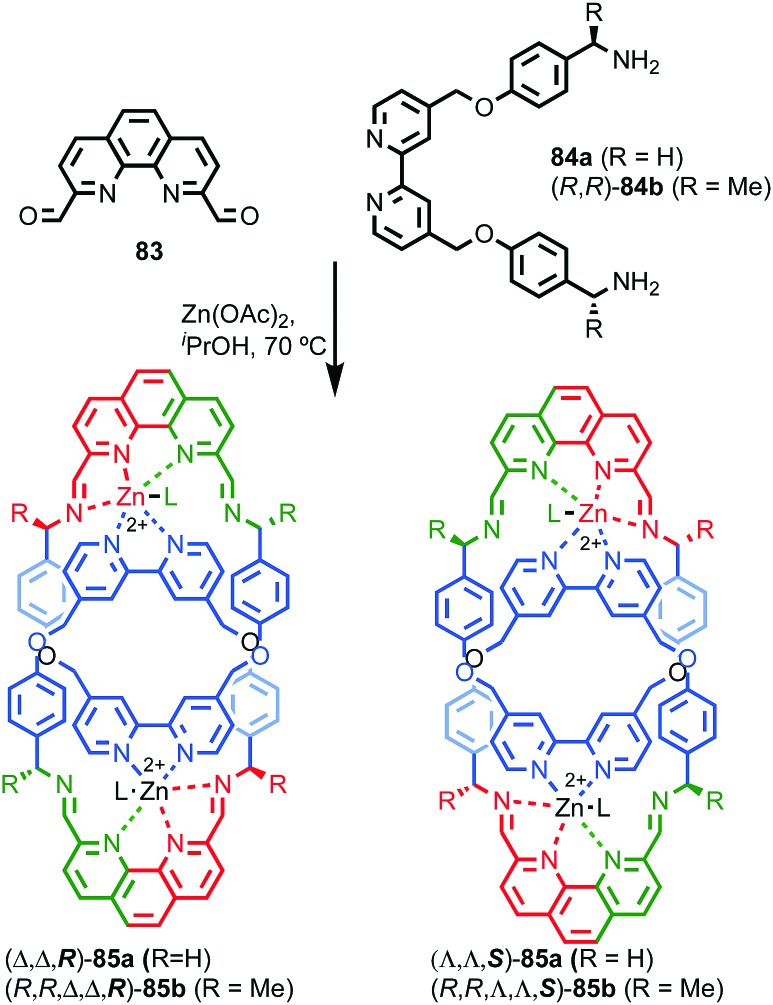
Trabolsi's dynamically chiral catenanes **85**.^101^ Counter ions omitted for clarity. (*R* and Λ/Δ) stereodescriptors refer to covalent and at-metal stereochemistry respectively. Emboldened stereodescriptor refers to the topological stereogenic unit. Highest priority atoms in each component are highlighted in black.

Catenanes **79** and (*S*,*S*)_3_-(*R*,*S*)-(*R*)-**82** are assembled using dynamic covalent chemistry under thermodynamic control and demonstrate the power of this approach for the synthesis of diastereoisomers. More recently, Trabolsi and co-workers disclosed catenane metal complexes **85a** and **85b** that are assembled under thermodynamic control through imine formation combined with metal–ligand interactions (Fig. 39).^100^ Catenane **85a** is assembled from building blocks **83** and **84a** in the presence of Zn(OAc)_2_. The solid-state structure of **85a** confirms the interlocked nature of the product and reveals that the coordination environment of the Zn ion is comprised of 2 × N donors from the 4,4′-bipyridine unit, 2 × N donors from the phenanthroline unit, one of the imine moieties and a trifluoroacetate anion, resulting in a highly distorted octahedral geometry and an at-metal stereocentre.

Coordination of one of the imine units to the metal ion orients the macrocycle, resulting in an element of topological stereochemistry, and thus catenane **85a** can exist as three diastereomeric pairs of enantiomers. In the solid state, **85a** crystallises as the racemate of the (Δ,Δ,*R*)* diastereoisomer in which both metallic stereocentres have the same absolute configuration, demonstrating an interplay between at-metal stereochemistry and topological stereochemistry at least in the solid state. In solution at room temperature, the ^1^H NMR spectrum of **85a** reveals broad signals consistent with a more symmetrical structure, suggesting that **85a** exists as a dynamic mixture of diastereomers in fast exchange. Cooling led first to coalescence and then the emergence of sharp signals consistent with a species of lower symmetry, suggesting the slowing of exchange between diastereomers at lower temperature.

When building block **84a** is replaced with enantiopure amine (*R*,*R*)-**84b** the product catenane, **85b**, contains elements of topological, at-metal and carbon-centred stereochemistry. Strikingly, in the solid state, only the (*R*,*R*,Δ,Δ,*R*) diastereomer of the four possible stereoisomers is observed, demonstrating once again that covalent stereochemistry can direct the stereoselective formation of a topological stereogenic element. In solution, the ^1^H NMR spectrum of **85b** is relatively sharp, suggesting that the methyl substituent on the amine building block significantly slows the exchange between diastereomers. In keeping with this, at higher temperatures, the two signals corresponding to the methyl protons coalesce, once again suggesting that although **85b** crystallises as a single diastereomer, in solution a dynamic equilibrium exists between different stereoisomers.

#### Conclusions

Although the potential for topological chirality in catenanes was identified very early in the discussion of interlocked molecules,13*a* little is known about their properties beyond that some examples display large Cotton effects and nothing is known about their potential applications. Given recent advances in methods for the synthesis of catenanes, as well as improvements in CSP-HPLC technologies, hopefully this situation will change in the near future. In the longer term, the development of stereoselective methods for the synthesis of topologically chiral catenanes will be required if this unusual form of stereochemistry is to be exploited fully. Finally, as can be seen in the cases of **79**, **82** and **85b**, the assignment of absolute stereochemistry is complicated when multiple labels are required. For this reason, we would propose that the suffix “mt” be added to the topological element to signify “mechanical topological” stereochemistry.

### Mechanically axially chiral catenanes

#### Description of the stereogenic unit

**Fig. 40:**
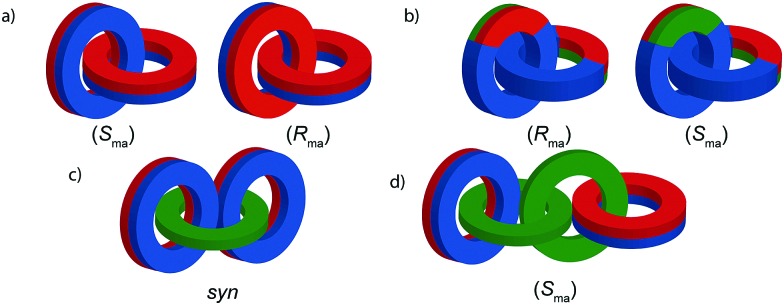
Schematic representation of the enantiomers of [2]catenanes based on (a) two equivalent *C*_∞v_ rings, (b) two equivalent *C*_1v_ rings, (c) the *syn* diastereomer of an achiral [3]catenane with terminal *C*_∞v_ rings, (d) one enantiomer of a linear [4]catenane with terminal *C*_∞v_ rings.

When two macrocycles of *C*_*n*v_ symmetry (principle axis perpendicular to the macrocycle plane), are combined to form a [2]catenane the resulting assembly is chiral even when the rings are identical.^101^*C*_*n*v_ symmetry is achieved when the two faces of the ring are inequivalent and in chemical terms, one can consider the required facial asymmetry as arising from the projection of substituents above and below the macrocyclic plane. Although the highest symmetry macrocycle that meets the requirement of facial asymmetry has *C*_∞v_ symmetry, (Fig. 40a), the majority of such chiral catenanes are composed of two *C*_1v_ macrocycles (Fig. 40b) whose faces are distinguished by the substituents of a single tetrahedral atom. The symmetry properties of such catenanes are similar to that of an axially chiral allene and the designation “mechanically axially chiral” has been proposed by Stoddart and Bruns with an “ma” added to the stereodescriptor.10*g* Considering the effect on the local symmetry of the components of mechanical bond formation, the relative orientation of the two rings in the catenane is restricted such that no representation is possible in which the mirror planes of individual macrocycles can be made coincident. Thus, the improper symmetry elements of the components are not present in the interlocked structure and it is chiral.

Regardless of the chemical structure of the macrocycles, it should be noted that mechanical axial chirality is Euclidean in origin, rather than topological, as it is always possible to exchange mechanically axial chiral enantiomers if the Euclidean properties of the molecule are relaxed. In a similar manner to the topologically chiral [*n*]catenanes discussed above, linear [*n*]catenanes with terminal facially unsymmetrical macrocycles are chiral when *n* is even (Fig. 40d), and achiral when *n* is odd (Fig. 40c). In the latter case the molecule can exist as a *syn* or *anti* diastereomer.

#### Assignment of absolute stereochemistry

Stoddart and Bruns suggested that the assignment of the absolute stereochemistry of mechanically axial catenanes can be achieved by extending the accepted approach for covalent axial chirality. Their approach is outlined below and exemplified for catenane **86**:

(i) For each ring, assign the priority of the macrocycle faces by identifying the highest priority (CIP) atom that does not lie in the macrocycle plane (and cannot be exchanged between faces by conformational changes), and label it “A”.

(ii) Define a vector from A to the macrocycle plane for each ring.

(iii) View the relative orientation of these vectors at the extremities of the assembly.

(iv) Consider the direction of rotation from the head of the front vector to the tail of the rear vector, with a right-handed rotation assigned as (*R*_ma_) and a left-handed path assigned as (*S*_ma_).

As with topologically chiral catenanes, this approach can readily be extended to linear [4]catenanes in which the two terminal rings have *C*_*n*v_ symmetry.

#### Synthesis of mechanically axially chiral catenanes

**Fig. 41:**
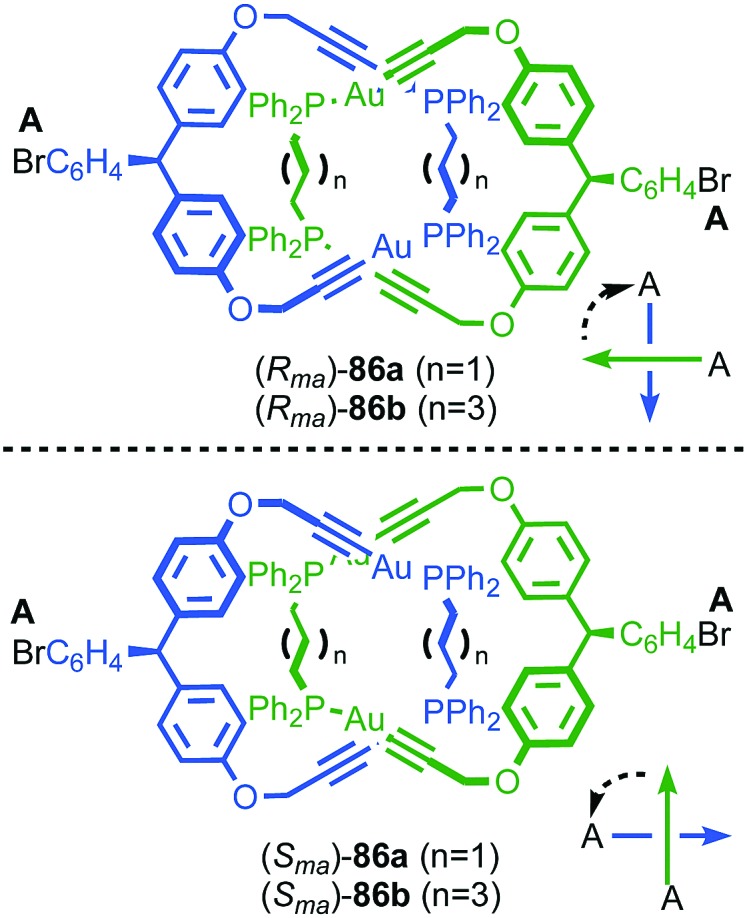
Puddephat's mechanically axially chiral catenane **86**.^103^ Highest priority off-ring plane atoms in each component are highlighted in black.

The first mechanically axially chiral [2]catenanes were reported by Puddephat and co-workers in 2002.^102^ Catenanes **86** were reported to assemble through a combination of Au^I^–Au^I^ aurophilic and π–π stacking interactions, with the facial asymmetry of the macrocycles arising from the substituent on the “hinge” region of the ring (Fig. 41). The chirality of catenanes **86** was studied by examining the equivalence or otherwise of the phosphorous centres by ^31^P NMR.^103^ Although the smaller of the two molecules, **86a** showed an AB quartet, consistent with the expected *C*_2_ symmetry of the chiral catenane, the phosphorous centres of **86b** produced a simple singlet, suggesting the facial asymmetry was poorly expressed in the larger, more flexible system. The ^1^H NMR spectra of **86a** and **86b** did not exhibit the expected diastereotopicity and the addition of chiral shift reagents failed to give rise to diastereoisomeric signals in the ^1^H spectra of **86**. These results suggest that the molecular asymmetry of catenanes **86** is poorly expressed in the shape of the molecules.

**Fig. 42:**
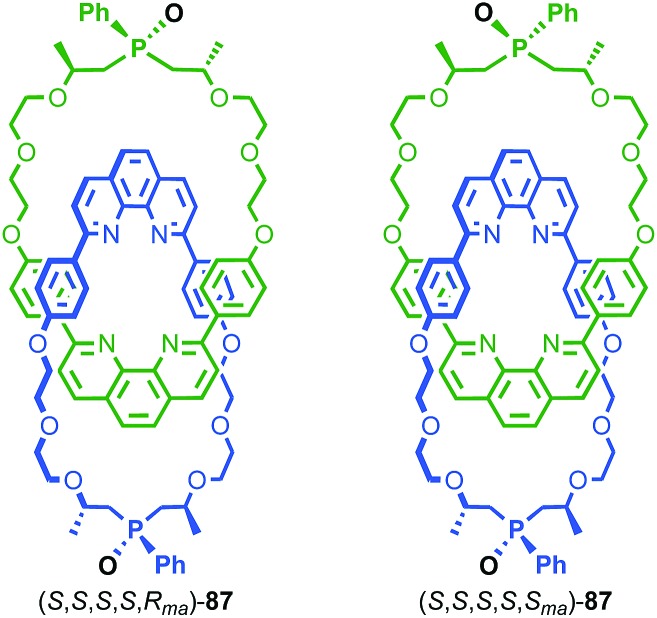
Marinetti's mechanically axial chiral catenanes **87**. (*S*) stereodescriptor refers to all covalent stereocentres in macrocycle framework.^105^ Highest priority off-ring plane atoms in each component are highlighted in black.

In 2006 Marinetti and co-workers synthesised catenane **87** which is derived from an enantiopure covalently chiral building block to give mechanical axially epimeric diastereoisomers (*S*,*S*,*S*,*S*,*R*_ma_)-**87** and (*S*,*S*,*S*,*S*,*S*_ma_)-**87** (Fig. 42).^104^ The diastereomers of **87** were separated by HPLC and characterised by ^1^H NMR. The chiral macrocycle alone does not display significant desymmetrisation of the phenanthroline moiety by ^1^H NMR, unsurprising since the stereochemical information is remote from the heterocycle. In contrast, the phenanthroline moieties in both diastereomers of catenane **87** show separate signals for many of the phenanthroline environments. This confirms that, although the immediate cause of the mechanical axial chirality, the phosphine oxide moiety, is remote from the phenanthroline unit, the mechanical axial chirality of **87** is well expressed throughout the whole molecule.

**Fig. 43:**
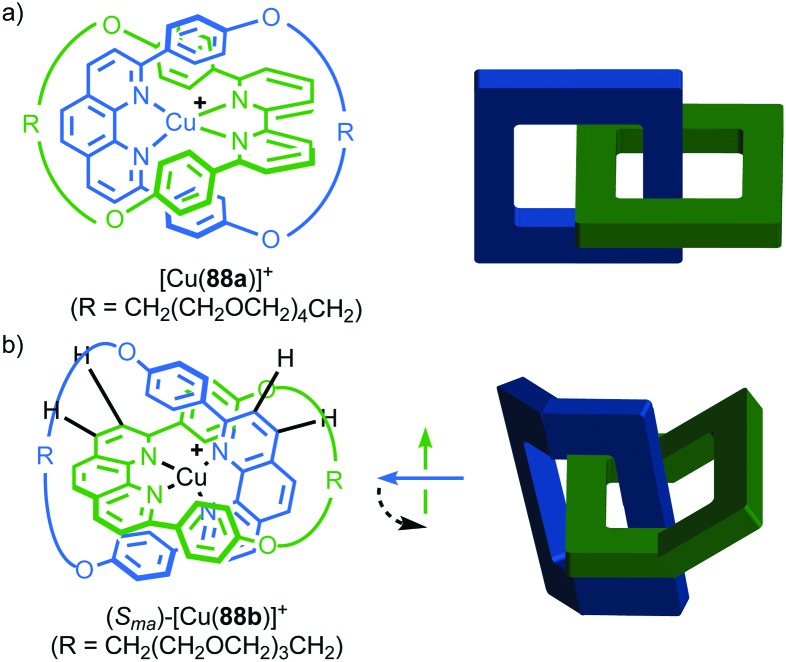
(a) Sauvage's achiral catenane **88a**^107^ and (b) conformationally mechanically axially chiral catenane **88b** with schematic representations highlighting their symmetry properties.^106^ Counter ions omitted for clarity.

Finally, although substitution of a tetrahedral centre is the dominant structural feature leading to facial asymmetry in the macrocycles of axially chiral catenanes, indeed the only one reported to date, it should be noted that any combination of *C*_*n*v_ macrocycles in which the principle axis is perpendicular to the ring will result in mechanical axial chirality. For example, *C*_2v_ symmetry can be achieved by bending rings such that they have a concave and a convex face. Recognising this, it is possible to categorise catenane **88b** (Fig. 43b), reported by Sauvage and co-workers,^105^ as a unique example of a mechanically axially chiral molecule that owes its chirality to the restricted conformation of the macrocycles.

Catenane **88a**, reported by Sauvage and co-workers in their ground-breaking development of the passive metal templated synthesis of catenanes,^106^ is composed of two macrocycles with *C*_2v_ symmetry in which the principle axis is in the plane of the macrocycle (Fig. 43a). This symmetry is maintained in catenane **88a** as the rings sit perpendicular to one another with an average planar conformation and so catenane **88a** does not meet the requirements set out above for mechanical topological or axial chirality and is achiral. Catenane **88b** is composed of smaller rings with the same symmetry (Fig. 43b). However, ^1^H NMR analysis of catenane **88b** reveals many diastereotopic resonances and in the presence of chiral shift agent (*R*)-trifluoro-methyl anthryl ethanol, the ^1^H NMR resonances of **88b** split further, confirming that **88b** is chiral, at least on the ^1^H NMR timescale.

The origin of the molecular asymmetry of **88b** is the restricted conformations of the macrocycles; due to the shorter linker unit, the chain cannot encircle the phenanthroline moiety and is thus displaced to one side or the other. This leads to bent macrocycle conformations in catenane **88b** with no stable fully planar conformations available and thus the faces of each macrocycle are non-equivalent; in the catenane, the two rings adopt a *C*_1v_ (*C*_s_) conformation resulting in the emergence of mechanical axial chirality. Because the enantiomers were not separated, it is not clear from the original report if catenane **88b** is atropisomeric under all conditions (*i.e.* the barrier to conformational racemisation is greater than that of bond breaking) or if the system is dynamic but the enantiomers exchange slowly on the ^1^H NMR timescale.

The method of assigning the absolute stereochemistry of **88b** is unclear as it does not obviously meet the structural requirements laid out above. We tentatively propose that an obvious modification would be to consider the convex face as being of higher priority with the vector required for stereochemical assignment pointing from convex to concave. This leads to the stereochemical assignment shown for **88b**.

#### Conclusions

A limited number of mechanically axially chiral catenanes have been reported but, beyond some preliminary spectroscopic studies, nothing is known about their properties and potential applications. That said, the selective display of substituents on the macrocycle faces, a key feature of the stereogenic unit, seems well suited for applications in catalysis. For example, Marinetti's catenane **87**, seems ideally placed to be explored as a chiral chelating phosphine ligand. As methods for the synthesis of catenanes improve hopefully these molecules will once again attract interest for potential applications.

## Co-conformational stereogenic units

The elements of conditional mechanical stereoisomerism presented in the preceding section, mechanical planar in the case of rotaxanes, mechanical axial and topological in the case of catenanes, are static properties of the molecules concerned and the absolute configuration of these molecules can be specified by considering the covalent constitution of the subcomponents in combination with the mechanical constitution of the interlocked structure.

Interlocked molecules can also display chirality even when the covalent structures of their components do not meet the symmetry requirements for conditional mechanical chirality laid out above. Specifically, the relative positions, known as co-conformations (by analogy with conformations in covalent molecules) of the covalent sub-units in the interlocked structure serve to desymmetrise one another, leading to the appearance of molecular chirality. For this reason, we propose that these are examples of “co-conformational” chirality by analogy with conformational chirality,^4^ where enantiomers are exchanged by single bond rotations.

In some cases, the distinction we have chosen to make between conditional mechanical and co-conformational stereochemistry is at odds with previous discussions, and we will highlight these disagreements where appropriate. However, we are of the opinion that this distinction is useful in categorising the molecules under discussion and their behaviour, particularly with respect to the dynamics of the stereogenic unit; as with conformational enantiomers, the co-conformational stereogenic units we have identified can either be dynamic or static on the timescale of a given experiment. In the former case the barrier to co-conformational motion is low enough that the system can adopt enantiomeric arrangements through shuttling or pirouetting, in the case of rotaxanes and catenanes respectively. In the latter situation, the barrier to relative motion is effectively infinite on the timescale of the measurement, typically due to steric hindrance. In this sense, co-conformational chirality bears a striking resemblance to atropisomerism in covalent chemistry.

In contrast, no conformational (with the exception of catenane **88b**, which combines elements of conformational and mechanical stereochemistry) or relative movement of the covalent subcomponents (without separating them) can exchange conditional mechanical stereoisomers. It is for this reason that we consider the conditional mechanical and co-conformational stereogenic units to be distinct stereochemical phenomena.

### Co-conformational covalent point chirality

#### Description of the stereogenic element

**Fig. 44:**
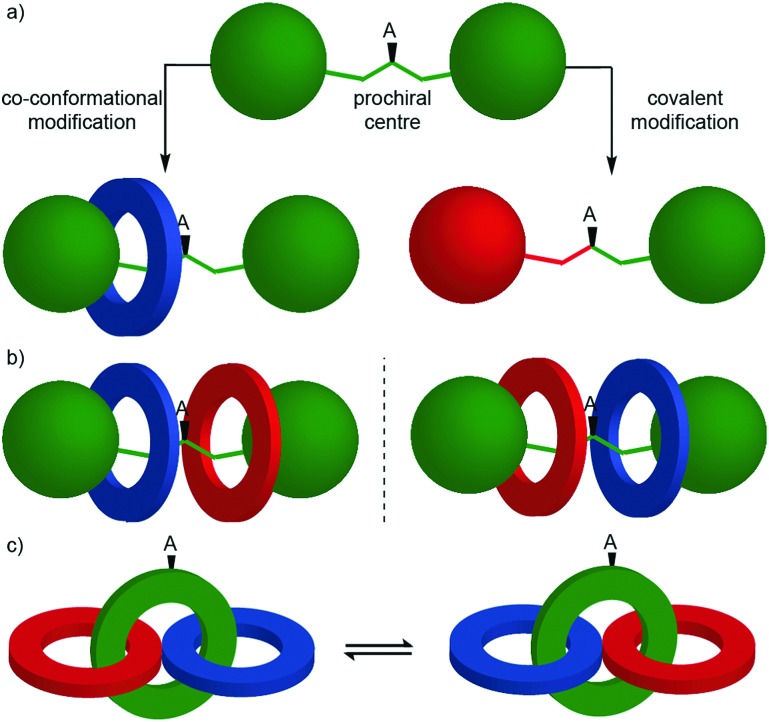
Co-conformational covalent point chiral molecules. (a) Relationship between a prochiral centre co-conformational covalent point chirality and desymmetrisation by covalent modification. (b) Co-conformational covalent point chiral [3]rotaxane. (c) Co-conformational covalent point chiral [3]catenane whose enantiomers are exchanged by pirouetting.

The best known chiral stereogenic element is a tetrahedral carbon atom with four inequivalent ligands. Such stereocentres can be considered to arise by the desymmetrisation of a prochiral unit by modification of one of the enantiotopic substituents (Fig. 44a). Classically, this is achieved by covalent modification of the enantiotopic substituents, but conceptually it is trivial to imagine this desymmetrisation occurring by localising a macrocycle over one of two prochiral regions of the axle of a rotaxane (Fig. 44b) or one macrocycle of a catenane (Fig. 44c). Considering the symmetry properties of the system, the position of a macrocycle on either side of a prochiral centre serves to remove the mirror symmetry of other covalent subcomponent which has *C*_s_ (*C*_1h_) symmetry. In this manner, the mechanical bond can give rise to chirality that is both fundamentally a covalent property of the molecule (a prochiral covalent centre is desymmetrised) and co-conformational (relative movement of the components inverts the absolute covalent stereochemistry); when co-conformational motion is unrestricted, exchange between enantiomers can be considered to occur by an achiral co-conformation in which the desymmetrising macrocycle is localised perfectly on the mirror plane of the *C*_s_ component, and so the molecule has at least one achiral presentation. However, as with atropisomers, when co-conformational motion is prevented, typically by a large group to sterically block the passage of the macrocycle, this leads to separable co-conformationally chiral enantiomers.

We should note that Leigh and co-workers proposed the term “mechanical point/centrally chiral” to describe this stereogenic unit in the case of [2]rotaxanes,^107^ in order to emphasise the role the mechanical bond plays in the appearance of molecular chirality. We have preferred to categorise these molecules as co-conformationally covalent point/centrally chiral as this serves to emphasise the key role covalent stereochemistry and co-conformation play in the stereogenic unit. This revised taxonomy also serves to link the dynamic behaviour of this stereogenic unit with the other classes of co-conformational stereochemistry outlined below. Their method of stereochemical assignment is also strongly linked.

Finally, although all examples of co-conformational covalent point chiral molecules disclosed to date are [2]rotaxanes, the co-conformational covalent point stereogenic element also arises in higher order structures. Most obviously, a hetero[3]rotaxane with a *C*_s_ axle can exist as two stereoisomers depending on the order of macrocycles along the axle (Fig. 44b). Since macrocycles cannot typically move past one another on an axle,^108^ these co-conformational enantiomers are unlikely to be in exchange on any reasonable time frame. In contrast, although a hetero[3]catenane (Fig. 44c) can also exist as a pair of enantiomers, these are exchangeable by pirouetting unless the co-conformational motion is restricted in some way.

#### Assignment of absolute stereochemistry

Leigh and co-workers proposed an intuitive and simple method for the assignment of co-conformational covalently point chiral structures in which the macrocycle is considered a substituent of the region of the axle that it occupies; effectively the covalent structure is desymmetrised by a “ghost atom” that represents the interlocked substituent.^108^ Here we propose a slight modification of this approach to generalise it to molecules containing multiple stereochemically relevant interlocked components. Thus, to assign the stereochemistry of co-conformationally covalent chiral molecules we propose the following:

(i) Where more than one stereochemically relevant interlocked component is present (*e.g.*Fig. 44b and c) and the system is stereochemically dynamic, the subunits are considered evenly distributed either side of the mirror plane of the *C*_s_ component that is desymmetrised by the mechanical bond. When the number of macrocycles is odd, the central macrocycle is considered localised on the *C*_s_ mirror plane and thus is non-stereogenic. Where the system is static (*i.e.* the components are trapped in a preferred co-conformation) or where the stereochemical assignment of a specific co-conformation is of interest (*e.g.* rotaxane **89**), they are considered to be on the appropriate side of the *C*_s_ mirror plane.

(ii) Each stereochemically relevant macrocycle is assigned a relative priority by comparing their highest priority constituent atoms according the CIP rules. If two rings are otherwise identical but have different mechanical or covalent absolute stereochemistry, *R* takes priority over *S*.

(iii) The interlocked components are then added in order of priority, as “ghost” substituents of the atoms on the side of the mirror plane they were assigned to in (i) until a stereochemical assignment of the desymmetrised component is possible using the established CIP method.

This approach is essentially a generalisation of that suggested by Leigh in the case of [2]rotaxanes to complex structures containing multiple interlocked components. It also has the advantage that it can be extended consistently to the other co-conformational stereogenic elements in the following sections.

#### Synthesis, properties and applications

**Fig. 45:**
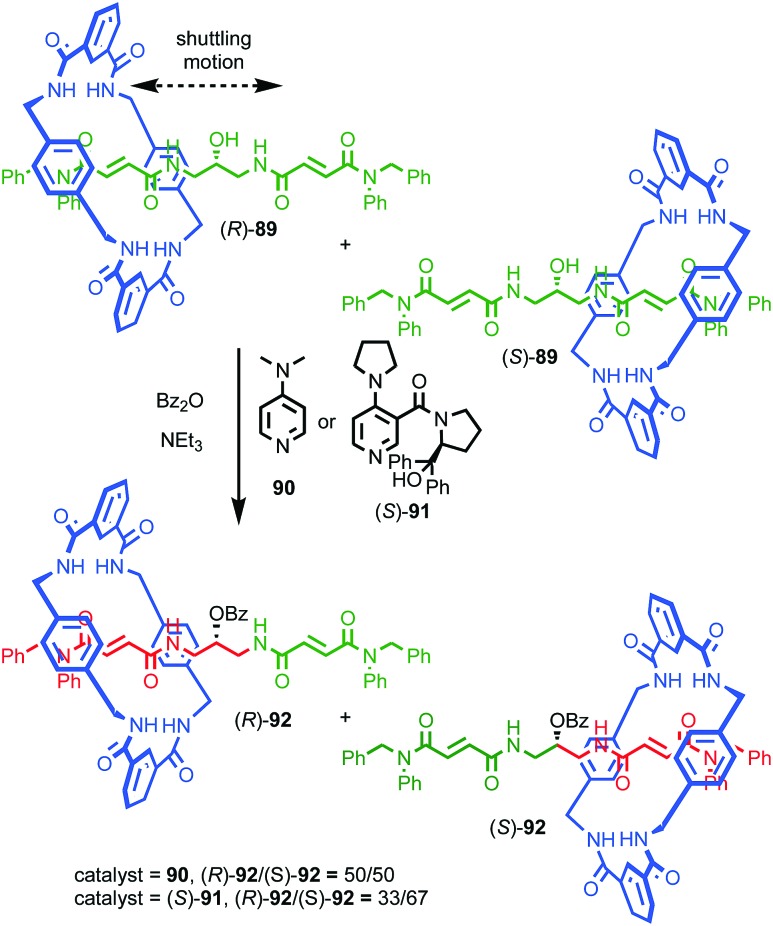
Leigh's co-conformationally covalent point chiral information ratchet.^108^

Leigh and co-workers have studied co-conformational covalent point chirality in the context of molecular motors and catalysis. Rotaxane **89** exists as a racemic mixture of enantiomers that are exchanged by shuttling of the macrocycle (Fig. 45).^108^,^109^ This co-conformational racemisation process can be halted by benzoylation of the hydroxyl moiety in the presence of DMAP (**90**) to produce a racemic mixture of (*R*)-**92** and (*S*)-**92**, as demonstrated by CSP-HPLC. When (*R*)-**91** is used in place of DMAP in the benzoylation of **89**, the product is formed in which the macrocycle is selectively trapped to give an excess of (*R*)-**92**. Conversely, (*S*)-**91** produces an excess of (*S*)-**92**. Thus, Leigh and co-workers demonstrated that it is possible to distinguish between co-conformationally point chiral enantiomers using a chiral catalyst. In addition to being a catalytic asymmetric synthesis of co-conformationally covalent point chiral rotaxanes, in machine terms, this is an example of an information ratchet mechanism as the position of the particle (the macrocycle) affects the rate of a process which selectively traps it in one mechanical state.^110^,^111^

**Fig. 46:**
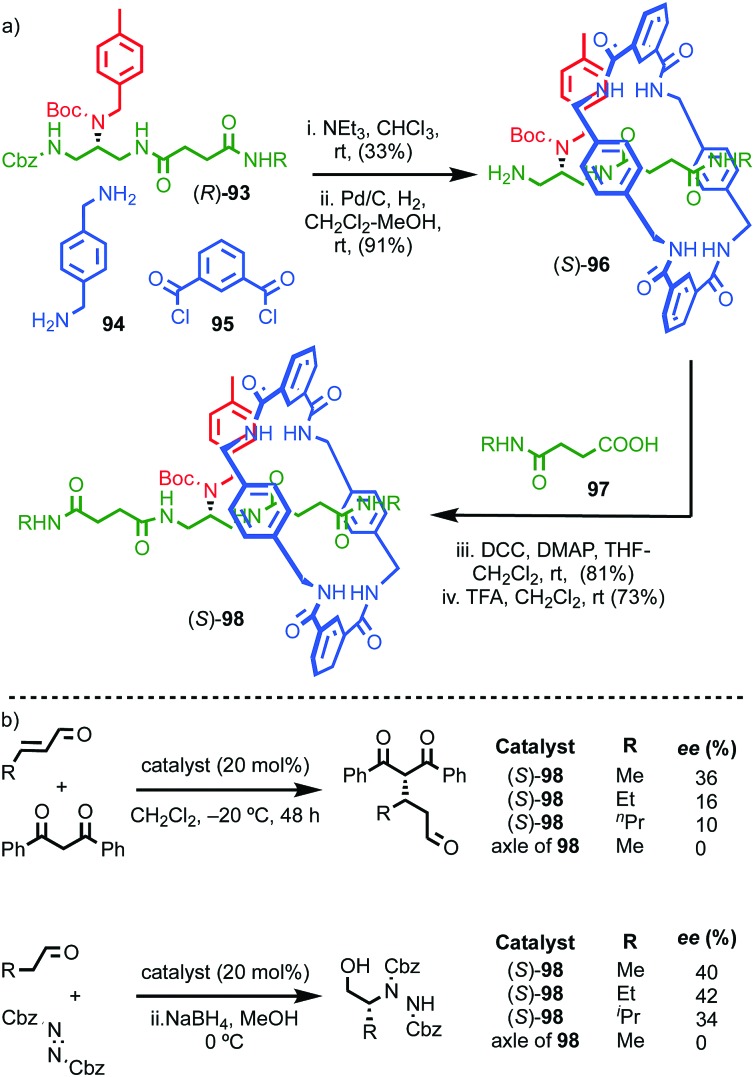
(a) Synthesis of Leigh's co-conformationally point chiral catalyst (*S*)-**98** (R = CH_2_CH(Ph)_2_). (b) Enantioselective reactions mediated by (*S*)-**98**.

Leigh and co-workers also demonstrated the potential of co-conformational chirality outside the realms of molecular machines.^112^ Rotaxane (*R*)-**98** was synthesised in 84% ee from an enantioenriched starting material (*R*)-**93** by selectively installing the macrocycle on one side of the bulky benzyl amine during the synthesis (Fig. 46a). The amine unit acts as an organocatalyst and can direct the Michael addition of activated carbon nucleophiles to a series of α-β-unsaturated aldehydes under iminium catalysis in up to 36% ee (Fig. 46b) or the α-amination of aldehydes under enamine catalysis in up to 42% ee (Fig. 46b).^113^

#### Conclusions

Co-conformationally covalently chiral interlocked molecules have not received much attention to date, although with the results from Leigh and co-workers in the context of machines and catalysis, the stage has been set for further developments. Of the forms of mechanical chirality presented here, it is arguably the most intuitive and amenable to standard synthetic methods using enantioselective catalysis or chiral pool approaches, as demonstrated by Leigh in the context of rotaxanes. Similar approaches should be applicable to catenanes. Furthermore, it is also possible to envisage other forms of co-conformational covalent chirality and these are discussed in the final section of this review.

### Co-conformationally mechanically planar chiral rotaxanes

#### Description of the stereogenic element

**Fig. 47:**
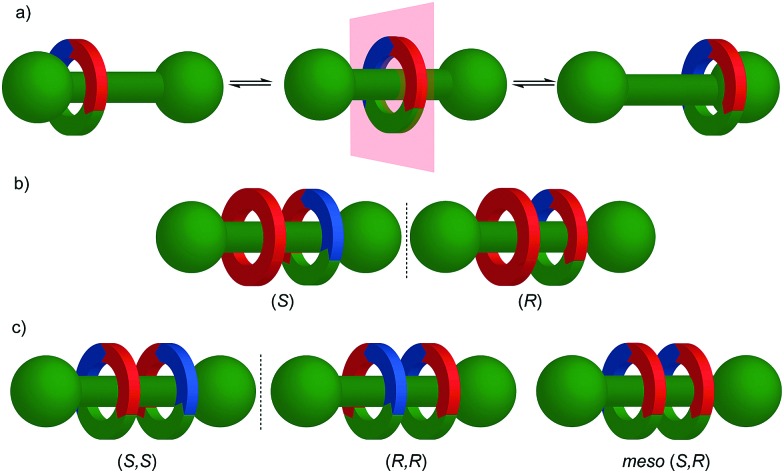
Schematic representation of (a) an achiral [2]rotaxane and its co-conformational mechanical planar chiral enantiomers, (b) the enantiomers of a co-conformational mechanical planar chiral [3]rotaxane composed of one oriented and one un-oriented ring; (c) the stereoisomers of a co-conformational mechanical planar chiral [3]rotaxane composed of two oriented rings. Arbitrary stereodescriptors shown.

An oriented *C*_*n*h_ macrocycle combined with an axle in which the two ends are identical (*C*_2v_ or *D*_∞h_), is on average achiral as the system possesses a mirror plane parallel with the macrocycle when it is positioned in the centre of the axle (Fig. 47a). However, if the macrocycle is displaced either side of the central mirror plane of the axle component, the assembly becomes desymmetrised producing enantiomers. Such [2]rotaxanes can be dynamic or, if a blocking group is introduced in the centre of the axle to prevent the shuttling of the macrocycle, static. This stereogenic unit as similar properties to both mechanical planar chiral rotaxanes and co-conformational covalent point chiral systems and so we propose they be termed co-conformational mechanically planar chiral to emphasise that co-conformation determines the appearance of the stereogenic element and that this is a characteristic of the mechanical bond as a whole rather than a specific covalent moiety; essentially the position of the macrocycle desymmetrises the axle component which, combined with the *C*_*n*h_ symmetric macrocycle, leads to mechanical planar chirality.

Similar stereoisomerism can manifest in [*n*]rotaxanes in which at least one of the macrocycles is oriented (Fig. 47b and c) and, given that typically macrocycles in interlocked structures are not able to pass one another, this form of stereoisomerism is almost always fixed. Thus, a [3]rotaxane with a centrosymmetric axle, one oriented and one non-oriented ring exists as a separable pair of enantiomers (Fig. 47b). Furthermore, a [3]rotaxane in which both macrocycles are oriented can form as two diastereomers, one of which exists as a pair of enantiomers and the other is *meso* (Fig. 47c).

#### Assignment of absolute stereochemistry

No explicit rules have been proposed for the stereochemical assignment of the co-conformationally mechanically planar stereogenic unit, although Vögtle and co-workers have passed some comment in the specific case of homo[3]rotaxanes.^114^ We propose this can be achieved simply by combining the logic presented above for the assignment of axle priorities in the co-conformational covalent point stereogenic unit, with the accepted approach for the assignment of mechanically planar chiral rotaxanes. Thus, for each oriented *C*_*n*h_ macrocycle in turn:

(i) Determine orientation of the macrocycle using the method presented for conditional mechanical planar stereochemistry.

(ii) Where more than one stereochemically relevant interlocked component is present (*e.g.*Fig. 47c) and the system is stereochemically dynamic, the subunits are considered evenly distributed either side of the central mirror plane perpendicular to the axle component. When the number of macrocycles is odd, the central macrocycle is considered localised on this mirror plane. Where the system is static (*i.e.* the components are trapped in a preferred co-conformation) or where the stereochemical assignment of a specific co-conformation is of interest (*e.g.* rotaxane **100**, Fig. 49) they are considered to be localised on the appropriate side of the central mirror plane of the axle.

(iii) Each stereochemically relevant macrocycle is assigned a relative priority by comparing their highest priority constituent atoms according the CIP rules. If two rings are otherwise identical but have different mechanical or covalent absolute stereochemistry, *R* takes priority over *S*. By convention the oriented macrocycle under consideration is given the lowest priority.

(iv) The macrocycles are then added in order of priority, as “ghost” substituents of the highest priority atoms on the side of the mirror plane they were assigned to in (ii) until the orientation of the axle component can be determined using the standard approach for the mechanical planar chiral stereogenic unit.

(v) Using the orientation of the axle determined in (iv), the absolute stereochemistry of the ring under consideration is assigned as for mechanically planar chiral rotaxanes.

**Fig. 48:**
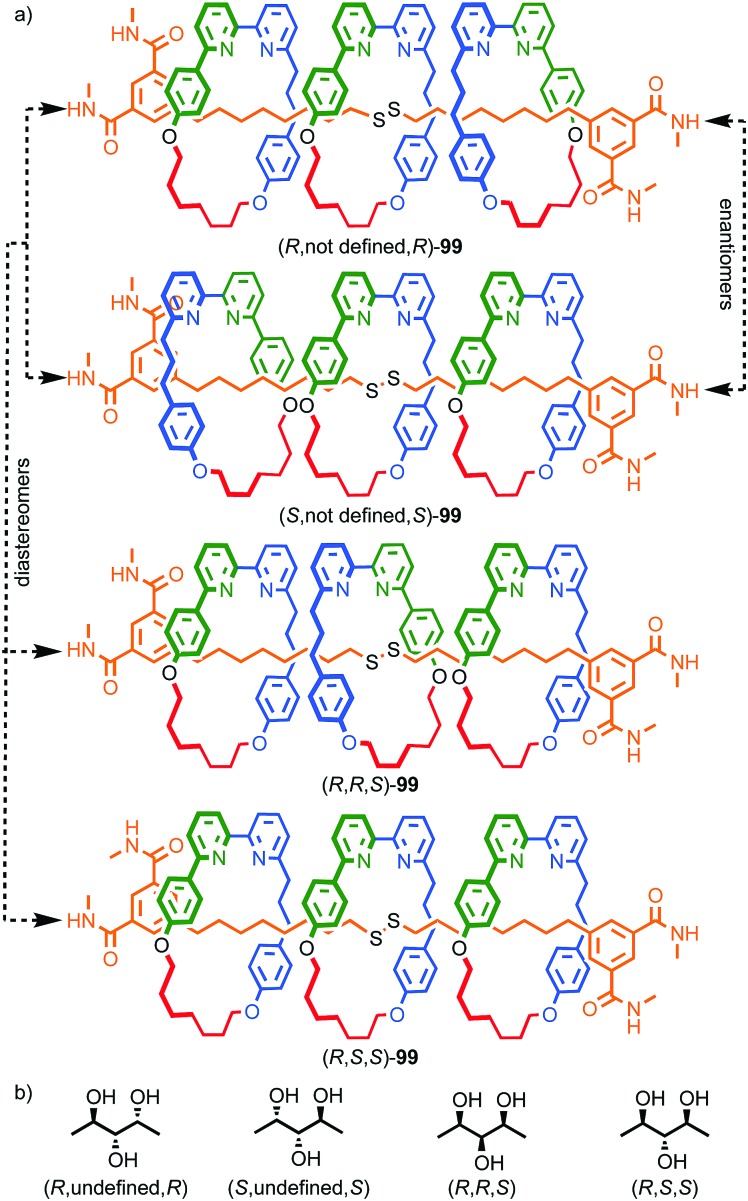
(a) Stereochemical assignment of hypothetical co-conformational mechanical planar chiral [4]rotaxane **99**. In all cases the highest priority atom of the macrocycle is the emboldened “O” in black. For *meso* diastereomers (*R*,*R*,*S*)-**99** and (*R*,*S*,*S*)-**99** the stereochemistry of the central macrocycle is determined by assigning the *R* configured macrocycle on the left the highest CIP priority. (b) Stereoisomers of 2,3,5-trihydroxypentane demonstrating similar stereochemical properties to [4]rotaxane **99**. Highest priority atoms in each component are highlighted in black.

We have exemplified our proposed approach in the case of dynamic [2]rotaxane **100** reported by Saito and co-workers.^115^ However, given that molecules exhibiting the full complexity possible in such systems have not been reported, we have exemplified the assignment of stereochemistry for the different diastereomers of a dynamic [4]rotaxane using hypothetical structure **99** (Fig. 48). This allows to demonstrate two slightly unusual properties of this stereogenic element: (1) the orientation of the axle is not fixed but varies depending on the macrocycle under consideration; (2) although the central macrocycle is considered as occupying the axle mirror plane and plays no role in assigning the absolute stereochemistry of the other rings, it is still potentially stereogenic and must be considered in order to achieve a full stereochemical assignment in some cases (*cf.* the central stereocentre in 2,3,4-trihydroxypentane which results in two *meso* diastereomers with opposite configuration of the central hydroxyl; Fig. 48b).

#### Synthesis and properties

**Fig. 49:**
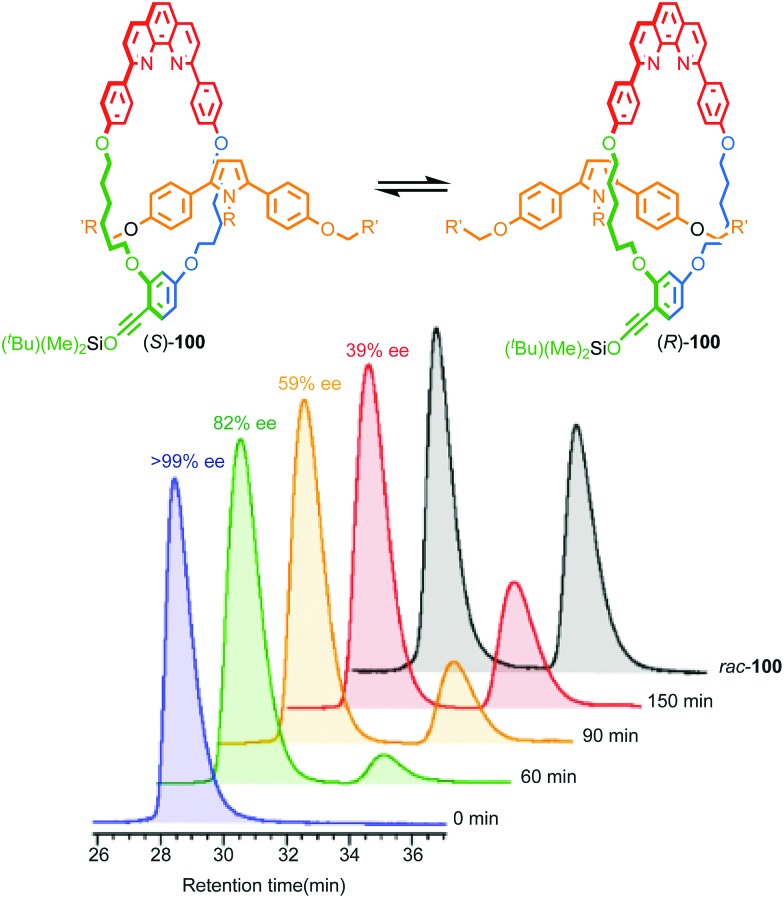
Saito and co-workers co-conformationally mechanically planar chiral rotaxane **100** as a stereodynamic probe for ring shuttling.^116^ CSP-HPLC analysis of the racemisation of enantiopure **100** in (CHCl_2_)_2_ at 413 K. R = 4-C_6_H_4_(cyclohexyl). R′ = (CH_2_)_5_C(4-C_6_H_4_(4-C_6_H_4_(cyclohexyl)))_3_. Highest priority atoms in each component are highlighted in black. Reprinted from ref. 116. Copyright 2016 American Chemical Society.

The explicit study of co-conformationally mechanically planar chiral [2]rotaxanes is extremely rare. Although Takata and co-workers have discussed this form of molecular asymmetry,^116^ the only study of the chirality of such molecules was recently reported by Saito and co-workers (Fig. 49).^116^ Rotaxane **100** exists as a pair of co-conformationally planar chiral enantiomers with the hand of the molecule exchanged by shuttling of the macrocycle between the compartments over the bulky *N*-substituent of the pyrrole moiety. The shuttling of the macrocycle was slow enough to allow the separation of the enantiomers of **100** by CSP-HPLC, which were confirmed to display mirror image CD spectra, although the absolute stereochemistry of the enantiomers was not assigned. The authors used the relationship between shuttling and racemisation to determine the activation parameters for the shuttling of the macrocycle over the pyrrole, something which they had been unable to do in a related achiral system as the process was too slow to monitor by ^1^H NMR.^117^

**Fig. 50:**
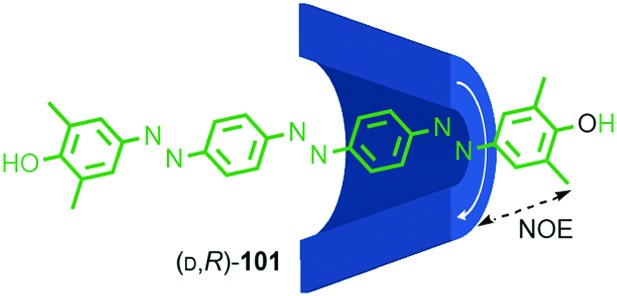
Preferred co-conformation of Anderson's α-cyclodextrin [2]rotaxane (d,*R*)-**101**. Highest priority atoms in the axle component are highlighted in black. The orientation of the macrocycle is indicated by a white arrow.

Although Saito's example is the only explicit study of co-conformational mechanical planar chirality in a [2]rotaxane that we are aware of, as in the case of mechanically planar chiral rotaxanes, many examples have been disclosed, although not commented upon, in the context of cyclodextrin-based interlocked molecules. In these examples, the interplay between the co-conformational mechanical planar stereogenic unit and the covalent stereochemistry of the sugar moieties co-conformational diastereomers. This phenomenon is well exemplified by rotaxane **101** which was isolated by Anderson and co-workers (Fig. 50);^118^^1^H NMR studies and NOE analysis revealed that the narrow end of the macrocycle is localised over the stopper unit, demonstrating significant diastereoselectivity in the formation of a single co-conformational mechanical diastereomer which can be assigned as (d,*R*)-**101**.

**Fig. 51:**
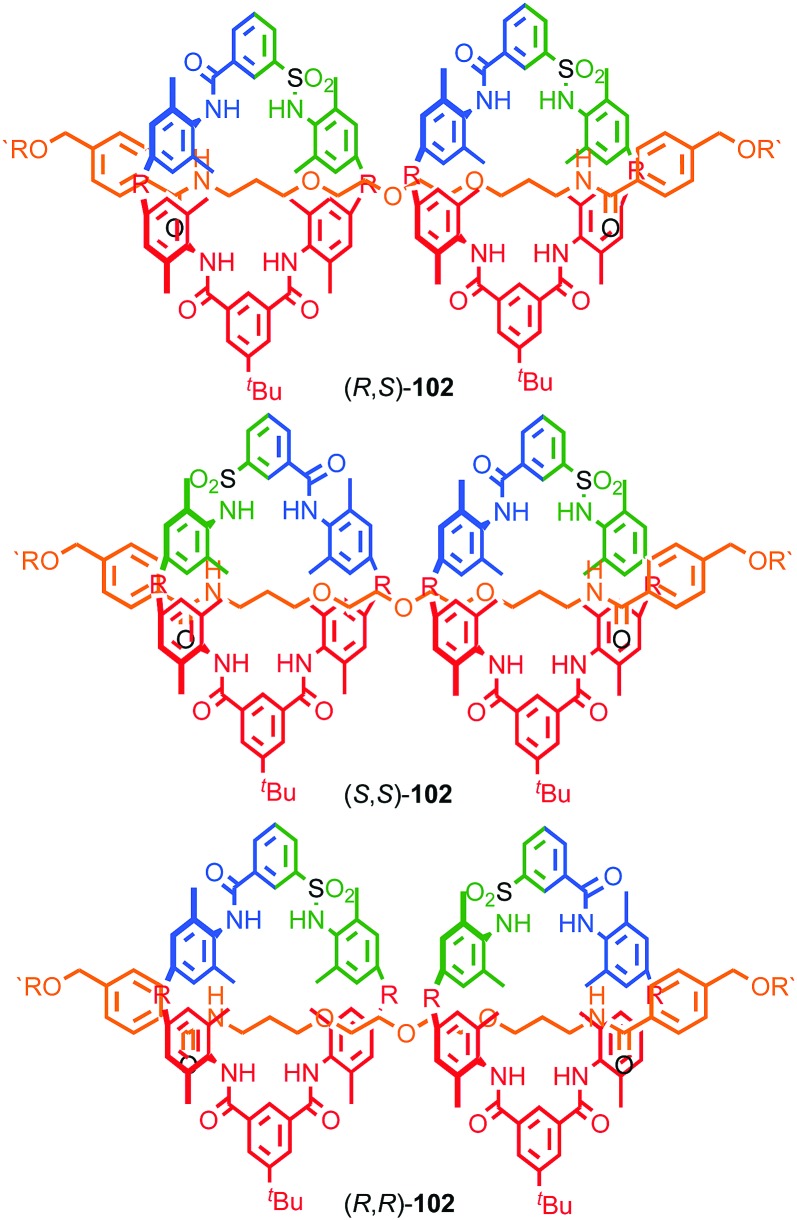
Stereoisomers of Vögtle's co-conformationally mechanically planar chiral [3]rotaxane **102**.^115^ R = 1,1-cyclohexyl, R′ = –C_6_H_4_–Tr. Highest priority atoms in each component are highlighted in black.

Surprisingly, given their higher structural complexity, studies of co-conformational chirality in [*n*]rotaxanes appeared relatively early in the exploration of mechanical chirality by Vögtle and co-workers. The authors synthesised [3]rotaxanes **102** based on two oriented macrocycles on a centrosymmetric axle (Fig. 51).^115^ [3]Rotaxane **102** can exist as two diastereomers, one of which exists as a pair of co-conformational mechanical planar chiral enantiomers and other of which is *meso*. The authors separated the stereoisomers by CSP-HPLC and confirmed that (+)-**102** and (–)-**102** display opposite Cotton effects while *meso* compound **102** is CD-silent. Vögtle and co-workers proposed that the stereochemistry associated with each oriented macrocycle could be assigned by considering the orientation of the rings with respect to a vector pointing from one macrocycle to the other. Although this is satisfactory in the case of [3]rotaxanes, in more complex [*n*]rotaxanes (*e.g.***99**) it is not clear how this rule could be extended. Using the approach developed above, the absolute stereochemistry of the stereoisomers of **102** are assigned as shown. Vögtle and co-workers later reported chiral [3]rotaxane derivatives in which the macrocycles are linked by a covalent bridge which they christened “Bonnanes”.^119^

**Fig. 52:**
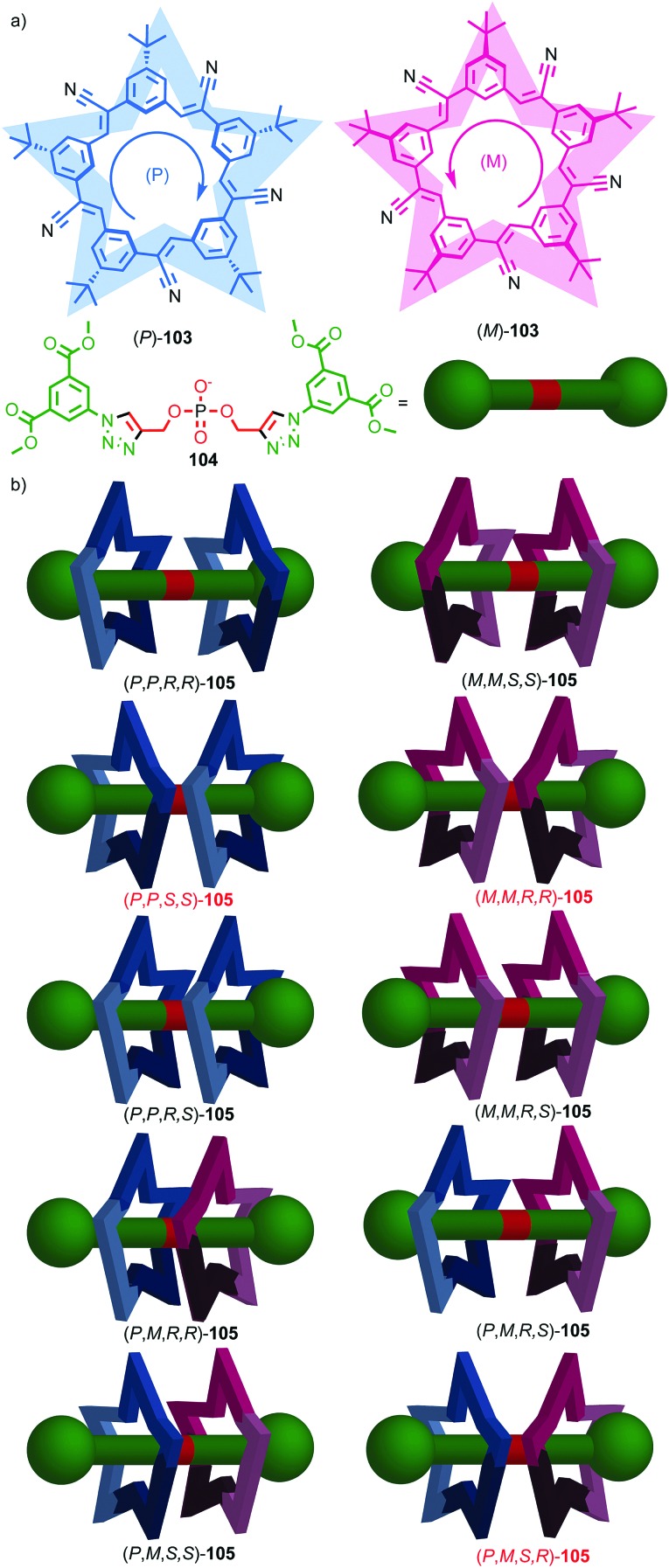
(a) Flood's cyanostar macrocycle **103** and its enantiomeric *P* and *M* bowl conformations. (b) Schematic representation of the possible stereoisomers of [3]rotaxane **105** formed when **103** encircles phosphate axle **104**.^121^ The observed (*P*,*P*,*S,S*), (*M*,*M*,*R,R*) and (*P*,*M,S,R*) stereoisomers are highlighted in red. Counter ions omitted for clarity.

Since Vögtle and co-workers first reported [3]rotaxane **102**, several examples of similar [3]rotaxanes have been reported, although the diastereoisomers are not typically separated. Flood and co-workers reported an interesting example based on their cyanostar macrocycle, **103**, which can be produced in excellent yield from simple starting materials, and has the potential to express both mechanical planar and covalent planar stereochemistry simultaneously (Fig. 52).^120^

In isolation, **103** adopts enantiomeric cone-shaped conformers in which each aromatic moiety can be considered to act as a planar stereogenic unit. Cyanostar **103** was found to form a 2 : 1 complex with a number of lipophilic anions, in which the anion is sandwiched between the two macrocycles. This observation was extended to the synthesis of [3]rotaxane **105** in which two macrocycles encircle axle **104**. As in Vögtle's original study, if the relative orientation of the macrocycles alone is considered, rotaxane **105** can exist as a pair of diastereomers, one of which is mechanically planar chiral and the other a *meso*-form. Taking into account the planar chiral conformations of the cyanostar rings, a total of 10 stereoisomers are possible. In reality, the only conformation that is observed is that in which the narrow rim of the macrocycle is oriented towards the phosphate unit, leading to a statistical mixture of (*P*,*P*,*S*,*S*)-, (*M*,*M*,*R*,*R*)- and (*P*,*M*,*S*,*R*)-**105**.

**Fig. 53:**
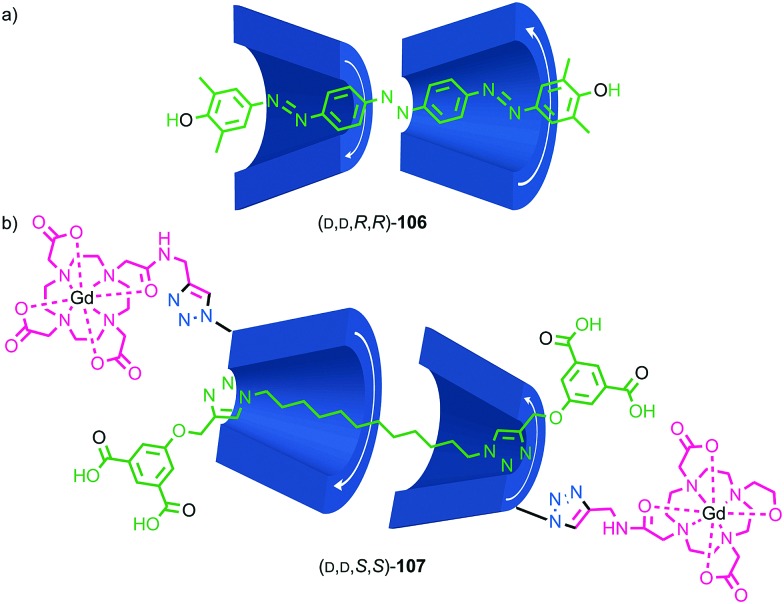
(a) Anderson's cyclodextrin [3]rotaxane **106** that forms as the head to head (d,d,*R*,*R*) isomer (d refers to stereochemistry of all glucose units in each macrocycle).122*b* (b) Hasenknopf and Vives cyclodextrin [3]rotaxane **107** for MRI imaging that forms as the tail to tail (d,d,*S*,*S*) diastereomer (stereochemistry shown is for Gd as the highest priority atom in the macrocycle; d refers to stereochemistry of all glucose units in each macrocycle).122*c* Counter ions omitted for clarity. Highest priority atoms in the axle components are highlighted in black. The orientations of the cyclodextrin rings (in the case of **107**, taking into account that Gd is the highest priority atom) are shown by a white arrow.

Once again, probably the most common mechanically planar chiral [3]rotaxanes of this type are based on cyclodextrin macrocycles.^121^ Indeed, it is relatively common for cyclodextrins to arrange themselves on a guest diastereoselectively as the pattern of hydrogen bond donors and acceptors is different on the two rims of the cone shaped macrocycle. This threading can happen in a head-to-head fashion, tail-to-tail or head-to-tail fashion, the latter of which corresponds to the co-conformational mechanically planar chiral *meso*-form when only the relative orientation of the macrocycles is considered. In the synthesis of azo-dye rotaxane **106**, Anderson and co-workers observed the stereoselective formation of the head-to-head isomer corresponding to the enantiopure (d,d,*R*,*R*) diastereoisomer (Fig. 53a).122*b* More recently, Hasenknopf, Vives and co-workers reported the stereoselective synthesis of the tail-to-tail (d,d,*S*,*S*) isomer of rotaxane **107** during the synthesis of rotaxanes as a platform for MRI imaging applications.122*c*

**Fig. 54:**
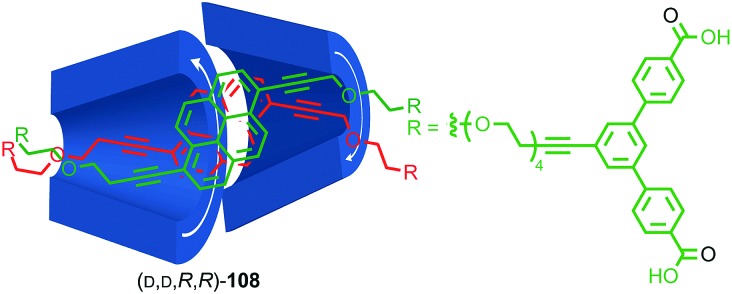
Inouyes CPL active rotaxane (d,d,*R*,*R*)-**108** (d refers to stereochemistry of all glucose units in each macrocycle).^124^ Highest priority atoms in the axle components are highlighted in black. Orientation of the cyclodextrin rings shown by a white arrow.

The discussion presented has focussed on [*n*]rotaxanes in which each macrocycle is singly threaded. Co-conformational mechanical planar chirality can also appear in more complex multiply threaded structures but these are extremely rare; indeed very few examples of multiply threaded rotaxanes have been reported in any context.^122^ Recently, Inouye and co-workers demonstrated that it is possible to use the mechanical bond to impose a chiral environment on the excited state of a molecule leading to circularly polarised luminescence (Fig. 54).^123^ [4]Rotaxane **108** was synthesised by stoppering a doubly threaded inclusion complex based on two tail-to-tail γ-cyclodextrin macrocycles leading selectivity to (d,d,*R*,*R*)-**108**. Investigation of **108** by CD, combined with molecular modelling, suggested that the pyrene units of **108** are arranged in a twisted manner imposed by the chirality of the assembly. The enforced stacking of the pyrene moieties results in an emission from an excimer state, unlike the simple inclusion complex which displayed concentration dependent emission and mixture of monomer and excimer fluorescence. The authors demonstrated that the emission of rotaxane (d,d,*R*,*R*)-**108** is circularly polarised, producing an excess of left-handed light, as a result of the chiral environment of the mechanical bond.

#### Conclusions

Because rotaxane **108** contains both co-conformational mechanical planar and covalent point stereogenic elements, it is not clear what role the co-conformational stereogenic element plays in its behaviour. However, it is perhaps the most striking application of a molecule containing co-conformational mechanical planar chirality reported to date. As methodologies to synthesise these molecules improve it should become possible to tease out the influence of the stereogenic mechanical bond and to start to determine its potential applications. Given recent progress, this is a field ripe for expansion and exploitation.

### Co-conformational mechanical helically chiral catenanes

#### Description of the stereogenic element

**Fig. 55:**
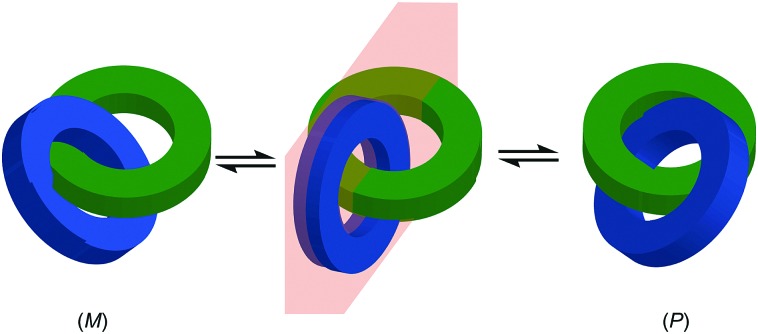
Schematic representation of rocking in a [2]catenane, leading to enantiomeric helical co-conformations.

The rocking motion of one ring relative to the other in a [2]catenane leads to desymmetrisation of the system and the emergence of chirality (Fig. 55), in a similar manner to the conformational rocking of an otherwise achiral biaryl unit. Stoddart and co-workers termed this form of chirality “mechanical helical chirality”.10*g* However, we propose that in order to emphasise the dynamic nature of co-conformational processes and that the stereogenic unit is a property of the interlocked structure as a whole, that these molecules are better considered “co-conformationally mechanically helically chiral”.

It should be noted that co-conformational mechanical helical chirality will arise in any catenane regardless of the symmetry properties of the individual covalent subcomponents, just as conformational chirality can arise in most molecules. Thus, rather than the question being “does this molecule have the correct covalent structure to display stereogenic unit *x*” as in the previous sections, all catenanes have the potential to display co-conformational mechanical helical chirality. The question is instead, “is this important to the molecule's properties on the timescale of a given experiment?”, which typically requires the molecule to have a stable helically chiral co-conformation.

#### Assignment of absolute stereochemistry

Although we were unable to identify explicit guidelines for the assignment of the co-conformational mechanical helical stereogenic unit, the method used by Stoddart and others appears to be consistent with that used in conformational stereochemistry; observing the molecule along a line perpendicular to the crossing points of the rings (Fig. 55) the stereochemistry is assigned by identifying the shortest direction of travel from the front ring to the rear ring. If the direction of travel it is clockwise the co-conformation is assigned as *P*, anticlockwise it is assigned as *M*. This method of assignment is exemplified for catenane **109**.

#### Synthesis and properties

**Fig. 56:**
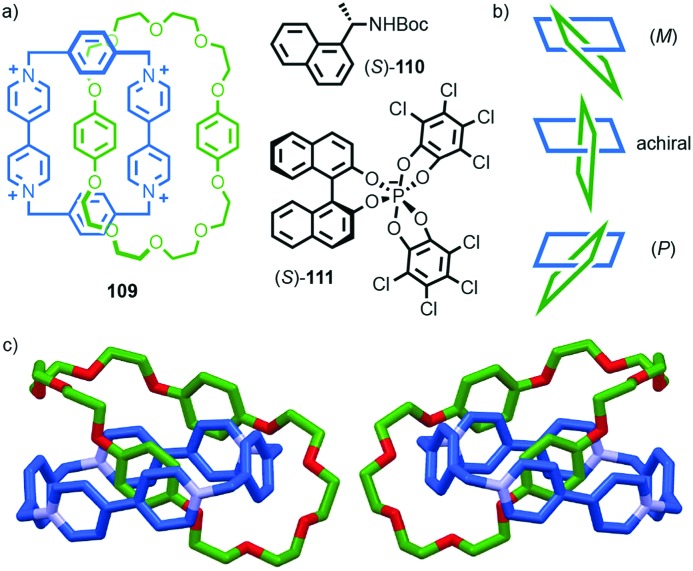
Co-conformational mechanical helical chirality in catenane **109**.^125^ Counter ions omitted for clarity.

Stoddart and co-workers first observed the appearance of such enantiomeric helical co-conformations in simple donor–acceptor catenane **109** (Fig. 56a).^124^,^125^ Single crystal X-ray analysis of **109** (Fig. 56c) demonstrates that the rings preferentially adopt a ∼45° angle relative to one another to maximise C–H···O and C–H···π interactions, resulting in a racemic mixture of helical enantiomers in the solid state. Solution state ^1^H NMR analysis suggests that this preference persists in solution; although at room temperature, the ^1^H NMR spectrum of **109** is highly symmetrical, cooling the solution to 197 K produced a spectrum in which two sets of encapsulated hydroquinone protons could be observed, consistent with the two environments found in the chiral co-conformation. Furthermore, addition of chiral shift reagents (CSRs) **110** and **111** led to splitting of enantiotopic signals, consistent with the formation of diastereomeric catenane–CSR complexes through π–π stacking (**110**) or charge–charge interactions (**111**). Furthermore, although in the case of **110** the diastereomeric signals were of equal intensity, in the presence of **111**, the diastereomeric resonances were found in a ∼2 : 1 ratio, suggesting that one diastereomeric complex is formed preferentially.

**Fig. 57:**
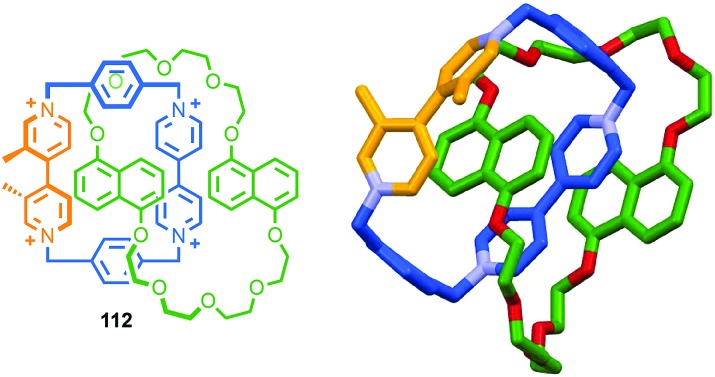
The interplay between conformational and co-conformational stereogenic elements in catenane **112**.^127^ Counter ions omitted for clarity.

The ^1^H NMR spectrum of catenane **109** in the presence of CSR **111** demonstrates that the co-conformational stereogenic element can be influenced effectively by other stereochemical information present in the system. Taking this observation a step further, Stoddart and co-workers have demonstrated that, in the presence of multiple interacting dynamic stereogenic elements within an interlocked structure, one (co)conformation can be populated preferentially.^126^ The preferred co-conformation of **112** (Fig. 57) is one in which one of the naphthyl units is encapsulated within the tetracationic cyclophane. In this arrangement, the molecule contains one conformational axial (bipicolinium unit), two conformational planar (naphthyl units) and one co-conformational mechanical helical stereogenic element leading to a total of 8 possible diastereomers that exist as pairs of enantiomers. In spite of this complexity, in the solid state (Fig. 57) a single diastereomer is observed and ^1^H NMR analysis suggests a similar situation in solution at room temperature; detailed analysis of the ^1^H NMR data reveals signals consistent with the solid-state structure^127^ and heating or cooling the sample does not lead to a loss of symmetry of coalescence of signals, suggesting the molecule is relatively static.

**Fig. 58:**
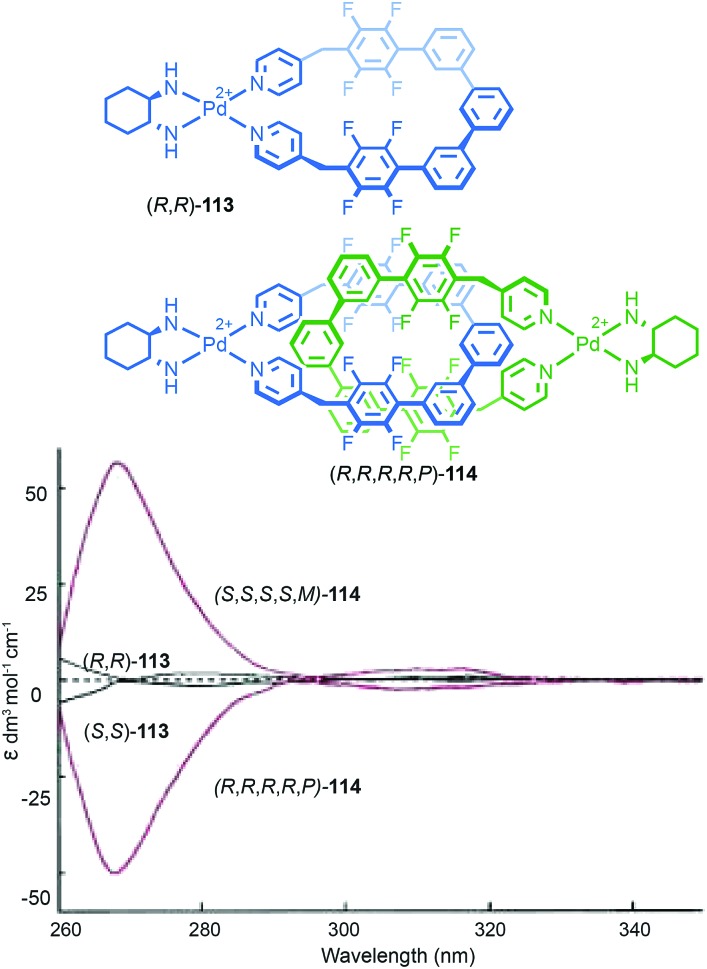
Fujita's chiral catenane **114** and a comparison of the CD spectra of macrocycle **113** and catenane **114**.^129^ Counter ions omitted for clarity. Reproduced with permission from ref. 129*a*. Copyright 2002 John Wiley and Sons.

The ability of covalent stereogenic elements to bias a source of co-conformational helical stereochemistry is shown clearly by catenane **114** reported by Fujita and co-workers (Fig. 58).^128^ Although macrocycle **113** bears a chiral diamine ligand on the Pd^II^ centres, it exhibits only extremely weak Cotton effects in its CD spectrum. This is perhaps unsurprising as the diamine moiety itself contains no significant chromophores and the Pd^II^ centre remains square planar. In contrast, catenane **114** displays a strong CD signal that originates from the flanking aromatic moieties. X-ray crystallography provided strong evidence that the difference between the CD spectra of **113** and **114** arises because catenane **114** adopts a preferred co-conformation in which the angle between the two macrocycles differs significantly from 90° due to π–π stacking between the fluorinated aromatic moieties.^129^ The chiral diamine serves to bias the co-conformational mechanical helical stereogenic unit to favour one hand and in this way the stereochemical information contained in the ligand is transmitted through the mechanical bond to the chromophoric aromatic moieties.

**Fig. 59:**
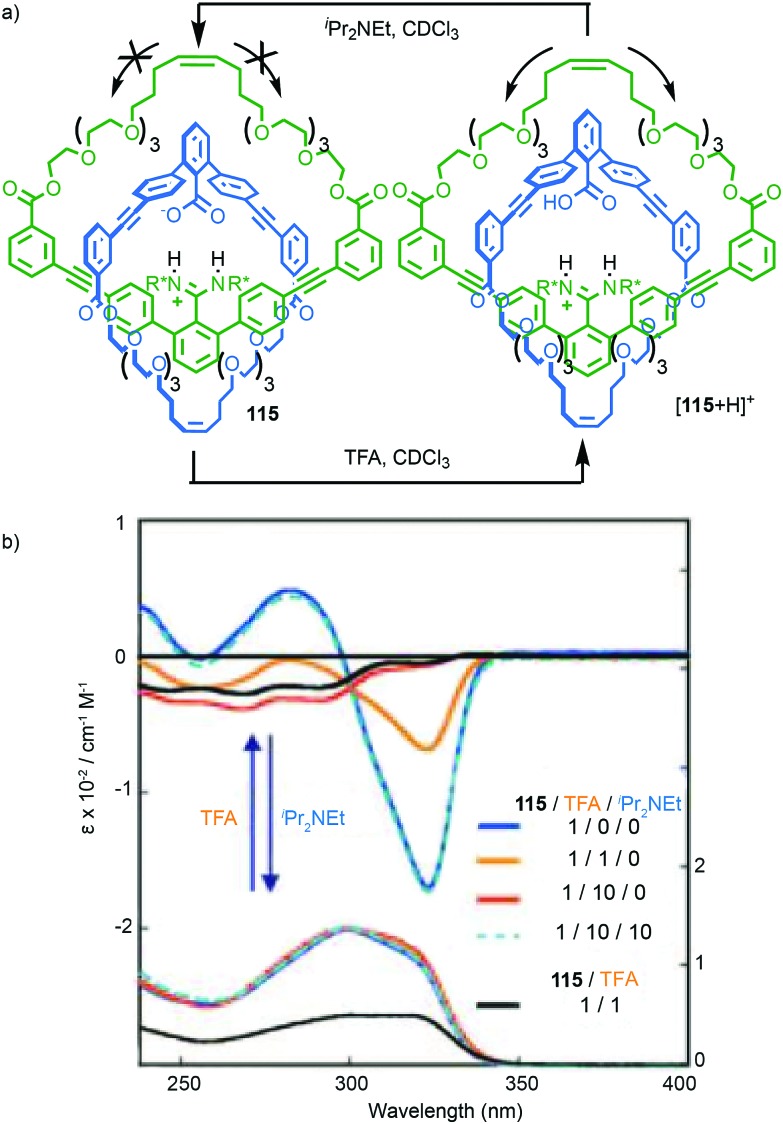
(a) Furusho and Yashima's acid and Zn^II^ responsive catenane.^131^ (b) CD and absorption spectra (CDCl_3_, 0.1 mm, *ca.* 20 °C) of the [2]catenane **115** before (blue) and after the addition of TFA 1 equiv. (orange), 10 equiv. (red), and further neutralization with 10 equiv. of iPr_2_NEt (dashed light blue). CD and absorption spectra of the TFA salt of **115** are also shown (black). Counter ions omitted for clarity. R* = (*R*)-CH(CH_3_)Ph. Reproduced with permission from ref. 130. Copyright 2010 John Wiley and Sons.

Furusho, Yashima and co-worker reported catenane **115**, assembled from chiral building blocks using a carboxylic acid–amidine salt bridge template, which produces a CD response on protonation or metal binding (Fig. 59).^130^ In the neutral state the authors propose that the salt bridge between the amidine and the carboxylic acid results in a rigid co-conformation in which the terphenyl units are canted relative to one another, corresponding to a helically chiral co-conformation that is biased by the fixed stereocentres on the amidine. In this co-conformation the molecule displays a large CD response. Protonation of the carboxylic acid moiety of **115** by TFA or binding to Zn^II^ results in a reduced CD signal that is similar to that of the non-interlocked macrocycle. The authors propose this is due to increased co-conformational freedom when the carboxylic acid is unable to form a salt bridge with the amidine, resulting in the loss of the well-defined chiral co-conformation and the CD signal associated with it.

#### Conclusions

Very few studies of the stereogenic potential of the ring rocking process in catenanes to produce co-conformational mechanical helical chiral systems have been reported, with the majority that have being those provided by Stoddart and co-workers in the context of donor–acceptor catenanes. This is perhaps unsurprising; for the co-conformational mechanical helical stereogenic unit to become important in the behaviour of a system the rocked co-conformation must have significant stability. However, it is important to be aware of these effects both for the analysis of the properties of such molecules and also in the design of chiral catenanes for new applications; the biasing of the co-conformational stereogenic element can result in significant changes in the optical properties of the assembly, as in catenanes **114** and **115**, and this dynamic element of stereochemical information could be exploited for the development of sensors and could be used to provide a well expressed chiral environment for catalysis.

### Conclusions – co-conformational stereogenic elements

Dynamic stereogenic elements that arise as a result of the mechanical bond and the relative positions of the subcomponents have already begun to receive attention and, as demonstrated by Leigh and Inouye, have the potential to lead to interesting applications. We would also like to highlight that, as with Stoddart's co-conformational mechanical helical catenanes, in such systems the interplay between multiple stereogenic elements can lead to complex and interesting behaviour. So far, very little work has been done in this area and many other combinations are available for investigation, for example a [2]rotaxane based on an oriented macrocycle and an axle containing a prochiral centre would exhibit both co-conformational mechanically planar stereogenic and co-conformational covalent point stereogenic elements which could be expected to influence the shuttling behaviour. The incorporation of co-conformational mechanical stereogenic elements can be expected to continue to contribute to the field of interlocked molecular machines, as in the case of Leigh's chemically driven information ratchet **89**.

## Unconditionally topologically chiral interlocked molecules

**Fig. 60:**
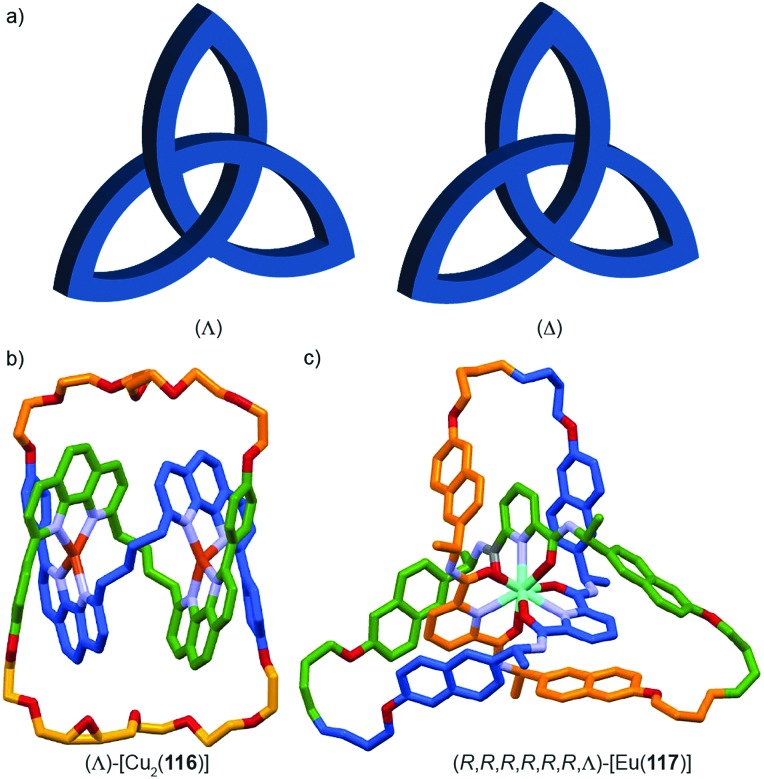
(a) Schematic representation of both enantiomers of a trefoil knot; (b) Sauvage's molecular trefoil knot **116** synthesised as a racemate using a Cu^I^-phenanthroline template;132*a* (c) Leigh's enantiopure trefoil knot **117** synthesised using a lanthanide templated approach.133*d* Counter ions omitted for clarity.

Thus far, the chirality of the molecules under discussion has been conditional either on their covalent constitution or their covalent constitution in combination with their mechanical constitution/co-conformational state. This conditionality is typically required for molecular asymmetry. However, it is possible for molecular asymmetry to arise due to a molecule's topology with no underlying conditions for the constitution or configuration of the molecular structure.^13^ The most famous example of this phenomenon is the trefoil knot (Fig. 60a), examples of which have been produced in racemic^131^ (Fig. 60b) or enantiopure form by resolution of the topological enantiomers^132^ or inclusion of covalent stereogenic units to produce separable topological epimers^133^ (Fig. 60c), and using similar template-directed approaches developed for the synthesis of interlocked molecules. Higher order knots also display topological chirality^134^ and mathematical knot theory has revealed that the majority of, but not all, knotted structures exhibit topological chirality in three-dimensional space. Knotted molecules and knot theory are extensive topics in their own right and lie outside the scope of this review. The interested reader is directed to an excellent recent review.^135^

**Fig. 61:**
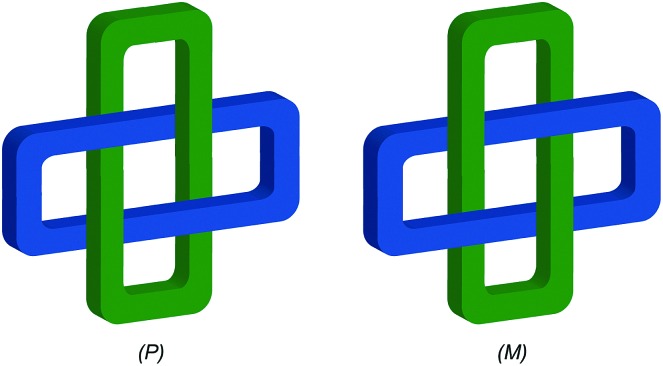
Schematic representation of both enantiomers of a Solomon link and the assignment of the molecular asymmetry.

The majority of interlocked molecules synthesised to date do not display unconditional topological chirality. Even relatively topologically complex molecules such as heterocircuit Borromean rings remain achiral without orientation of at least two of the macrocycles or the inclusion of elements of covalent chirality (Fig. 61).^136^ However, in general, as the number of crossing points between two mutually interlocked components rises, the potential for unconditional topological chirality rises. It is not practical (or perhaps even desirable) to try and categorise unconditionally topologically chiral interlocked molecules as this phenomenon arises in a variety of ways. Thus, in this section, we will provide a brief overview of the unconditionally topologically chiral interlocked molecules disclosed to date.

### Braided [2]catenanes

As discussed above, simple [2]catenanes with two crossing points do not display unconditional topological chirality. However, as the number of crossing points between two interlocked rings is increased, unconditional topological chirality emerges. The simplest interlocked molecule to display unconditional topological chirality is the Solomon link which is a braided [2]catenane with four crossing points (Fig. 61).^13^

**Fig. 62:**
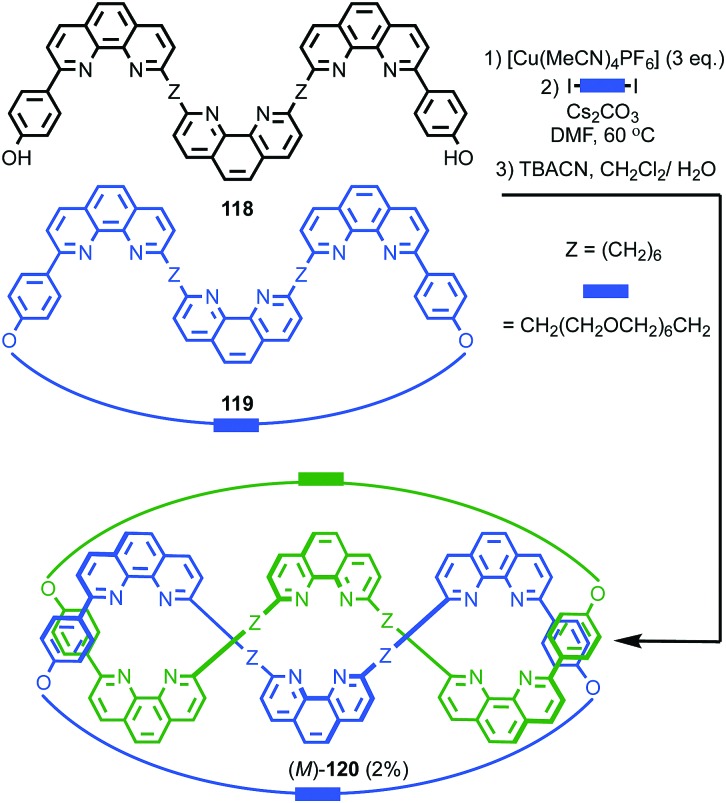
Sauvage's synthesis of molecular Solomon link **120**.^138^

As the chirality of a Solomon link is unconditional, the absolute stereochemistry can be assigned without reference to the molecular structure by considering a representation in which the rings are entwined in a helix, as shown in the case of Solomon link **120** (Fig. 62), and assigning *P* or *M* stereochemistry for a clockwise screw sense and anticlockwise respectively. Alternatively, the helical stereochemistry is directly linked to the pattern of crossing points in the representation shown in Fig. 61; considering the first crossing point in the top left of the structure, if the horizontal line passes over the vertical line this molecule will have a *P* helical representation and if it passes under it will have an *M* helical representation.

The majority of examples of Solomon links have been synthesised in racemic form using methods similar to those used in the synthesis of simple catenanes. Indeed, the first molecular Solomon link was reported by Sauvage and co-workers using their Cu^I^-phenanthroline template motif (Fig. 62).^137^ In this case three phenanthroline units were required in each of pre-macrocycle **118** and preformed macrocycle **119** and the system was designed such that intra-component chelate formation was prevented by rigid linking units. Thus, combination of one equivalent of each macrocycle **119** and pre-macrocycle **118** with three equivalents of Cu^I^ led to the formation of a helicate, intra-component cyclisation of which leads to Solomon link **120**. Demetallation of the crude mixture formed followed by chromatography allowed Solomon link **120** (2%) to be separated from the non-interlocked macrocycle **119** and the corresponding simple [2]catenane (1%).

The catenane and Solomon link **120** were differentiated by their very different ^1^H NMR spectra; whereas that of the catenane is highly symmetrical, the signals arising from **120** are broad at room temperature, becoming sharper as the temperature is raised, consistent with a compact structure in which the two doubly entwined macrocycles have limited conformational freedom. Later work from the same group using the same strategy but replacing Cu^I^ with Li^I^ and employing a Ru^II^-mediated alkene metathesis cyclisation reaction led to an improved 30% yield of the corresponding Solomon link.^138^

**Fig. 63:**
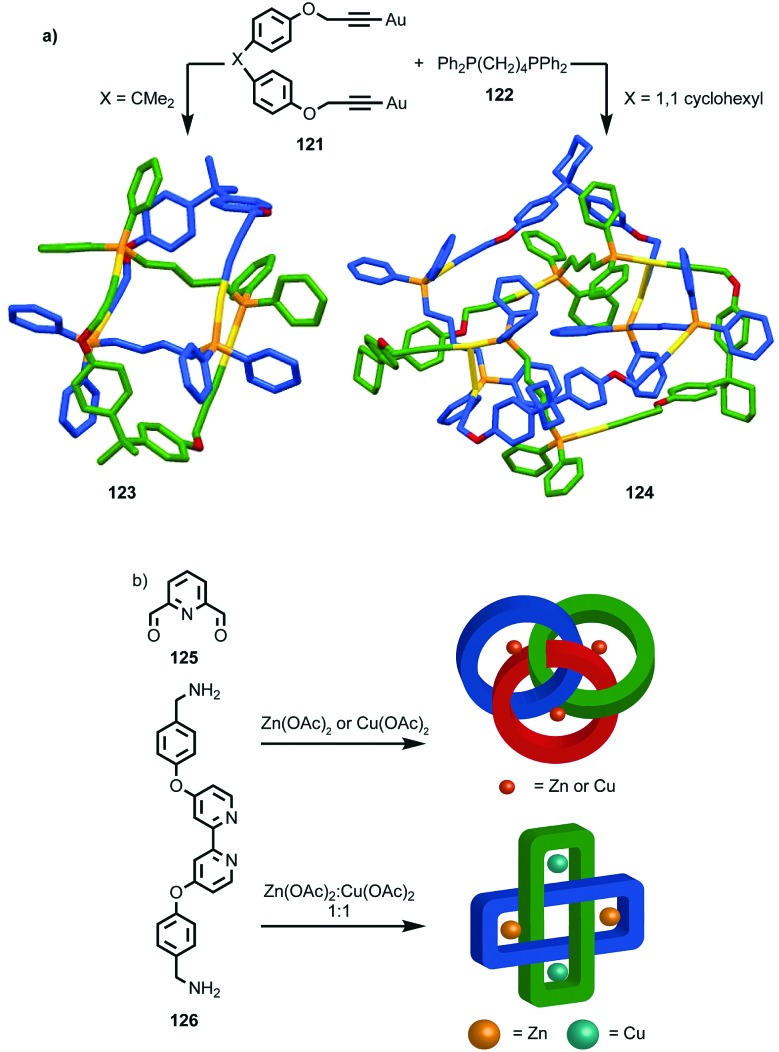
(a) Variation in the outcome of the self-assembly process with small changes in the structure of ligands **121**.^140^ (b) Stoddart's Solomon link synthesis in which choice of metal ions employed leads to formation of a Solomon or Borromean link from the same ligands.^141^ Counter ions omitted for clarity.

These reports from Sauvage and co-workers demonstrate not only the first syntheses of a molecular Solomon link but also the inherent challenge of producing a molecule containing four crossing points. Remarkably, Puddephat and co-workers observed the formation of a Solomon link using the same aurophilic template employed in the synthesis of axially chiral catenanes **86** simply by varying the structure of the ligand employed rather than, as Sauvage and co-workers did, by installing additional templating moieties (Fig. 63).^139^ Indeed, relatively simple structural modifications of the ligand led to either the formation of [2]catenane **123** or the Solomon link **124** presumably due to differences in rigidity and weak secondary interactions.

Similarly, Stoddart and co-workers discovered that varying the metal ion in their Borromean ring synthesis led to formation of either the Borromean rings if Zn^II^ or Cu^II^ were used or the Solomon link if a mixture of Zn^II^ and Cu^II^ were employed together (Fig. 63b).^140^ The origin of selectivity in the latter case was proposed to be a kinetic resolution of the dynamic combinatorial library present in solution, driven by crystallisation. In later work, the same authors found that by varying the solvent they were able to isolate the all-Zn^II^ Solomon link.^141^,^142^

**Fig. 64:**
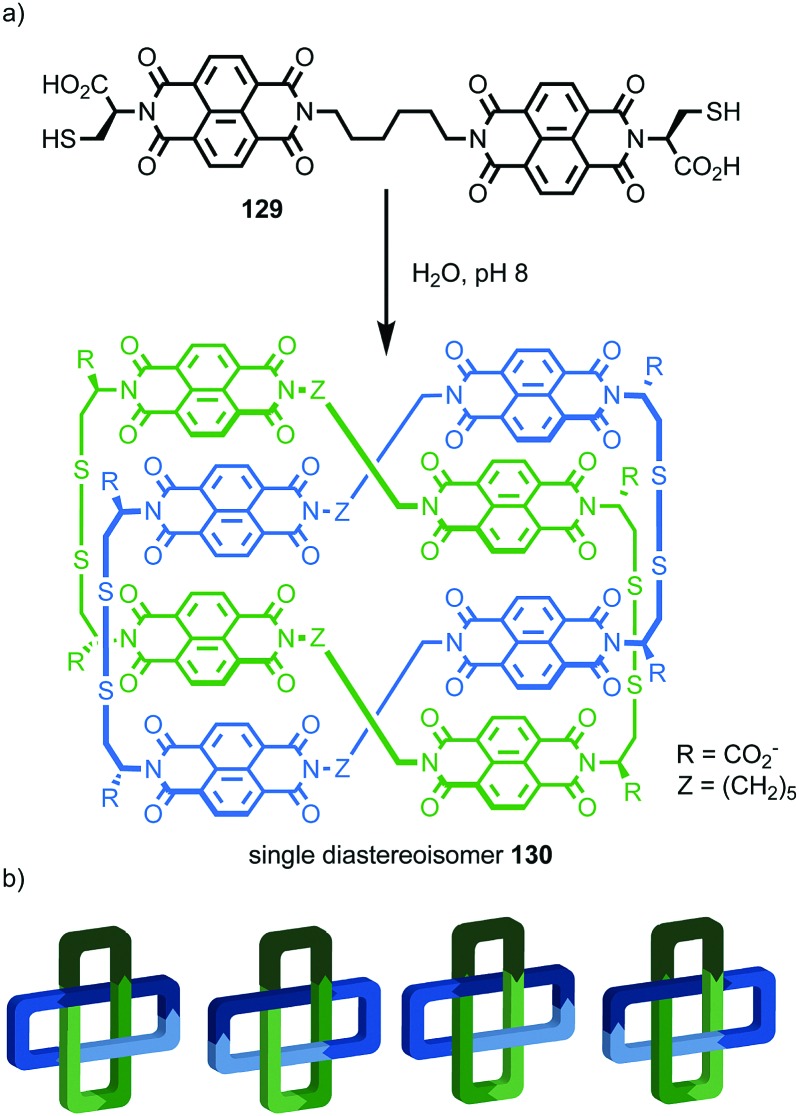
(a) Sanders synthesis of an enantiopure Solomon link **130** using dynamic combinatorial chemistry.^146^ (b) Schematic representation of the conditional topological stereoisomers of one of the enantiomers of **130**. Counter ions omitted for clarity.

Although Solomon links have been synthesised using a number of strategies, including combinations of different templating interactions,^143^ or cyclic metal helicates,^144^ to our knowledge only one example of an enantiopure Solomon link has been disclosed. Sanders and co-workers observed the emergence of a Solomon link from a dynamic combinatorial library containing chiral building block **129** (Fig. 64a).^145^ Slow oxidation of dithiol **129** under air at room temperature in water (5 mM) led to the emergence of a single major product composed of four units of **129**, that was identified as Solomon link **130**.^146^

Only a single isomer of **130** was observed by ^1^H NMR despite the enantiopure stereocentres in the building block, leading to the conclusion that the homochirality of the building blocks directs the formation of a single topological stereoisomer, although the absolute stereochemistry of **130** was not determined. This is even more remarkable given that the two rings of **130** are oriented and thus, in addition to covalent chirality and unconditional topological chirality, Solomon link **130** also displays conditional topological stereochemistry similar to that of topologically chiral catenanes (Fig. 64b). Thus, it appears that the stereocentres in building block **129** direct the formation of a single diastereomer of the 8 available within the library. Since the absolute stereochemistry of Solomon links containing oriented rings has not been determined, or to our knowledge discussed, we have not proposed a method of stereochemical assignment. However, it is likely that a method similar to that discussed above for topologically chiral catenanes would be appropriate.

**Fig. 65:**
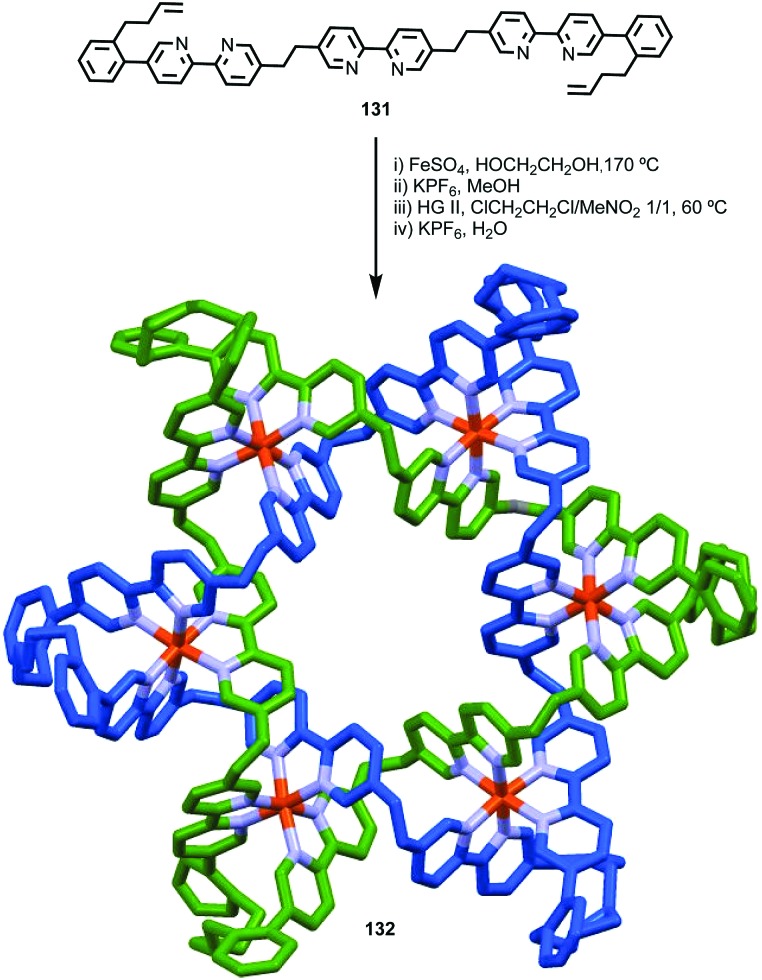
Leigh's synthesis of racemic Star of David catenane **132** from building block **131** by assembly of a circular helicate and ring closing alkene metathesis.^148^ Counter ions omitted for clarity.

The Solomon link is the simplest two component interlocked molecule that displays unconditional topological chirality. Braiding the system further to create a six or greater crossing point catenane would also lead to topologically chiral structures. To date the only example reported is star-of-David catenane **132** synthesised by Leigh and co-workers (Fig. 65).^147^ Building block **131** was employed with six equivalents of Fe^II^ to produce a racemic mixture of circular helicates. Subsequent ring closing alkene metathesis cyclisation produced triply braided catenane **132**. The yield, 69%, over these two steps is remarkable for such a complex molecule.

### Multiply interlocked [*n*]catenanes

Increasing the number of interlocked components can also lead to the appearance of unconditional topological chirality, although only in the case of catenanes, rotaxanes being topologically trivial. In order for the additional components to lead to topological chirality they must be mutually interlocked; a simple [*n*]catenane composed of fully symmetrical *D*_∞h_ rings, whether linear or cyclic, will always be topologically achiral. However, when the rings are mutually interlocked, as in a cyclic [3]catenane in which all of the rings pass through one another a single time, the system is unconditionally topologically chiral.

**Fig. 66:**
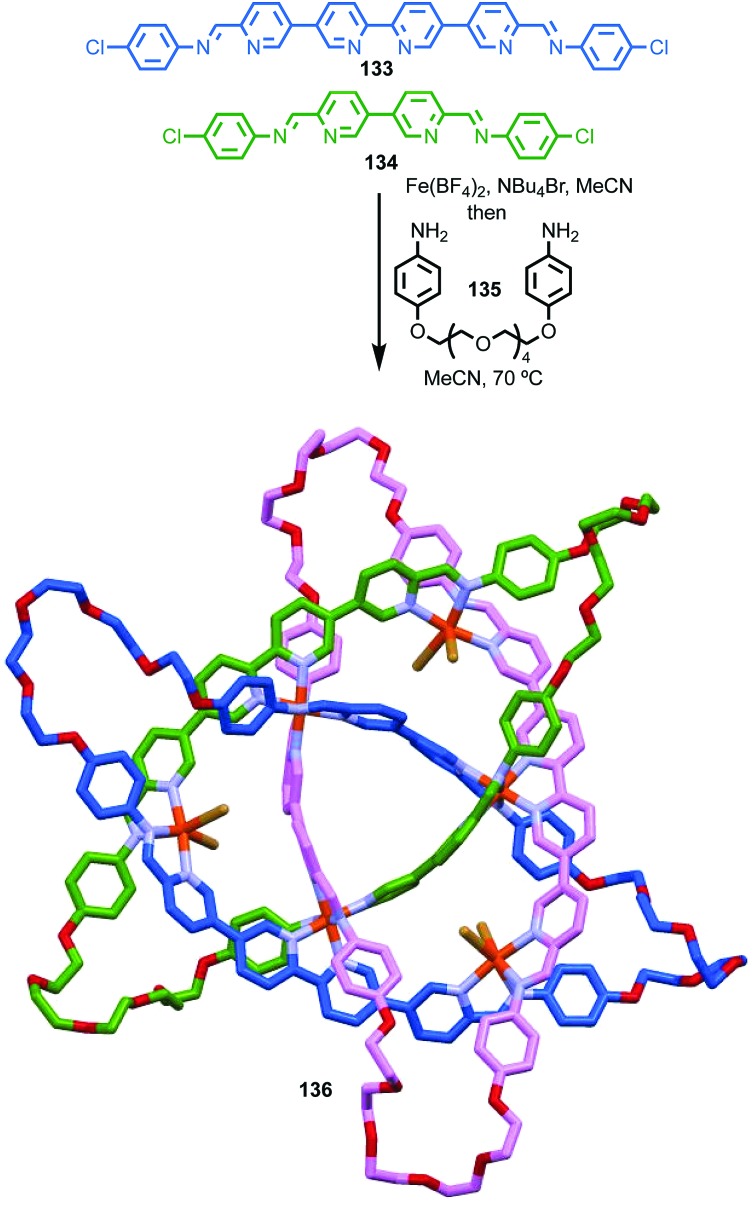
Nitschke's stepwise approach to a triply interlocked [3]catenane **136** displaying unconditional topological chirality.^149^

Unsurprisingly, given their synthetic complexity, examples of such molecules are extremely rare. Nitschke and co-workers successfully synthesised [3]catenane 136 using a stepwise approach (Fig. 66).^148^ First, building blocks **133** and **134** were reacted with six equivalents of Fe^III^ to generate a helicate with the correct crossing points for the target [3]catenane. To capture the interlocked molecule the electron deficient *p*-chloroaniline unit was displaced from the terminal imine of the helicate by tethered diamine **135** to give a cyclic [3]catenane topology. Single crystal X-ray analysis confirmed the target topology had been achieved and also demonstrated the topological chirality of the system; both enantiomers of **135** were observed in the solid state.^149^

**Fig. 67:**
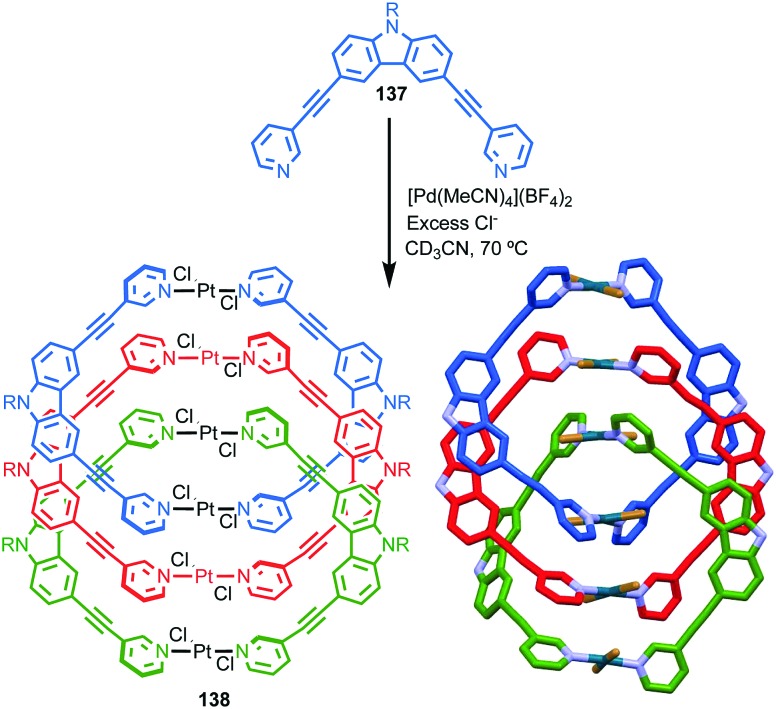
Clever's synthesis of a triply interlocked [3]catenane **138**.^151^ R = 1-hexyl. Hexyl group omitted for clarity.

Clever and co-workers reported a triply interlocked catenane based on metallo-macrocycles and using a dynamic self-assembly strategy (Fig. 67).^150^ Building block **137** assembles into a dinuclear M_2_L_4_ macrocycle in the presence of Pd^II^ and addition of 1.5 equivalents of chloride leads to the formation of a [2]catenane by interpenetration of two rings. Further addition of chloride leads to a triply interlocked [3]catenane **138** in which each macrocycle is linked to both of the others in a cyclic fashion, resulting in unconditionally topological chirality.

**Fig. 68:**
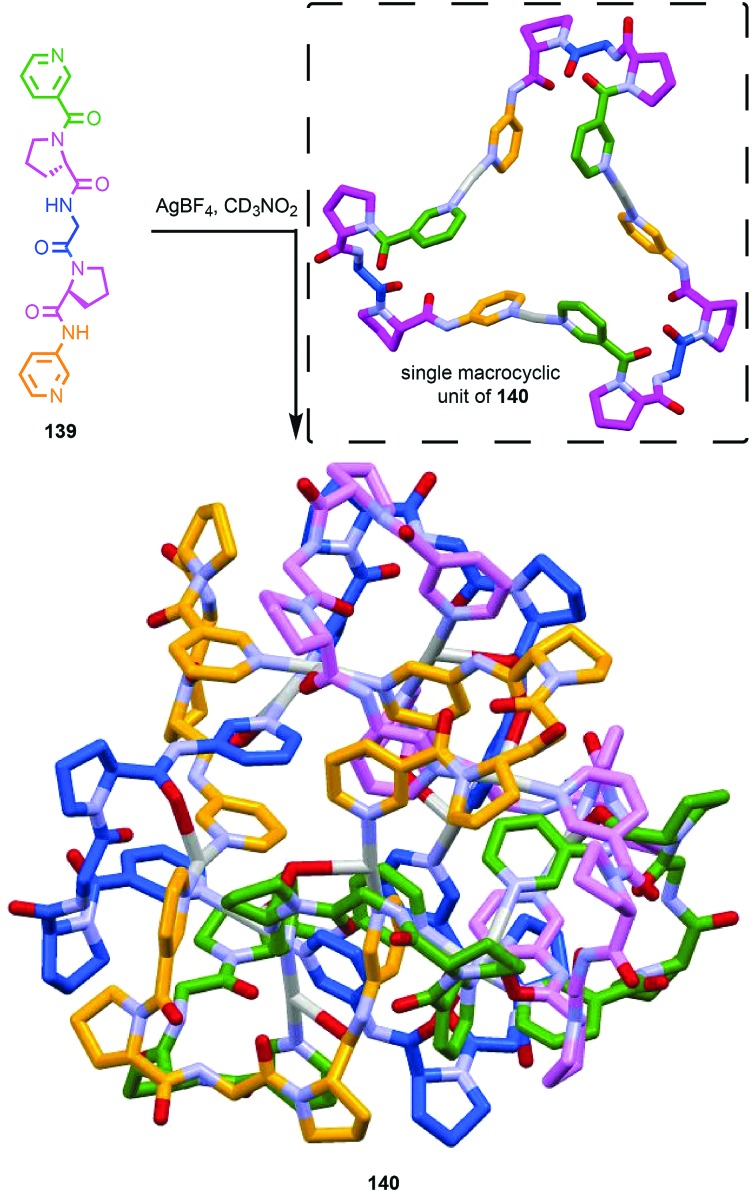
Fujita's stereoselective synthesis of quadruply interlocked [4]catenane **140** from chiral building block **139**.^152^ Counter ions omitted for clarity.

To our knowledge, only one example of a tetra-interlocked cyclic [4]catenane has been reported. In 2016, Fujita and co-workers reported that peptide-based unit **139** assembles in the presence of Ag^I^ or Au^I^ to form a [4]catenane with 12 crossing points composed of four trimeric macrocycles (Fig. 68).^151^ It should be noted that catenane **140** contains multiple opportunities for isomerism in addition to the unconditional topological chirality of the [4]catenane architecture itself; building block **139** is oriented and thus can assemble into the constituent macrocycles in a head–tail or head–head manner, resulting in two possible constitutional isomers of the macrocycle, both of which are overall oriented and contain six fixed stereogenic centres. This raises the possibility of extremely complex stereoisomerism; even ignoring the constitutional and orientational isomerism of the rings, [4]catenane **140** has two possible topological diastereoisomers *M*, *S* and *P*, *S* where *M*/*P* refer to the topological chirality of the catenane and *S* refers to the fixed all-*S* stereochemistry of the constituent building blocks. The oriented nature of the macrocycles adds an element of conditional topological stereochemistry.

Solid state analysis revealed that ligand **139** assembles in a head-to-tail arrangement resulting in a structure has T-symmetry with all of the **139** units equivalent by rotation. However, the solution state ^1^H NMR data reveals two environments for all of the protons associated with the **139** unit. The authors propose this is due to a rhombohedral distortion of the structure in solution that results in loss of the *C*_2_ axes and effective *C*_3_ symmetry. However, they do not comment on the complex stereochemistry of such systems and it is worth noting that their explanation assumes that [4]catenane **140** is formed as a single diastereomer, directed by the fixed stereogenic centres of **139**. An alternative explanation for the lower symmetry observed in solution would be that **140** is present as *M*, *S* and *P*, *S* diastereomers, only one of which crystallises. Further work is required to verify this second hypothesis but it is worth noting that were this to be the case, it remains striking that the covalent stereochemistry of **139** is sufficient to completely control the conditional topological stereogenic unit.

### Conclusions

Although the synthesis of interlocked molecules displaying unconditional topological chirality is challenging – by definition it requires the generation of systems with high crossing numbers – it is clear that modern synthetic methods are approaching a level of sophistication that can tackle such targets. In particular, Solomon link **130** reported by Sanders and co-workers and [4]catenane **139** reported by Fujita and co-workers suggest that covalent stereogenic units can be used to influence the conditional and unconditional topological stereochemistry of these molecules when assembled under thermodynamic control. This parallels recent progress in the synthesis of topologically chiral knots,^133^ including an enantiopure example that has found applications in asymmetric catalysis.^152^,^153^ Hopefully in the coming years we will see more progress in this area both from the point of view of fundamental science and applications.

## The “Missing” chirotopic mechanical stereogenic elements

In preparing this review we considered how best to categorise the existing stereogenic elements that arise as a result of the mechanical bond to generate a useful taxonomy that grouped the reported systems according to their common structural and dynamic features. The result of the process was the categorisation of the reported structures into systems containing covalent, mechanical, co-conformational and unconditional topological chiral stereogenic elements.

A side effect of this process is that it has highlighted a number of “missing” stereogenic units, structures that are obviously possible but have yet to be reported or explicitly highlighted. In this final section, we outline the “missing stereogenic units” we have identified in the hope of stimulating future work to realise them in chemical form. We have tentatively named them, provided hypothetical structures, and in most cases proposed methods for assigning their absolute stereochemistry by analogy with the examples discussed above.

### Co-conformational covalent axial and planar chirality

#### Description of the stereogenic unit

**Fig. 69:**
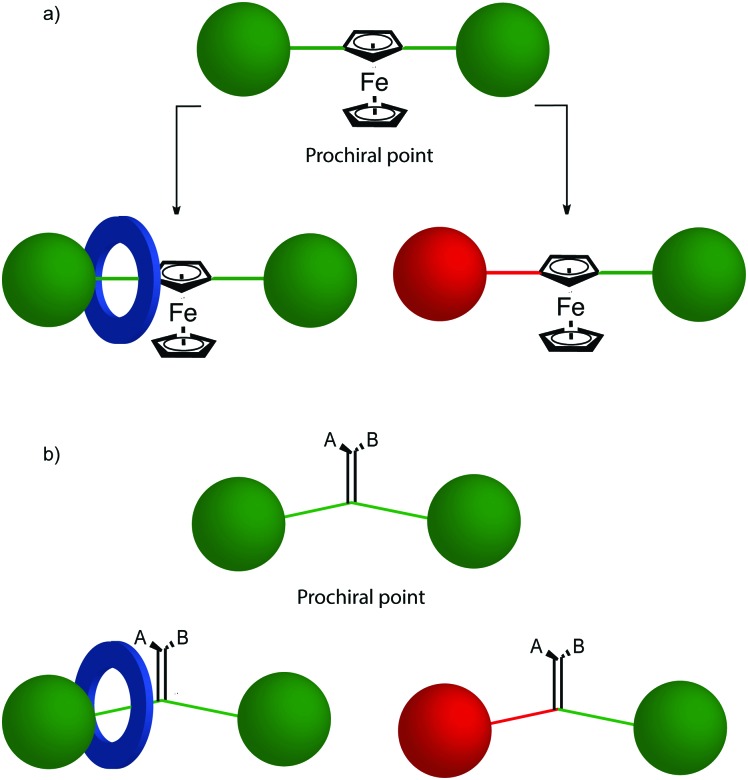
Examples of co-conformational covalent (a) planar chiral and (b) axially chiral stereogenic elements.

The co-conformational covalent point chiral systems described by Leigh and co-workers require the relative positions of mechanically bonded groups to desymmetrise a prochiral stereogenic centre. Although they have yet to be described, the same analysis can clearly be applied to prochiral axial and planar stereogenic units; the localisation of a mechanically bonded component over an enantiotopic substituent will lead to desymmetrisation and the appearance of covalent axial or planar chirality respectively (Fig. 69).

As with the co-conformational covalent point chiral systems discussed above, the axial and planar analogues can either be dynamic in cases where co-conformational motion exchanges the enantiomers, or static, when such motion is blocked, typically by steric hindrance, leading to separable co-conformationally covalently chiral enantiomers. Higher order structures containing multiple interlocked components are also possible and, in the case of [*n*]rotaxanes, the inability of macrocycles to pass one another on the axle typically leads to static co-conformational enantiomers.

#### Assignment of absolute stereochemistry

**Fig. 70:**
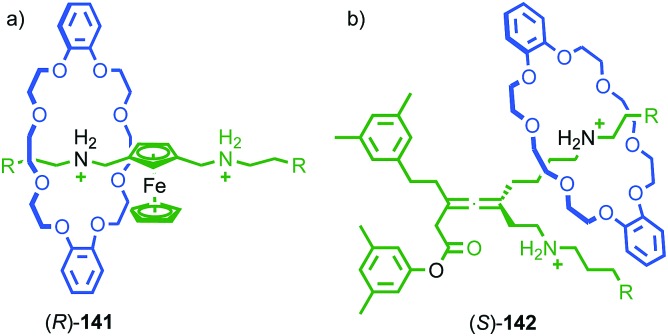
Examples of co-conformationally covalently (a) planar chiral and (b) axially chiral stereogenic elements. Counter ions omitted for clarity.

It is trivial to extend the method of assignment proposed above for co-conformationally covalent point chiral molecules to their axial and planar analogues. Indeed, the method above is worded such that it is applicable to any prochiral covalent stereogenic element simply by applying the appropriate accepted methodology for a stereogenic axis or plane. To demonstrate this approach hypothetical structures **141** and **142** have been assigned and labelled (Fig. 70).

**Fig. 71:**
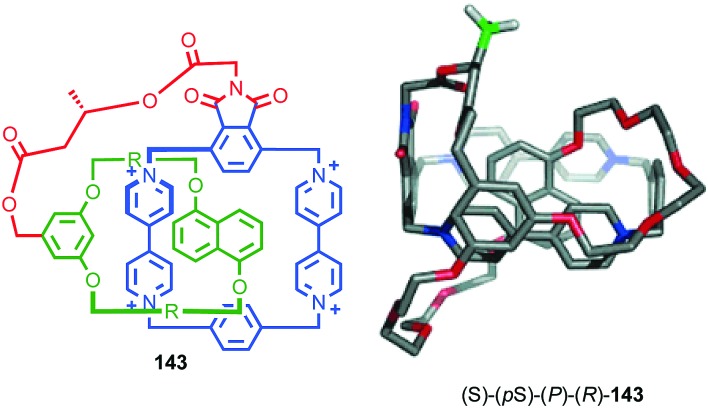
Stoddart's pretzelane **143** that displays bias between enantiomeric (co)conformations due to a single fixed covalent stereocentre and its modelled structure, showing the predicted preferred (co)conformation.^155^ The assigned stereochemistry is listed in the order covalent point, conformational planar, co-conformational covalent planar, co-conformational mechanical helical. R = (CH_2_OCH_2_)_3_. Reproduced from ref. 154 from The Royal Society of Chemistry.

Although co-conformational covalent axial and planar stereogenic elements have not previously been highlighted, pretzelane **143**, reported by Stoddart and co-workers, can be described as containing a co-conformational covalent planar chiral stereogenic unit (Fig. 71).^154^ Pretzelane **143** contains a fixed stereogenic centre, a conformationally stereogenic plane (dialkoxynaphthalene), a dynamic element of mechanical helical chirality (rocking of the two macrocycles relative to another) and a co-conformational covalent planar stereogenic element (the imide ring). Despite the potential for eight distinguishable stereoisomers, ^1^H NMR suggests only two are significantly populated in solution. By analogy with an achiral analogue lacking the fixed stereocentre and molecular modelling, the preferred co-conformations of **143** were proposed to be (*S*,*S*,*P*,*R*) (Fig. 71) and (*S*,*R*,*M*,*S*) with the former being significantly preferred in CD_3_CN (9 : 1). The kinetic barrier to exchange between the diastereomers was found to be 74.5 kJ mol^–1^ at 301 K in *d*_6_-DMSO which is in keeping with the barrier to pirouetting of the rings relative to one another. Analysis of the CD spectra of **143** revealed strong Cotton effects in the region of the spectrum associated with the bipyridinium moiety and the charge transfer band between the bipyridinium and dialkoxynaphthalene units, consistent with the fixed stereocentre imposing a chiral environment on the chromophores through a biased co-conformation.

#### Conclusions

As with co-conformational covalent point chirality, co-conformational covalent planar and axial chirality seem likely to be amenable to standard techniques from asymmetric synthesis. Given the importance of both classical covalent planar and axial chirality in catalysis, sensing and materials applications, combined with Leigh's demonstration of co-conformational covalent point chirality in catalysis, these forms of stereochemistry seem ripe for exploitation. Our hope is that by explicitly highlighting them we will stimulate activity in this area.

### Co-conformational “Topologically” chiral catenanes

#### Description of the stereogenic element

**Fig. 72:**
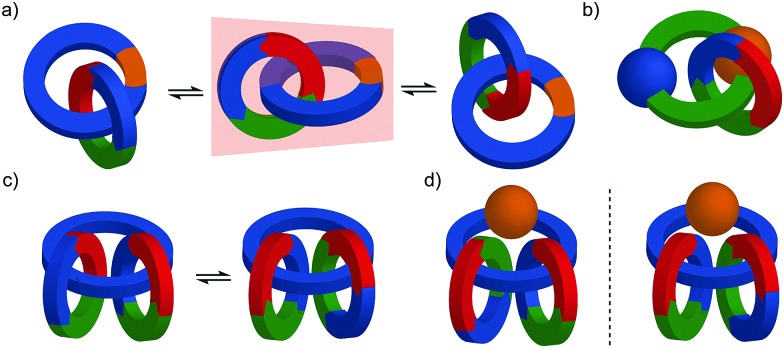
Co-conformational topological chirality. (a) Pirouetting between enantiomers in a [2]catenane. (b) A co-conformationally “topologically” chiral [2]catenane locked in a single enantiomeric co-conformation. (c) Exchange between enantiomers in the *anti* diastereomer of [3]catenane containing oriented peripheral rings. (d) A co-conformationally “topologically” chiral [3]catenane locked in a single enantiomeric co-conformation.

Although it has not previously been highlighted, in a similar manner to co-conformational mechanical planar chirality, it is possible to produce enantiomers with the symmetry properties of topologically chiral catenanes even without meeting the requirement of two *C*_*n*h_ oriented rings.^155^ When a catenane is formed from an oriented *C*_*n*h_ ring (principle axis perpendicular to the macrocycle plane) and a *C*_2v_ un-oriented macrocycle (principle axis in the plane of the macrocycle), its co-conformation will be achiral whenever the oriented ring lies on the mirror plane of its partner (Fig. 72a). However, pirouetting of the oriented ring either side of the mirror plane results in enantiomeric co-conformations. If the barrier to the pirouetting process is high enough, for instance in the presence of blocking groups (Fig. 72b), these enantiomers will be separable as in the case of co-conformationally mechanically planar chiral rotaxanes.

Furthermore, introducing multiple rings, at least one of which is oriented onto a fully symmetrical, *D*_∞h_ central ring to form a linear [3]catenane leads to emergence of co-conformational topological chirality (Fig. 72c). If the oriented peripheral rings are identical the *anti* topological diastereomer (Fig. 34c) can exist as co-conformational enantiomers whenever the peripheral rings are not disposed on a mirror plane. The *syn* diastereomer is achiral in all co-conformations if the two oriented rings are identical but has enantiomeric co-conformations if the oriented rings are distinguishable. These stereochemical properties bear some similarity to the co-conformational mechanical planar chirality of [3]rotaxanes composed of an unoriented axle and oriented macrocycles (Fig. 47). Introducing a blocking group in the *anti* diastereomer results in separable co-conformational “topological” enantiomers (Fig. 72d). Increasing the number of rings, as in the case of mechanically planar chiral rotaxanes results in more stereoisomers.

#### Assignment of absolute stereochemistry

As they have not previously been discussed explicitly there is no established method for assigning the absolute stereochemistry of co-conformational “topologically” chiral catenanes. We propose the following approach to the assignment of absolute stereochemistry which is in keeping with the methods developed earlier for co-conformational mechanically planar chiral rotaxanes. For each oriented macrocycle in turn:

(i) Determine the orientation of the macrocycle as for simple topologically chiral catenanes.

(ii) Assign the relative priority of any other relevant macrocycles by comparing their highest priority constituent atoms according the CIP rules. By convention, the macrocycle under consideration is assigned the lowest priority.

(ii) Where more than one such component is present and the system is co-conformationally dynamic (*i.e.* catenane **145**), the macrocycles are considered in an arbitrary co-conformation in which they are evenly distributed either side of the mirror plane associated with the central unoriented macrocycle. Where the system is not dynamic (*i.e.* the components are trapped in a preferred co-conformation) or where the stereochemical assignment of a specific co-conformation is of interest (*i.e.* catenane (*S*)-**144**) they are considered localised on the appropriate side of the mirror plane associated with the central macrocycle.

(iii) The interlocked components are added in turn, in order of priority, as substituents of the highest priority atom of the region they encircle. This process is repeated until the orientation of the second macrocycle can be assigned unambiguously.

(iv) Using the orientation of the macrocycle determined in (iii), the absolute stereochemistry associated with the oriented ring under consideration is assigned as above for topologically chiral catenanes. This process is then repeated for other oriented rings.

**Fig. 73:**
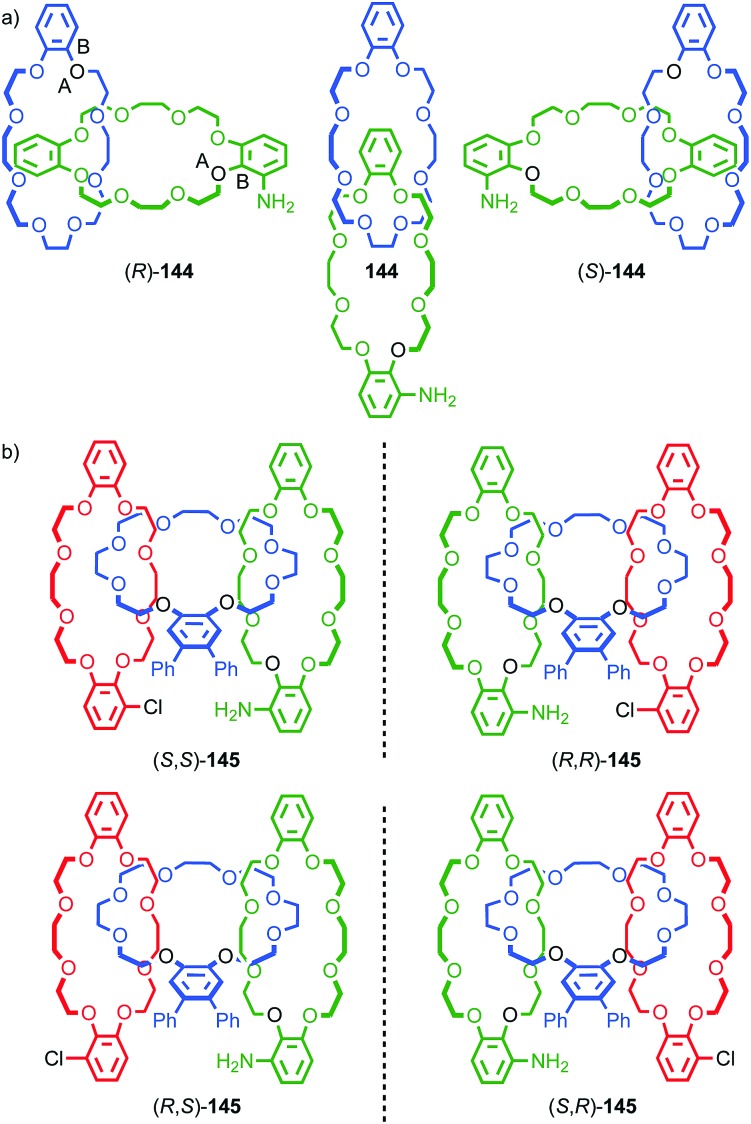
Hypothetical examples of co-conformationally “topologically” chiral catenanes and their stereoisomers. (a) Dynamic co-conformationally “topologically” chiral catenane **144** and its co-conformations. (b) Locked co-conformationally “topologically” chiral catenane **145** and its enantiomers. The highest priority atom(s) in each component are highlighted in black. The stereochemical labels of **145** are ordered according to the priority of the macrocycles (*i.e.* the red ring stereodescriptor is first in all cases).

As, to our knowledge co-conformationally “topologically” chiral catenanes have not been reported, to demonstrate this approach hypothetical structures **144** and **145** have been assigned and clearly labelled (Fig. 73).

#### Conclusions

Given that they have yet to be investigated at all there is clearly potential for new and interesting properties and applications of these molecules to be discovered. It may also be that co-conformationally “topologically” chiral catenanes are synthetically easier to access than their conditionally topologically chiral analogues using kinetic resolution, catalytic desymmetrisation or other strategies from asymmetric synthesis.

From the point of view of nomenclature this is the most troubling section of the review; co-conformational stereogenic elements by definition cannot be topological as they can be exchanged by the relative motion of the covalent subcomponents,^10^ hence the quotation marks that have been used consistently throughout this section. However, given the relationship between the symmetry properties of these co-conformational examples and “true” topologically chiral catenanes it seems sensible to link the nomenclature of these stereochemical phenomena. One solution to this linguistic problem would be to re-categorise topologically chiral catenanes as mechanically planar chiral catenanes, in light of the obvious similarities between this stereogenic element and that of mechanically planar chiral rotaxanes. However, this is a decision that needs to be taken by the community and could cause confusion by creating a disconnect between future work and the systems described over the last three decades. For now, we will leave the linguistic problem in place and hope to stimulate discussion in the community.

### Co-conformationally mechanically axially chiral catenanes

#### Description of the stereogenic unit

Whereas it is relatively trivial to envisage how co-conformational chirality can arise when the relative position of the mechanically bonded subunits lowers the internal symmetry of a covalent prochiral unit (as in the cases of co-conformational covalent chirality), or the regions of one of the interlocked components (as in the cases of co-conformational mechanical planar and “topological” chirality), it is harder to intuitively see how co-conformational mechanical axial chirality can arise.

**Fig. 74:**
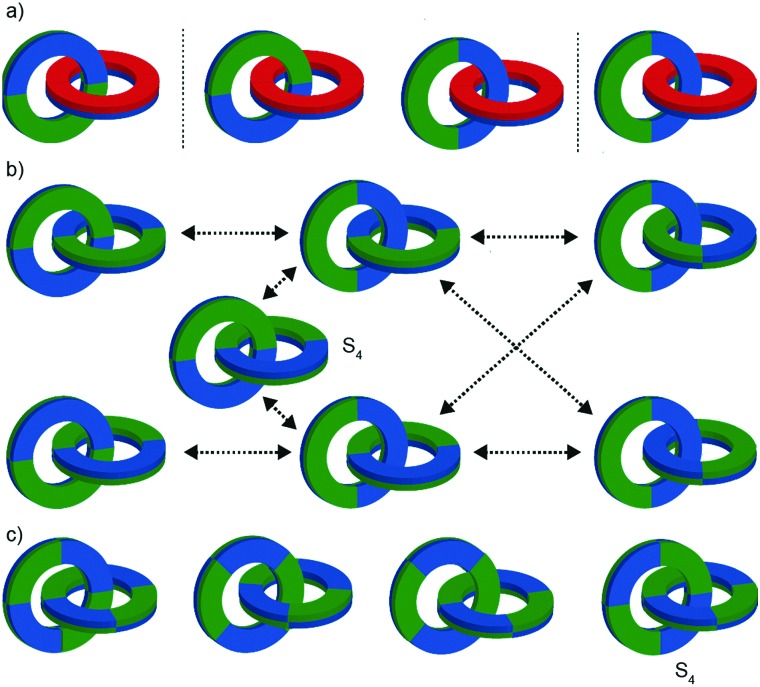
Schematic representations of co-conformational mechanical axial chirality. (a) Stereoisomers of a [2]catenane composed of one *C*_2h_ and one *C*_∞v_ macrocycle. (b) The network of stereoisomers formed by a [2]catenane based on two *C*_2h_ macrocycles (each arrow corresponds to a 90° rotation of one ring; vertical pairs of structures are related as enantiomers; achiral diastereomer is indicated as S_4_ symmetry). (c) The four diastereomers of a [2]catenane based on two *D*_2d_ macrocycles (enantiomers not shown; achiral diastereomer is indicated as S_4_ symmetry).

However, examining schematic representations of a hetero[2]catenane composed of a *C*_2h_ ring (principle axis parallel to the macrocycle plane) and one *C*_*n*v_ macrocycle (principle axis perpendicular to the ring plane), reveals the possibility of enantiomeric co-conformations that are exchanged by pirouetting of the components (Fig. 74a). Although this form of co-conformational chirality has not previously been identified or classified, the stereogenic unit relies on the facial symmetry properties of the two macrocycles and so we propose that these molecules are classified as co-conformationally mechanically axially chiral by analogy with static mechanical axial chirality introduced above. More generally, the co-conformational mechanical axial stereogenic element arises in any [2]catenane composed of at least one *C*_2h_ or *D*_*n*d_ (principle axis perpendicular to the ring, *n* is an even integer) ring combined with a *C*_2h_ (Fig. 74b), *D*_*n*d_ (Fig. 74c) or *C*_*n*v_ macrocycle.

The co-conformational stereochemical behaviour of such systems bears some comment. The *C*_2h_/*C*_∞v_ [2]catenane gives rise to two diastereomeric pairs of enantiomers that are exchanged through a 180° circumrotation of the *C*_2h_ without passing through an achiral arrangement. Hence, although the enantiomers are in equilibrium, all co-conformations are chiral (Fig. 74a). In contrast the *C*_2h_/*C*_2h_ homo[2]catenane has four diastereomeric limiting co-conformations, one of which has S_4_ symmetry and is achiral and the other three exist as enantiomers (Fig. 74b). However, although it is possible to exchange the co-conformational enantiomers *via* the achiral S_4_ diastereomer, the shortest path between enantiomers is *via* chiral co-conformations for two of the three chiral diastereomers. Similar behaviour is found for the *D*_2d_/*D*_2d_ catenane.

#### Synthesis of co-conformational mechanically axially chiral catenanes

**Fig. 75:**
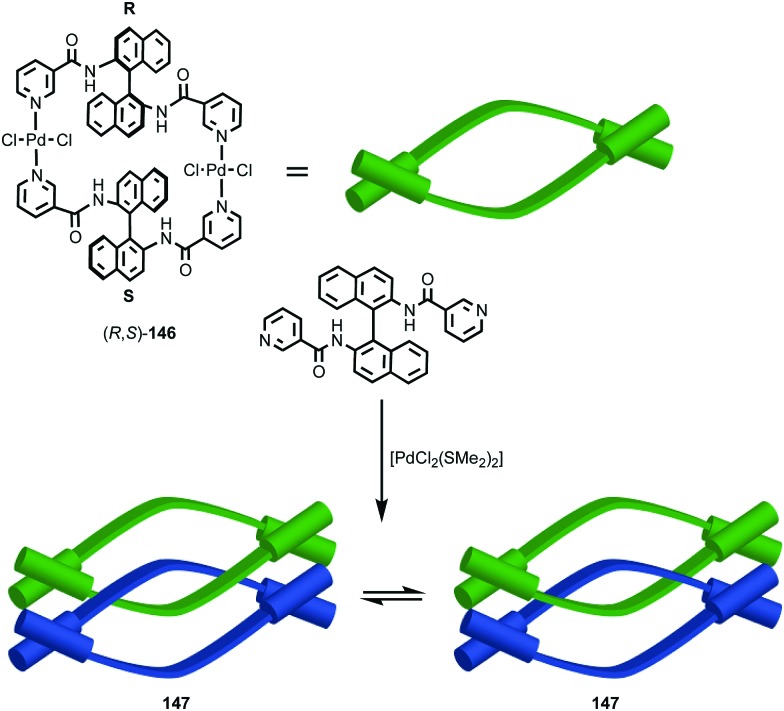
Puddephat's mechanically axial chiral catenanes **147**.^157^

Although the co-conformational mechanical axial stereogenic unit has not been identified previously, during the preparation of this review we identified catenane **147**, reported by Puddephat and co-workers, which conforms to the symmetry requirements outlined above. Catenane **147** is formed through the assembly of four equivalents of racemic building block **146** in the presence of Pd^II^ (Fig. 75).^156^ X-ray analysis of **147** reveals that, although three stereoisomeric macrocycles can be formed from (*R*/*S*)-**146**, in the solid-state catenane **147** is composed exclusively of macrocycles with (*R*,*S*) absolute stereochemistry that individually have *C*_2h_ symmetry and are thus achiral when not interlocked. However, catenane **147** adopts an axially chiral co-conformation in the solid-state and both enantiomers were present in equal amounts in the unit cell.

^1^H NMR analysis of catenane **147** revealed four distinct NH resonances, consistent with the *C*_2_ symmetry of the solid state chiral co-conformation. However, at 298 K cross peaks were observed between pairs of NH environments by COSY NMR analysis which are absent at 273 K, suggesting that a dynamic process exchanges these positions. This pairwise exchange process is consistent with the pirouetting of the rings which exchanges pairs of enantiotopic protons.

#### Conclusions

Although the co-conformational mechanical axial chiral stereogenic unit has not been commented on in general terms before, catenane **147** clearly demonstrates the principles of this stereogenic element. Now that it has been highlighted, and in particular, the relatively unusual stereochemical course of the co-conformational stereodynamic processes such molecules can undergo, we hope that this will receive more attention in future. The keen reader will also notice that we have omitted to propose a method for assigning the absolute stereochemistry of co-conformationally mechanically axially chiral enantiomers. This is because there is no obvious way to go about it! Given the dearth of examples and the unusual nature of the one example reported we have elected to leave this challenge until more structures are disclosed it is clearer how to tackle the problem.

### Conclusions – the missing elements of mechanical chirality

Perhaps unsurprisingly, the missing stereogenic elements identified are co-conformational rather than conditional, the majority of the latter having been identified over 20 years ago. However, although these stereodynamic systems are perhaps slightly esoteric, the potential for such stereoisomerism is important not only for the analysis of interlocked molecules, where chiral co-conformations may result in complex NMR spectra, but also from the point of view of applications; Leigh has already demonstrated that co-conformational covalent point chiral molecules can be used in asymmetric catalysis, which begs the question, what are the potential benefits of the analogous co-conformational covalent planar or axial stereogenic units? Similarly, co-conformational “topologically” and mechanically axially chiral catenanes are completely unexplored. It might be that their stereodynamic nature could make their synthesis and desymmetrisation to give enantiopure samples easier than the corresponding static stereogenic elements, for instance by dynamic kinetic resolution.

## Conclusions

**Fig. 76:**
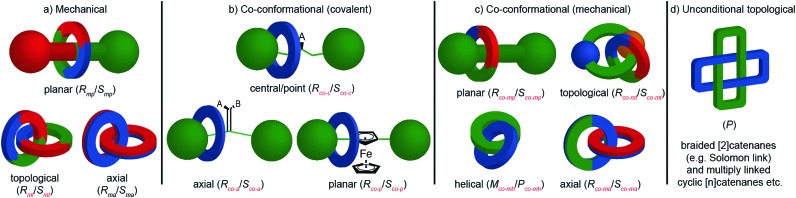
A summary of stereogenic units that arise from the mechanical bond categorised by (a) conditional, (b) co-conformational covalent, (c) co-conformational mechanical and (d) unconditional stereochemistry. Stereodescriptors and suffixes used in stereochemical labels are shown (proposed stereochemical suffixes in red).

In this review, we have presented the major stereogenic elements that have been incorporated and observed in interlocked molecules with a particular focus on those that arise as a result of the mechanical bond (Fig. 76), and the properties of the resulting chiral interlocked structure. We have also highlighted applications of chiral interlocked molecules in a range of areas including catalysis, sensing and circularly polarised luminescence. Given the interesting properties of chiral rotaxanes and catenanes and the recent surge in interest in their applications it seems certain that the field will grow rapidly in the next few years.

In preparing this review we took a “first principles” approach to the logical classification of the different possible stereogenic elements according to the structural factors that lead to the appearance of the stereogenic unit. Ultimately this led us to classify the different chiral stereogenic elements present in interlocked structures as covalent, mechanical (Fig. 76a), co-conformational (a category that we further subdivided between covalent and mechanical stereogenic elements Fig. 76b and c respectively) and unconditional topological (Fig. 76d). In places this suggested taxonomy differs from previous comments in the area.

Similarly, in places we have proposed slight modifications to previous approaches to the assignment of absolute stereochemistry for the stereogenic units discussed, or where no clear guidelines exist, made suggestions of how this could be achieved systematically. Also, previous authors have proposed adding suffixes to the stereodescriptor of the mechanical stereogenic unit (Fig. 76; accepted stereodescriptors and suffixes shown in black). In some cases, no suffix has been proposed and here we take the opportunity to suggest suitable examples (Fig. 76; proposed suffixes shown in red).

Our suggested modifications to the taxonomy, methods of assignment and stereolabels of mechanical stereogenic units are obviously just that, suggestions. We hope our approach will prove useful but given that the field is still evolving, look forward to further discussion and revision as these molecules become more prevalent and others begin to grapple with the challenge they pose both in terms of nomenclature and synthesis.

Our discussion of mechanical stereogenic elements has mainly focussed on examples that have been previously identified and described. However, we have also added “missing” elements where they are obviously needed, for example the axial and planar stereogenic equivalents of previously disclosed co-conformational covalent point stereogenic systems and co-conformational “topologically” and mechanically axially chiral catenanes, which are logical analogues of co-conformationally planar chiral rotaxanes which have been disclosed by various authors.

However, it is important that the reader does not regard this review as describing all the stereogenic elements of that can arise as a result of the mechanical bond; it is likely that there are more, particularly in the unconditional topological class that will appear as the complexity in terms of number of components and crossing points rises. To convince the reader of this, our final figure presents three examples that lie outside of the classifications above but nonetheless rely on the mechanical bond for their stereochemical complexity.

**Fig. 77:**
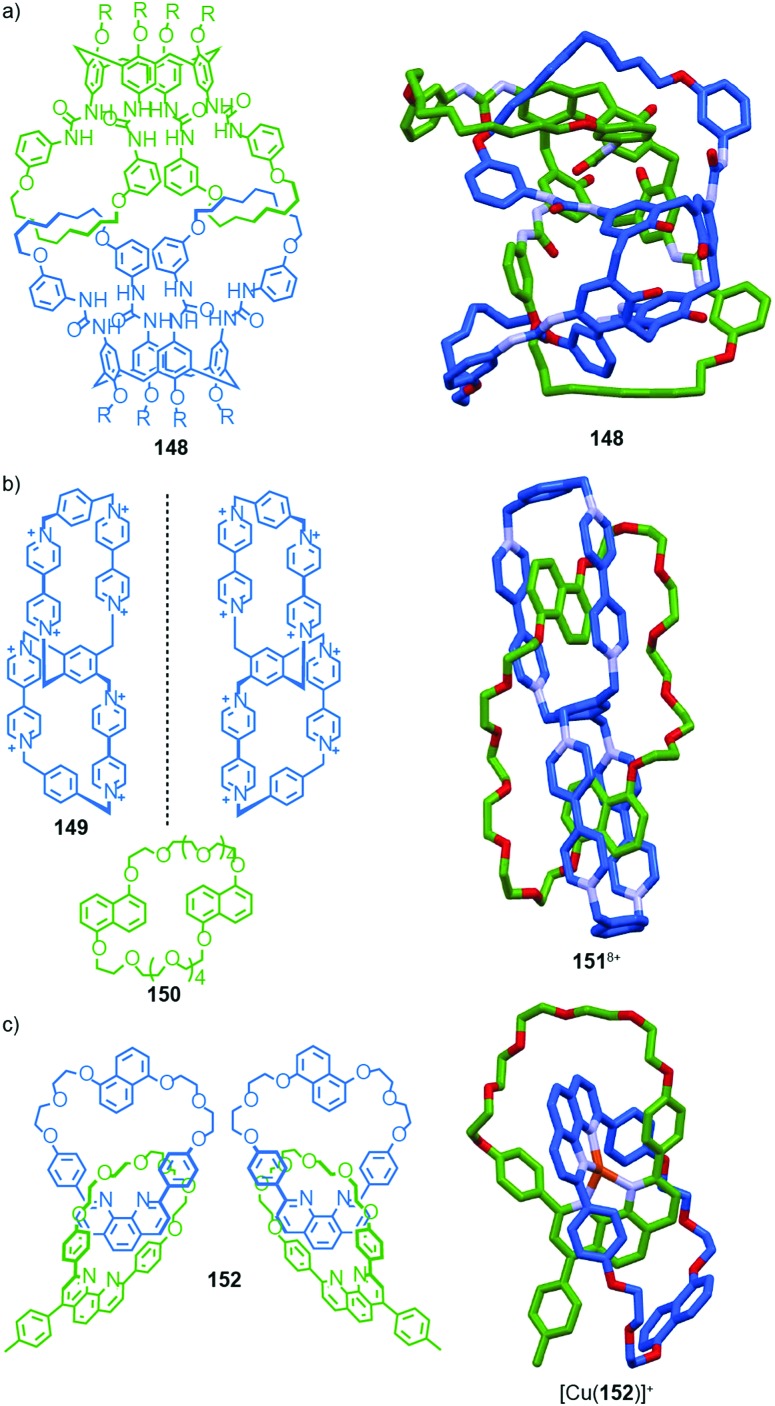
(a) Böhmer's chiral doubly interlocked [2]catenane **148** (R = Et) and its solid state structure (Et omitted for clarity).^158^ (b) Topological stereoisomers of Stoddart's chiral handcuff catenane **150**.^159^ (c) Sauvage's topological rubber glove catenane **151**.^160^

Böhmer and co-workers have disclosed several examples of what are technically [2]catenanes (*i.e.* they contain two covalent subcomponents) but which have two identifiable mechanical bonds (Fig. 77a).^157^ Catenane **148** is chiral as a result of the mechanical bond and the stereogenic element appears similar to co-conformational helical chirality but in this case the rings are unable to reorient by rocking due to the covalent link between them. As a result, catenane **148** is unconditionally topologically chiral.

Another unusual example from the Stoddart group illustrates the potential for the mechanical bond to lead to additional stereoisomers in an already chiral covalent framework (Fig. 77b).^158^ Handcuff catenane **149** contains a rigid planar chiral core with two cationic cyclophane rings projected from the central benzene ring which represents a planar stereogenic element. When a second ring is threaded through both portals of cyclophane **149** a [2]catenane is formed which displays an additional level of stereochemical complexity; depending on the direction of threading of the second macrocycle (which has *C*_2h_ symmetry in its highest symmetry conformation) through each of the enantiomers, two diastereomers can be formed, the enantiomers of which can be formed starting from the other enantiomer of **149**. Once again, these stereoisomers are topological in nature rather than Euclidean as they cannot be exchanged without breaking and reforming the alkoxy naphthalene macrocycle. It is not immediately obvious how they are related to the general classes developed above but they bear some resemblance to the unconditionally topologically chiral [3]catenanes reported by Clever and Nitschke but in which two of the macrocycle have become fused.

Finally, Sauvage and co-workers have disclosed catenane **152** which they described as a “topological rubber glove” (Fig. 77c).^159^,^160^ Neither of the rings in catenane **152** are themselves chiral, but one contains a dialkoxynaphthalene unit that can function as a conformational planar stereogenic element and the other is oriented with *C*_1h_ symmetry. When they are interlocked in catenane **152**, the resulting ensemble is chiral but the enantiomers can be exchanged by rotation of the naphthalene unit between the two enantiomeric conformations. Strikingly, however, at no point in the rotation of the naphthalene moiety between enantiomeric conformations does the system adopt an achiral conformation as the oriented nature of the second macrocycle means there is no mirror plane parallel with the dialkoxynaphthalene ring. Thus, although catenane **152** contains stereogenic elements that are similar to those discussed above, and its stereochemistry can be adequately described by assigning the stereochemistry of the naphthalene unit in the two conformations, its behaviour is quite exotic.

The purpose of these final examples is to highlight the sheer stereochemical diversity that is available when considering interlocked structures and avoid the previous sections of this review giving the impression of completeness; we have merely attempted to map out the majority of known structures and present them in a logical manner. Some of the more complex systems available will remain curiosities with no practical application and it is certainly possible that many possible stereochemically complex interlocked molecules, even those composed of relatively small numbers of components, will never be realised. However, given the central role of chirality in chemistry and potential applications of new chiral stereogenic units in a range of areas, it is also certain that interlocked molecules will continue to be a rich playground for chemists searching for new chiral molecules with unusual properties. With the advent of ever more powerful synthetic methods and the renewed interest the field has received in the last decade, this will undoubtedly remain an exciting topic for both supramolecular chemists and stereochemical enthusiasts.

## Conflicts of interest

The authors declare they have no conflicts of interest.
